# Expression of *Dux* family genes in early preimplantation embryos

**DOI:** 10.1038/s41598-020-76538-9

**Published:** 2020-11-10

**Authors:** Kenta Sugie, Satoshi Funaya, Machika Kawamura, Toshinobu Nakamura, Masataka G. Suzuki, Fugaku Aoki

**Affiliations:** 1 Department of Integrated Biosciences, Graduate School of Frontier Sciences, The University of Tokyo, Seimei-Building 302, 5-1-5 Kashiwanoha, Kashiwa, 277-8562 Japan; 2 Department of Bio-Science, Nagahama Institute of Bio-Science and Technology, Nagahama, Shiga 526-0829 Japan

**Keywords:** Developmental biology, Zoology

## Abstract

After fertilization, the zygotic genome is activated through two phases, minor zygotic activation (ZGA) and major ZGA. Recently, it was suggested that DUX is expressed during minor ZGA and activates some genes during major ZGA. However, it has not been proven that *Dux* is expressed during minor ZGA and functions to activate major ZGA genes, because there are several *Dux* paralogs that may be expressed in zygotes instead of *Dux*. In this study, we found that more than a dozen *Dux* paralogs, as well as *Dux*, are expressed during minor ZGA. Overexpression of some of these genes induced increased expression of major ZGA genes. These results suggest that multiple *Dux* paralogs are expressed to ensure a sufficient amount of functional *Dux* and its paralogs which are generated during a short period of minor ZGA with a low transcriptional activity. The mechanism by which multiple *Dux* paralogs are expressed is discussed.

## Introduction

In mouse oocytes, genes are actively transcribed during the growth phase, and then silenced at the end of their growth^1^. This transcriptionally inactive state remains after fertilization. Zygotic genome activation (ZGA) is initiated at the mid to late S-phase of the 1-cell stage^2^. ZGA proceeds in two phases, minor and major ZGA, and the pattern of gene expression is dramatically changed between these two phases. During minor ZGA, which occurs from the S phase of the 1-cell stage to the G1 phase of the 2-cell stage, a relatively low level of transcription occurs in a large part of gene and intergenic regions, and regions that code for retrotransposons. However, during a subsequent activation that occurs up to the G2 phase of the 2-cell stage, i.e., major ZGA, the number of transcribed genes and transcription from intergenic regions decrease, whereas the expressions of particular genes are greatly increased^3,4^. Abe et al. (2018)^5^ showed that minor ZGA is a prerequisite for the occurrence of major ZGA. In that study, after the temporal inhibition of minor ZGA by 5,6-dichloro-1-β-d-ribofuranosyl-benzimidazole (DRB), a reversible inhibitor of RNA polymerase II, transcription was initiated in the pattern of minor ZGA at the time when major ZGA normally occurred. However, it remains to be elucidated how minor ZGA regulates major ZGA.

Recently, it was suggested that *Dux* is transcribed during minor ZGA and regulates the expression of some genes during major ZGA^6–8^. When *Dux* expression is induced in myoblasts and mouse embryonic stem cells (mESC), hundreds of major ZGA genes are upregulated^7,8^. *Dux* knockout reduces the expression of some of major ZGA genes in 2-cell stage embryos^6^. In addition, microinjection of cRNA encoding *Dux* into blastomeres of late 2-cell stage embryos results in the arrest of embryos at the 4-cell stage^9^, suggesting that transient expression of *Dux* is necessary during minor ZGA to induce the transcription of some of major ZGA genes. In *Dux*-knockout mice, viable offspring can be obtained but the litter size is small^6,10,11^.

In humans, *DUX4*, which is considered an ortholog of mouse *Dux*, is a gene that is found within tandem repeats consisting of *DUX4* paralogs that differ by only a few bases from each other^12^. *DUX4* is localized at the telomere end of the repeats. These tandem repeats are located at pericentromeric regions that are usually heterochromatinized, which prevents their expression in most types of cells. *DUX4* is the causative gene for facioscapulohumeral muscular dystrophy (FSHD), the third most common muscular dystrophy^13–17^. In this disease, only *DUX4* is aberrantly expressed from the tandem repeats^18,19^. In mice, *Dux* is also known to be a gene within tandem repeats and some of its paralogs have been identified^20^: in this study, *Dux* and *Dux* paralogs are collectively called *Dux* family. However, it is not known where *Dux* is located in the tandem repeats.

To date, several *Dux* family genes have been identified, but they are not expressed in early embryos. To identify the function of the *Dux* family and the mechanism regulating their expressions in preimplantation embryos, we first need to identify the *Dux* family gene(s) that is (are) expressed. Here, we report that some *Dux* family genes are expressed and function in early mouse pre-implantation embryos.

## Results

### Diversity of *Dux* paralogs in mouse genome and their expression in early preimplantation embryos

To identify the paralogs of *Dux*, their coding regions were amplified by PCR using primers with sequences at the ends of *Dux*, and mouse genome as the template. The amplified cDNAs were cloned and were sequenced. Although out of 30 clones, some of them were overlapped, 23 different *Dux* paralogs were identified, which were designated DuxG1 to DuxG23, in addition to the canonical *Dux* (Table 1 and Supplemental Fig. S1). These paralogs were only 1–12 bases different from *Dux*, and in all of them, no termination codon was recognized in the sequence, indicating that there are a large number of *Dux* family genes (more than 24 genes).

**Table 1:**
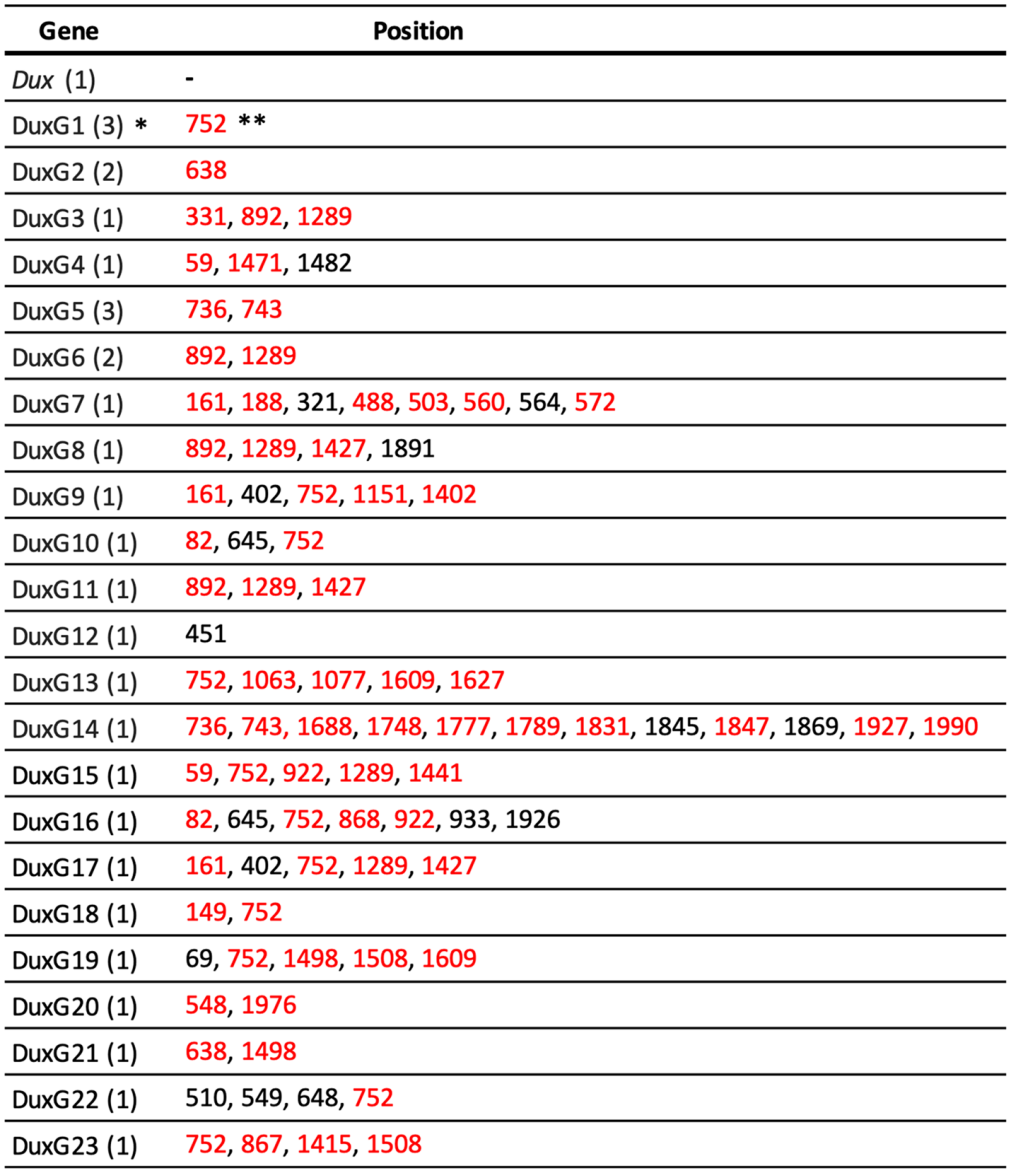
*Dux* family genes identified in the mouse genome.

*Parentheses indicate the number of clones obtained.

**Red letters indicate the position of non-synonymous replacements.

We examined whether these genes were expressed in early preimplantation embryos. To this end, PCR was performed using cDNA that had been reverse-transcribed using transcripts from early 2-cell stage embryos as the template. The primers used were capable of amplifying the whole coding regions (CDS) of *Dux* and all of the aforementioned *Dux* paralogs. However, no amplicon was obtained. Therefore, we decided to amplify the upstream region (31–889 bps) of the *Dux* family, which contains many mutations. After the PCR using the primers that matched all of them, the amplified cDNA was cloned and the resulting 37 clones were sequenced. As a result, 15 different *Dux* family sequences including *Dux* were identified. These sequences, excluding *Dux*, were designated DuxR1 to DuxR14 (Table 2 and Supplemental Fig. S2). In 37 clones, 8 clones were identical to *Dux*, and yet only comprised less than 1/4 of the 37 clones. Nine of the 14 sequences matched the aforementioned *Dux* paralog genes, suggesting that these *Dux* paralog genes did not come from the sequencing artifacts of the canonical *Dux*. In addition, none of these sequences had a stop codon in the middle of the sequence, which would lead to translation termination (Supplemental Fig. S3). When the nucleotide sequences of DuxR1 to R14 were converted into amino acid sequences, there were only a few amino acid differences from DUX (Table 2). These results suggest that many *Dux* family genes in tandem repeats are expressed in early 2-cell stage embryos and encode functional proteins.

**Table 2:**
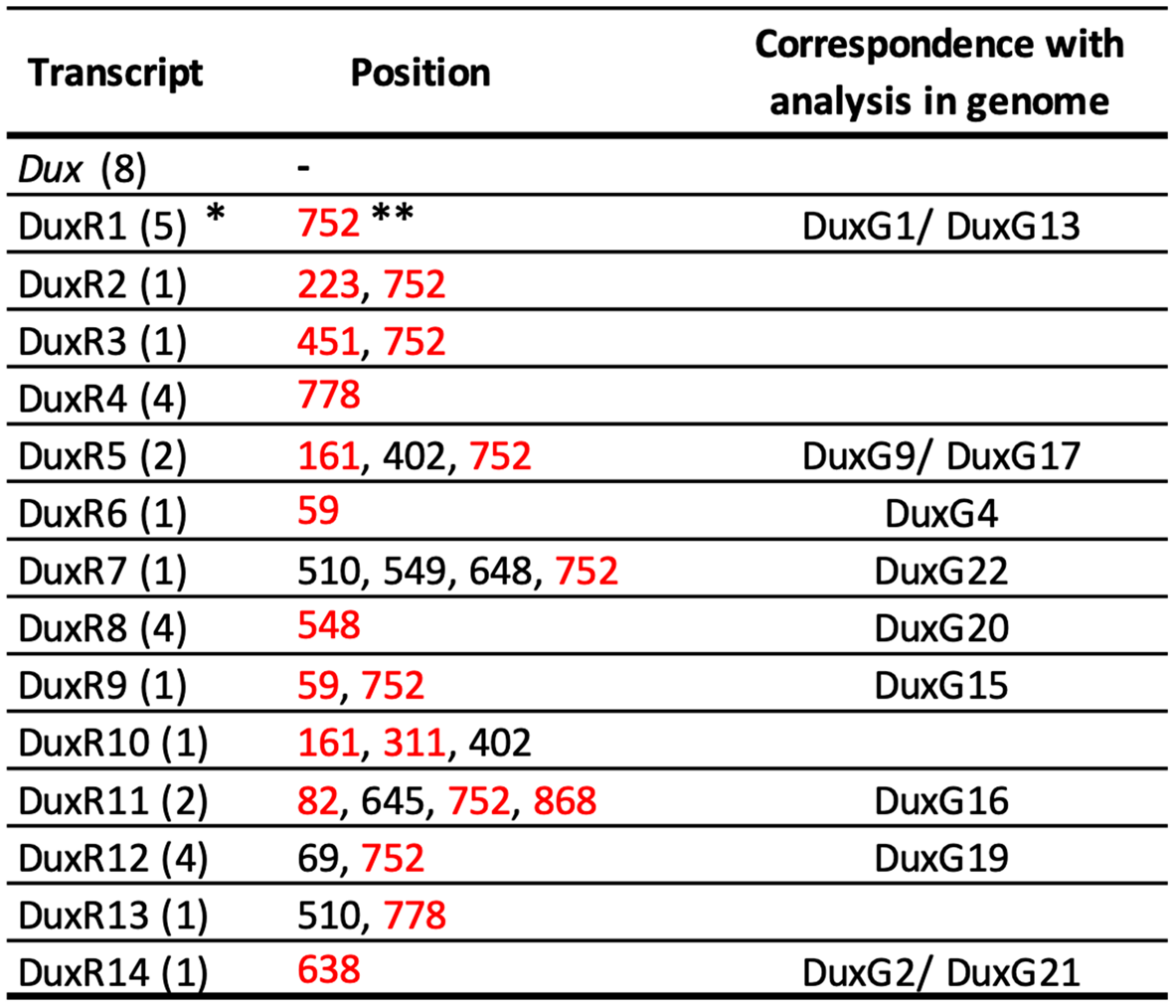
*Dux* family transcripts expressed in the early 2-cell stage embryos.

*Parentheses indicate the number of clones obtained.

**Red letters indicate non-synonymous replacements.

### Expression of *Dux* family genes during preimplantation development

**Figure 1:**
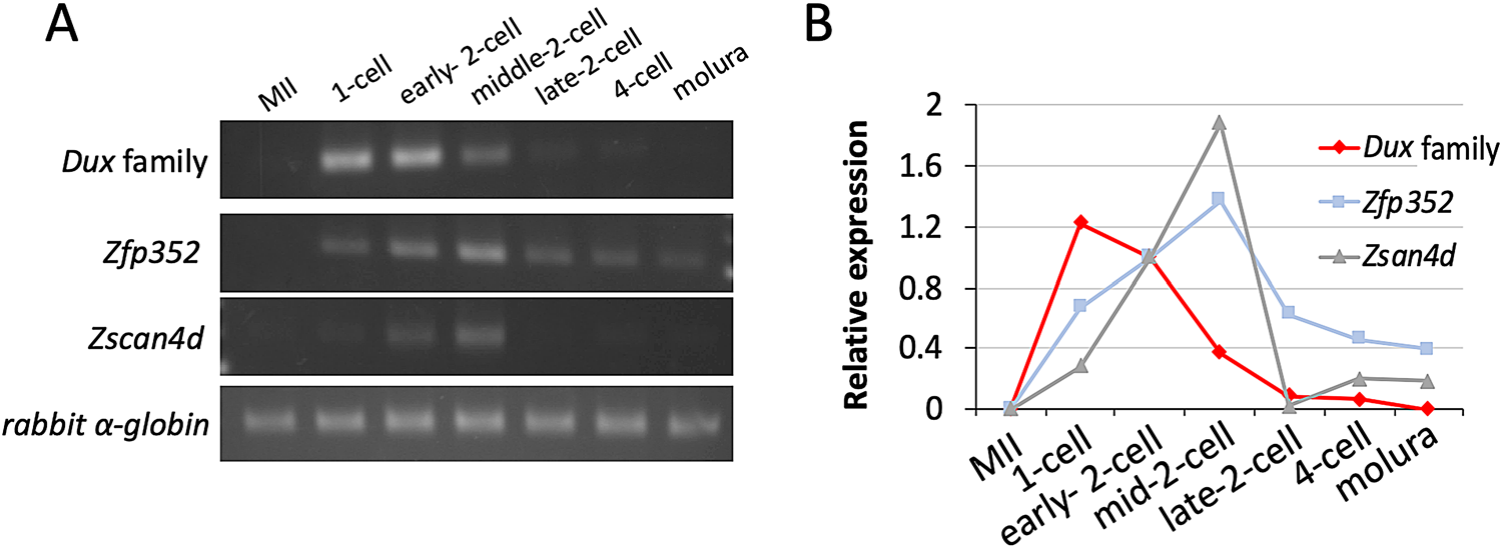
Expression of the *Dux* family, *Zfp352*, and *Zscan4d* during preimplantation development. Fifty MII stage oocytes and embryos at the 1-, 2-(early, mid and late), 4-cell, and morula stages were collected at 13, 16, 24, 32, 40 and 70 h post insemination (hpi), respectively, and subjected to reverse transcription polymerase chain reaction (RT-PCR). Two independent experiments were conducted and similar results were obtained. (**A**) Electrophoresis images of the *Dux* family, *Zfp352*, *Zscan4d*, and *rabbit* α-globin (external control) PCR products. (**B**) The band densities in (**A**) were quantified using ImageJ. The band densities of the three genes are relative to that of *rabbit α-globin*. The values at the early 2-cell stage were set to 1 and the relative values were calculated. The average value from two experiments is shown.

Changes in the expression levels of *Dux* family genes during preimplantation development were examined by RT-PCR, using the aforementioned primers that matched all of the *Dux* family genes expressed in the 2-cell stage embryos. Although the expression of *Dux* family genes was not detected in unfertilized eggs, it was detected in 1-cell stage embryos after fertilization. It was decreased during the 2-cell stage and hardly detected at the late 2-cell stage (Fig. 1A,B and Supplemental Fig. S4A). This indicates that the *Dux* family is a group of genes that are transiently expressed during the minor ZGA stage. We also examined the expression of two genes, *Zfp352* and *Zscan4d*, which are targets of *Dux*^8^. Their expression followed that of the *Dux* family genes. They were detected in 1-cell stage embryos, increased until the mid-2-cell stage, and then started decreasing (Fig. 1A,B and Supplemental Fig. S4A).

### Involvement of DNA replication in the expression of the *Dux* family at the 2-cell stage

**Figure 2:**
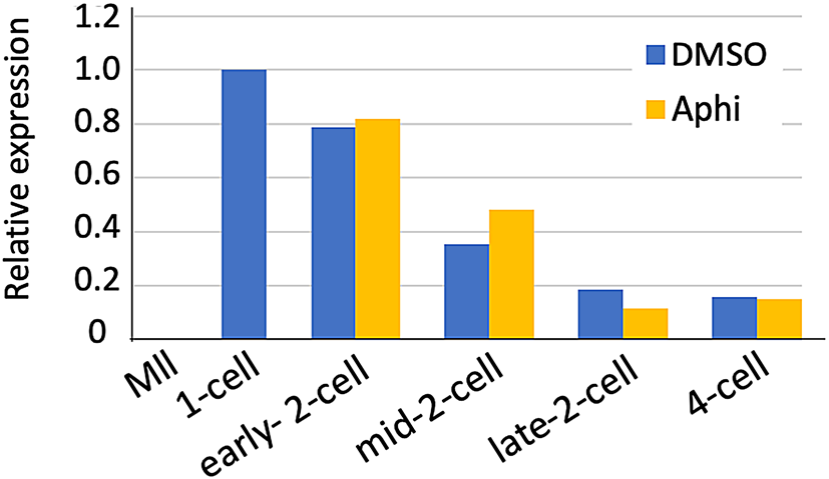
Effect of inhibition of DNA replication on the suppression of *Dux* family expression during the 2-cell stage. Fifty MII stage oocytes and embryos at the 1-, 2-(early, mid and late), 4-cell, and morula stages were collected at 13, 16, 24, 32 and 40 hpi, respectively. The expression levels of *Dux* family genes were examined by RT-PCR. *Rabbit α-globin* was used as an external control. The expressions of Dux family genes are relative to that of *rabbit α-globin*. Gene expression from the 1-cell stage was set to 1 and the relative values were calculated. Two independent experiments were conducted and similar results were obtained. The average value from two experiments is shown.

The expression levels of some minor ZGA genes decrease during the 2-cell stage and depend on DNA replication^21,22^. Therefore, DNA replication was inhibited using aphidicolin, an inhibitor of DNA polymerase, and the expression of the *Dux* family was examined at the late 2- to 4-cell stage to investigate whether the expression of the *Dux* family is suppressed by DNA replication. The results showed no significant differences compared to the control (Fig. 2). Therefore, the decrease in the expression of the *Dux* family during the 2-cell stage does not depend on DNA replication.

### Function of the *Dux* family

**Figure 3:**
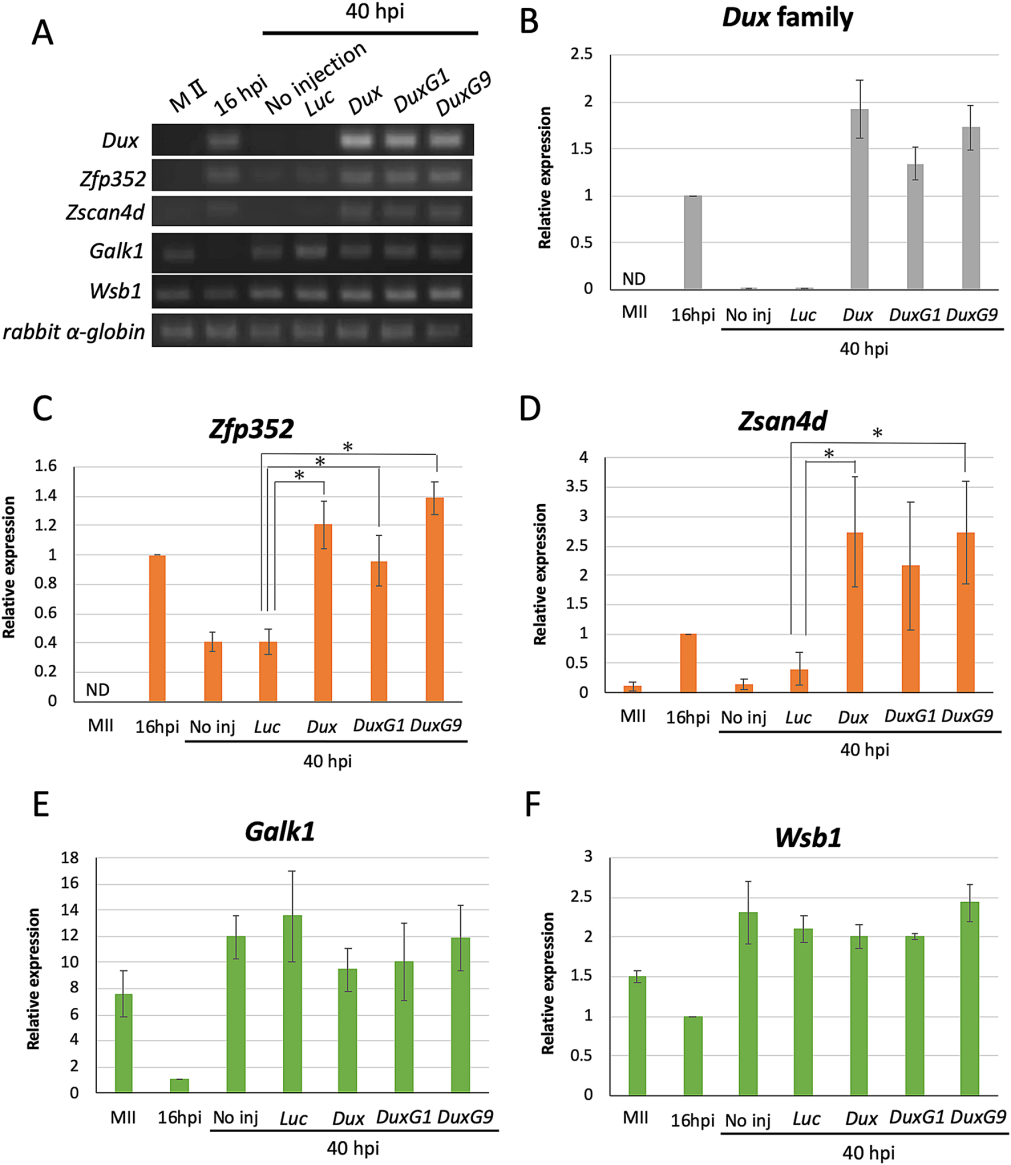
Effects of *Dux* family overexpression on the change in gene expression pattern in the early 2- and 4-cell stages. cRNA encoding *Dux*, *DuxG1*, *DuxG9*, or firefly luciferase (Luc, control) were microinjected into a blastomere of a 2-cell stage embryo at 16–20 hpi and cultured in vitro. Cells were collected and RT-PCR was conducted to amplify *Dux* family, *Zfp352*, *Zscan4d*, *Galk1*, *Wsb1* and *rabbit α-globin* (external control) at 40 hpi. MII stage oocytes and non injected embryos at 16 and 40 hpi were also collected for RT-PCR. Thirty cells were used for each sample. (**A**) Electrophoresis images of PCR products. (**B**–**F**) The band densities in (**A**) were quantified using image J. The band density of each gene is relative to that of *rabbit α-globin*. The values at the early 2-cell stage (16 hpi) were set to 1 and the relative values were calculated. Five (*Dux* family, *Zfp352* and *Zscan4d*) and three (*Galk1* and *Wsb1*) independent experiments were conducted. Error bars represent SE. Asterisks indicate significant difference (*p* < 0.05) by paired Student t-test.

**Figure 4:**
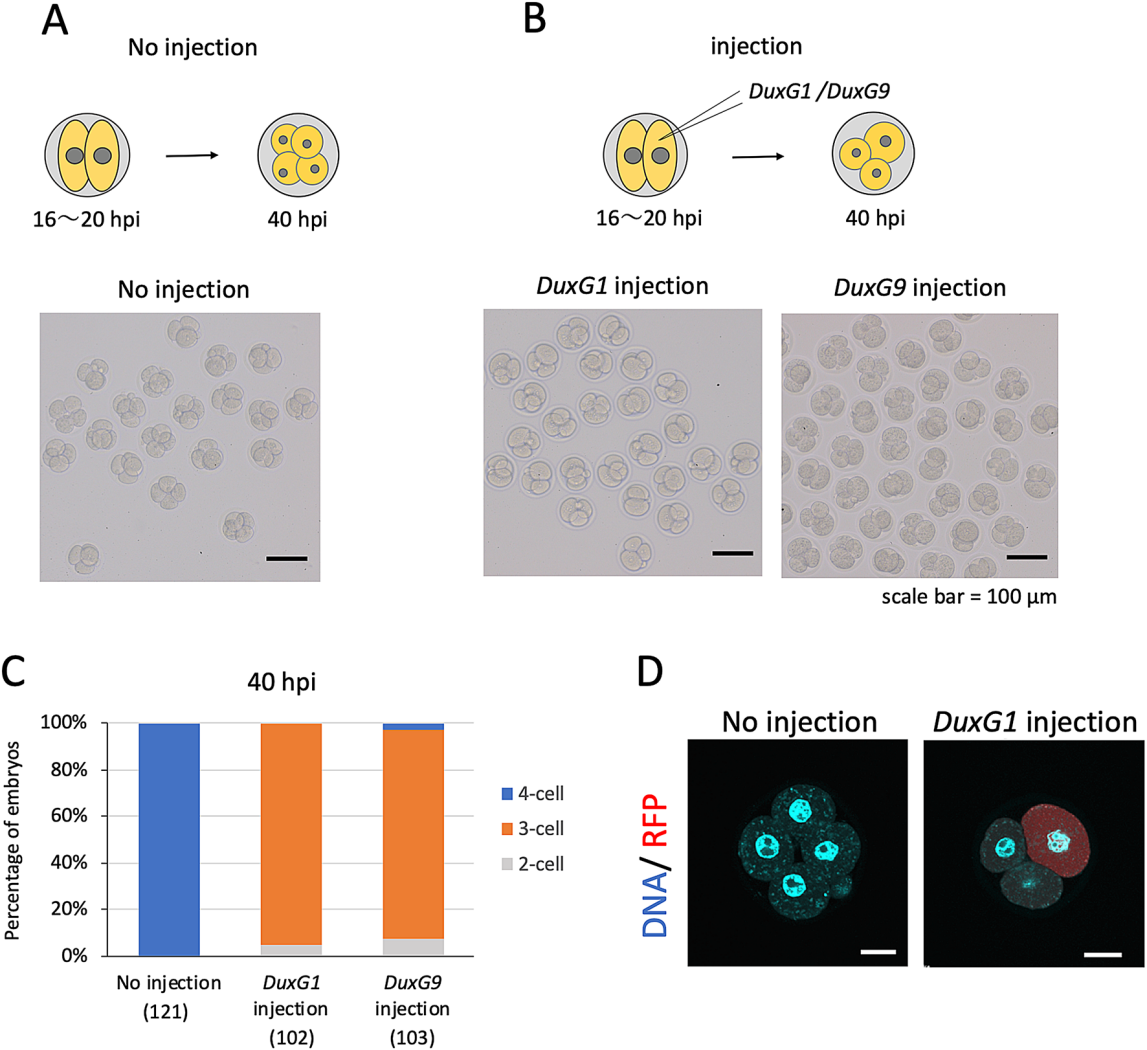
Effect of overexpression of the *Dux* family on the development of 2-cell stage embryos. A blastomere of a 2-cell stage embryo was microinjected with cRNA encoding *DuxG1* or *Dux G9* and then cultured until 40 hpi in vitro. Control embryos were not injected. (**A**,**B**) Schematic diagrams of the experiments. Photograph of embryos that were injected with (**A**) or without (**B**) *DuxG1* or *DuxG9* cRNA. Scale bar = 100 μm. (**C**) The developmental stages of the embryos at 40 hpi. Parentheses indicate the number of embryos observed at 40 hpi. (**D**) DuxG1 and RFP cRNA were simultaneously microinjected into a blastomere of 2-cell stage embryos and cultured until 45 hpi in vitro. Then the embryos were immunostained with RFP antibody at 45 hpi. DNA was detected by DAPI staining. Scale bar = 20 μm.

We overexpressed some of the *Dux* family genes to investigate their function in early pre-implantation embryos. We chose *DuxG1* and *DuxG9* because their expression was detected in 2-cell stage embryos, in addition to *Dux* (Table 2). DuxG1 has frequently been detected in experiments to identify the *Dux* family genes and *DuxG9* has the most mutations compared to *Dux* among the *Dux* family genes whose expressions had been observed (Table 2). The cRNA encoding *DuxG1*, *DuxG9*, or *Dux* was microinjected into one blastomere of 2-cell stage embryos between 16 and 20 h post insemination (hpi), because the expression of the *Dux* family was found to peak at 13 hpi and decrease at 16 hpi. Thereafter, the embryos were collected at 40 hpi and examined for the expression of the *Dux* family and the *Dux* target genes *Zfp352* and *Zscan4d*. The expressions of both of the *Dux* target genes were increased by the overexpression of *Du*x family genes (Fig. 3 and Supplemental Fig. S4B). It should be noted that the levels of the *Dux* target genes in the embryos overexpressing *DuxG1* or *DuxG9* were almost the same as those overexpressing *Dux*, suggesting that DUXG1 and DUXG9 have almost the same level of the activity as *Dux* to increase the expression of *Dux* target genes. The expressions of the *Dux*-independent genes *Wsb1* and *Galk1*^8^ showed no change after overexpression of *DuxG1*, *DuxG9*, or *Dux* (Fig. 3E,F). The embryos that were microinjected with *DuxG1* or *DuxG9* in a single blastomere developed to the 3-cell stage at 40 hpi, which is the time most of the control embryos developed to the 4-cell stage (Fig. 4). To confirm that the microinjected blastomere did not divide, red fluorescent protein (RFP) cRNA was microinjected together with *DuxG1* cRNA. The results showed that the microinjected blastomeres did not divide (Fig. 4D).

## Discussion

We identified 23 *Dux* family genes whose sequences were very similar to that of *Dux* in genomic DNA (*DuxG1*-*G23*; Table 1, Supplemental Fig. S1), and 14 mRNAs that were transcribed in 2-cell stage embryos (DuxR1-R14; Table 2, Supplemental Fig. S2). When the sequences of DuxR1 to R14 were compared to those of *DuxG1* to *G23* of the genome, there were nine matched pairs (Table 2). Therefore, five transcripts were derived from genes that had not yet been identified. Furthermore, because the sequences of the transcripts in nine of the aforementioned pairs were not full-length, the possibility cannot be excluded that they might have been derived from genes different from those described above. It is thus likely that there are many *Dux* family genes that have not yet been identified. Although it is unknown where these genes are located on the genome, in a DNA-FISH experiment using *Dux* as a probe, only one signal was found on chromosome 10^20^, suggesting that all (or most) of the *Dux* family genes are present in *Dux* tandem repeats.

**Figure 5:**
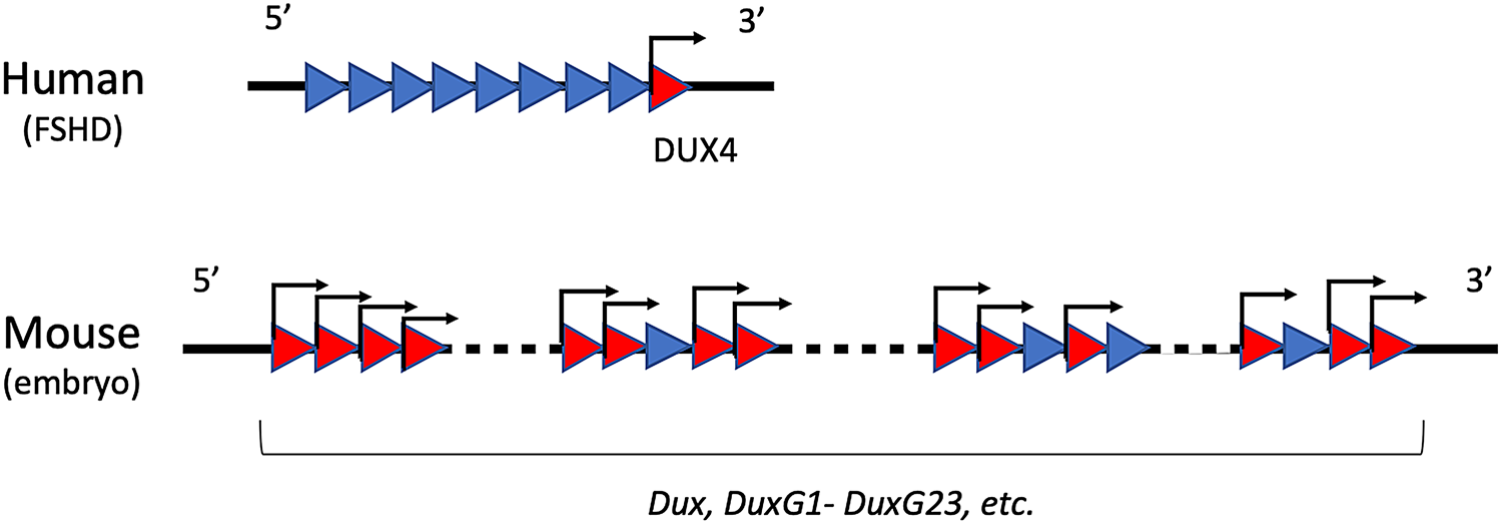
DUX4 expression in human FSHD myocytes and *Dux* family expression in mouse embryos. In human FSHD, only DUX4, which is located at the end of tandem repeats, is expressed in muscle, whereas in early mouse embryos, a large number of *Dux* paralogs are expressed during minor ZGA. The red and blue triangles represent active and inactive *Dux* family genes, respectively.

Erroneous expression of *DUX4* in human muscle causes FSHD^18,19^. In this disease, the number of tandem repeats is decreased from 11–100, which is normal, to less than 10, which leads to a change in chromatin structure, resulting in only the expression of *DUX4* that is located at the telomere end of the tandem repeats^23^. However, as described above, because there are at least 29 *Dux* family genes in mice, it is unlikely that expression occurs due to a small number of repetitions in early preimplantation embryos of mice. Furthermore, in FSHD, only one *DUX4* gene is expressed, whereas in mouse preimplantation embryos, at least 15 *Dux* family genes including *Dux* are expressed (Table 2; Fig. 5). Therefore, the mechanism by which *Dux* family genes is expressed from the 1-cell stage to the early 2-cell stage is likely to differ from that of muscle cells. The mechanism seems to be related to the chromatin structure specific to the embryos at these stages. The tandem repeats containing *DUX4* are present in the subtelomeric region in primates and African orders (such as those to which elephants and hyrax belong), and tandem repeats of DUXC, a homologue of DUX4, are present around the centromere in bovine and Laurasian orders (such as those to which dogs and dolphins belong)^24^. The areas around the subtelomere and centromere regions form heterochromatin and expression from these areas is suppressed. Although tandem repeats from mice containing the *Dux* family are located near the center of chromosome 10 and not near telomeres or centromeres^20^, a structure similar to that of subtelomeres has been observed there^20,24,25^. Therefore, it is likely that tandem repeats also form heterochromatin and are silenced in adult mouse cells. On the other hand, the chromatin structure is extremely loosened in 1-cell stage embryos and become tightened during the 2-cell stage^26^. Although in adult cells, pericentromeric regions form constitutive heterochromatin, which gathers to form chromocenters, they are not formed at the 1-cell and early 2-cell stages. This loosened chromatin structure causes promiscuous transcription from many regions all over the genome including pericentromeric regions^27–29^. Therefore, the tandem repeat containing the *Dux* family may have a loosened chromatin structure without forming heterochromatin at the 1-cell and early 2-cell stages, leading to the expression of the *Dux* family genes. In this case, unlike the expression of *DUX4* alone in human FSHD, the *Dux* family in the tandem repeats would be widely expressed in preimplantation embryos. Indeed, we found that several *Dux* family genes were expressed in 2-cell stage embryos (Table 2).

We have shown that the *Dux* family is a group of genes that are transiently expressed during minor ZGA (Fig. 1 and Supplemental Fig. S4A). DNA replication at the 2-cell stage is involved in the reduced expression of genes that are transiently expressed only at the time of minor ZGA^30^. When DNA replication is inhibited at the 2-cell stage, the expression of some of these genes is maintained even at the late 2-cell stage^3,21^. Therefore, we considered that the suppression of the *Dux* family genes at the late 2-cell stage might be regulated by DNA replication. However, inhibition of DNA replication by aphidicolin did not affect the expression of the *Dux* family (Fig. 2). Alternatively, the reduction of their expression at the 2-cell stage is related to the expression of the retrotransposon LINE1^31^. Therefore, it is possible that the expression of Dux is suppressed by LINE1 in the 2-cell stage, and heterochromatin is gradually formed during subsequent embryonic development, which becomes responsible for the suppression of the *Dux* family genes.

The C-terminus of mouse *Dux* and human *DUX4* is conserved^20,32^. The result of an experiment to determine the domain in human *DUX4* involved in the occurrence of FSHD revealed that the C terminus domain was important^33^. Furthermore, the overexpression of *DUX4* caused the expression of *Zscan4*, a ZGA gene, in HeLa cells, but *DUX4* lacking the C-terminus did not. We examined the sequences of C-terminal region (1981–2025 bps) in 23 *Dux* family genes obtained in this study, and found only one mutation in this region in a single gene, *DuxG14*. This suggests that most *Dux* family genes might be functional.

To investigate the function of the *Dux* family in preimplantation embryos, we overexpressed those genes. Their expressions increased the expression of some major ZGA genes in pre-implantation embryos (Fig. 3A and Supplemental Fig. S4B). In this experiment, we overexpressed *DuxG1*, *DuxG9*, and *Dux*, because their expressions were confirmed in 2-cell stage embryos. Because microinjection of the same amount of these three types increased the expression of *Dux* target genes to similar levels, this suggests that the *Dux* family genes may have similar functions in early preimplantation embryos.

In the present study, we found that there are a number of *Dux* family genes that are expressed during minor ZGA. These genes seem to function similarly to regulate the expression of some major ZGA genes. These characteristics seem to be suitable to ensure a sufficient amount of functional *Dux* and its paralogs. Because transcription is regulated independently of enhancers during minor ZGA, there seems to be no mechanism to enhance the expression of particular genes during this period^3,30,34^. In addition, transcriptional activity is low and the period (less than 10 h) is not long during minor ZGA^2^. Therefore, although a large number of genes are expressed during ZGA, the expression level of each gene is very low^3,4^. Under these conditions, an efficient way to produce a sufficient amount of transcripts would be to have multiple genes with similar function, as was found in the *Dux* family.

## Materials and methods

### Collection and culture of oocytes and embryos

MII stage oocytes were obtained from 3-week-old C57BL/6N (B6N) (Japan SLC, Inc., Shizuoka, Japan) or B6D2F1 (BDF1) (Japan SLC, Inc.) female mice, which were intraperitoneally administered with 5 IU of serotropin (ASKA Pharmaceutical, Co., Ltd., Tokyo, Japan) followed by 5 IU of human chorionic gonadotropin (hCG) (ASKA Pharmaceutical Co., Ltd.) for 48 h later to induce superovulation. The mice were sacrificed by cervical dislocation, and MII stage oocytes were obtained from the ampulla of oviduct in human tubal fluid (HTF) medium^35^. Spermatozoa were obtained from the cauda epididymis of adult B6N or ICR male mice (Japan SLC, Inc.), and cultured in HTF medium. In vitro fertilization was performed by adding sperm to HTF medium containing MII stage oocytes. Six hours after insemination, the fertilized oocytes were transferred to K^+^-modified simplex optimized medium (KSOM)^36^ to remove sperm and cumulus cells. Only fertilized oocytes containing two pronuclei were culled under a stereomicroscope, and then cultured in KSOM. The medium was covered with mineral oil and placed in an incubator at 38 °C containing 5% CO_2_. Embryos at various developmental stages were collected according to the following time schedule: 1-cell stage, 10 h post insemination (hpi); early 2-cell stage, 16 hpi; mid-2-cell stage, 24 hpi; late-2-cell stage, 32 hpi; 4-cell stage, 40 hpi; morula stage, 70 hpi.

All of the procedures using animals were reviewed and approved by the University of Tokyo Institutional Animal Care and Use Committee and were performed in accordance with the Guiding Principles for the Care and Use of Laboratory Animals.

### Inhibition of DNA replication

To inhibit DNA replication at the 2-cell stage, embryos were transferred to KSOM medium containing 3 µg/mL aphidicolin (Sigma-Aldrich, St. Louis, MO, USA), a DNA replication inhibitor, at 14 hpi at which time most of the embryos had just cleaved. As a control, embryos were cultured in KSOM medium containing 0.3% DMSO, which is the solvent that was used to resuspend aphidicolin.

### Reverse transcription polymerase chain reaction (RT-PCR)

Fifty (unless otherwise specified) oocytes or embryos were collected in 200 µL ISOGEN (Nippon Gene Co., Ltd., Tokyo, Japan). After adding rabbit α-globin as an external control, RNA extraction was performed. Genomic DNA was removed using RQ1 RNase-Free DNase (Promega Corporation, Madison, WI, USA), and RNA was extracted after adding 200 µL ISOGEN. Reverse transcription was performed using random hexamers from a PrimeScript RT-PCR kit (TaKaRa Bio Inc, Shiga, Japan). PCR was performed using Ex taq DNA polymerase (TaKaRa Bio Inc). The primers and conditions used for PCR are shown in Supplemental Table S1.

### Vector construction

To identify *Dux* paralogs in the mouse genome, constructs containing the coding regions (CRs; 2025 bps) of *Dux* paralogs were prepared. The CDSs of full-length *Dux* paralogs were amplified by PCR using the genome of C57BL6N mouse as a template, DNA polymerase with proofreading activity (KOD-Plus-; TOYOBO Co., Ltd., Osaka, Japan), and the following primers.

- Forward: 5′-ATGAATTCGCCACCATGGCAGAAGCTGGCAGC-3′.
- Reverse: 5′-ATGAATTCTCAGAGCATATCTAGAAGAGTCTGATATTCTT-3′.

These primers contained a Kozak sequence and a restriction enzyme site (EcoR1) to allow for the constructs to also be used for overexpression experiments. After amplification, an additional PCR was performed using Ex taq (Takara Bio Inc) to add a protruding end of adenosine at 95 °C for 2 min and at 72 °C for 15 min. The amplified DNA was electrophoresed on an agarose gel, and the PCR fragment was purified according to the procedure in the Wizard SV Gel and PCR Clean-Up System (Promega Corporation). The purified PCR fragment was cloned using a TOPO TA cloning kit (pCRII TOPO vector; Life Technologies, Carlsbad, CA, USA) according to the manufacturer’s instructions, and introduced into DH5α (TaKaRa, Cell density: 1–2 × 10^9^ bacteria/mL). Thereafter, the plasmid was extracted using a Plasmid DNA Extraction Mini Kit (Chiyoda Science Co., Ltd., Tokyo, Japan). The extracted plasmid was subjected to DNA sequencing using a 3500 Genetics Analyzer (Thermo Fisher Scientific Inc., Waltham, MA, USA). Because the full length *Dux* CDS is too long to be completely sequenced using a single set of primers (2025 bps), four sets of forward and reverse primers were used (Supplemental Table S2).

To identify the transcripts expressed in 2-cell stage embryos, cDNA was prepared as described above in the RT-PCR section, and part of the *Dux* CDS (31–889 bps) was amplified using the following primers: forward, 5′-AGTGGTGTGGCACGGGAA-3′; reverse, 5′-AGCTCTCCTGGGAACCTTCA-3′. The PCR products were inserted into a pCRII TOPO vector and used for sequencing as described above.

To overexpress *Dux*, artificial gene synthesis of *Dux* was outsourced to Thermo Fisher Scientific, because the Dux sequence could not be obtained by cloning using the genome as a template. Using the plasmid containing *Dux* as a template, full-length *Dux* CDS was amplified by PCR and the products were inserted into a pCRII TOPO vector and used for sequencing.

### In vitro transcription

In vitro transcription (IVT) was performed to prepare cRNAs encoding *Dux*, *DuxG1*, *DuxG9*, and firefly luciferase (*Luc*), which had been prepared previously. The expression vectors containing *Dux*, *DuxG9*, and *Luc* were treated with the restriction enzyme EcoRV (FastDigest, Thermo Fisher Scientific Inc.). The expression vector containing *DuxG1* was treated with SpeI (FastDigest, Thermo Fisher Scientific Inc.). They were purified by phenol chloroform/ethanol precipitation. Thereafter, IVT was performed using a T7 or Sp6 mMESSAGE mMACHINE (Thermo Fisher Scientific Inc.), according to the manufacturer’s instructions. After adding a poly (A) tail to the transcribed cRNA using a Poly (A) tailing kit (Thermo Fisher Scientific Inc.), the cRNA was purified using a lithium chloride precipitation solution and dissolved in nuclease-free water.

### Microinjection

cRNAs encoding *Dux* and *Dux* paralogs were adjusted to a concentration of 500 ng/µL, and ~ 10 pL cRNA and were microinjected into a blastomere of 2-cell stage embryos between 16 and 20 hpi in KSOM-HEPES medium covered with mineral oil. To discriminate the blastomere that had been microinjected, 500 ng/µL cRNA encoding red fluorescent protein (RFP) was microinjected together with the *Dux* paralog-encoding cRNA.

### Immunocytochemistry

The embryos were fixed with 3.7% paraformaldehyde (PFA) in PBS at room temperature for 20 min, and then washed with PBS containing 0.1 mg/mL bovine serum albumin (BSA; Sigma-Aldrich) (0.1% BSA in PBS) three times. The membrane was permeabilized with 0.5% Triton X-100 (Sigma-Aldrich) in PBS (0.5% Triton X-100 in PBS) at room temperature for 15 min. After being washed three times with 0.1% BSA in PBS, the embryos were treated with the primary antibody against RFP (Abcam, Cambridge, UK; diluted 500-fold with 0.1% BSA in PBS) overnight at 4 °C followed by treatment with the secondary antibody (AlexaFluor 568 donkey-anti rabbit IgG; Life Technologies; diluted 200-fold with 0.1% BSA in PBS) at room temperature for 1 h. After being washed with 0.1% BSA in PBS three times, the samples were mounted in VECTASHIELD Mounting Medium (Vector Laboratories, Burlingame, CA, USA) containing 3 µg/mL 4,6-diamidino-2-phenylindole (DAPI: Dojindo Laboratories, Kumamoto, Japan) on a glass slide. The fluorescence was observed using an FV3000 confocal laser-scanning microscope (Olympus, Tokyo, Japan).

## Supplementary information

Supplementary Information.

## References

[1] Moore GP, Lintern-Moore S, Peters H, Faber M (1974). RNA synthesis in the mouse oocyte. J. Cell Biol..

[2] Aoki F, Worrad DM, Schultz RM (1997). Regulation of transcriptional activity during the first and second cell cycles in the preimplantation mouse embryo. Dev. Biol..

[3] Abe K (2015). The first murine zygotic transcription is promiscuous and uncoupled from splicing and 3′ processing. EMBO J..

[4] Yamamoto R, Aoki F (2017). A unique mechanism regulating gene expression in 1-cell embryos. J. Reprod. Dev..

[5] Abe KI (2018). Minor zygotic gene activation is essential for mouse preimplantation development. Proc. Natl. Acad. Sci. U.S.A..

[6] De Iaco A (2017). DUX-family transcription factors regulate zygotic genome activation in placental mammals. Nat. Genet..

[7] Hendrickson PG (2017). Conserved roles of mouse DUX and human DUX4 in activating cleavage-stage genes and MERVL/HERVL retrotransposons. Nat. Genet..

[8] Whiddon JL, Langford AT, Wong CJ, Zhong JW, Tapscott SJ (2017). Conservation and innovation in the DUX4-family gene network. Nat. Genet..

[9] Guo M (2019). Precise temporal regulation of Dux is important for embryo development. Cell Res..

[10] Chen Z, Zhang Y (2019). Loss of DUX causes minor defects in zygotic genome activation and is compatible with mouse development. Nat. Genet..

[11] De Iaco A, Verp S, Offner S, Grun D, Trono D (2020). DUX is a non-essential synchronizer of zygotic genome activation. Development.

[12] Mitsuhashi S (2017). Nanopore-based single molecule sequencing of the D4Z4 array responsible for facioscapulohumeral muscular dystrophy. Sci. Rep..

[13] Hewitt JE (1994). Analysis of the tandem repeat locus D4Z4 associated with facioscapulohumeral muscular dystrophy. Hum. Mol. Genet..

[14] Winokur ST (1994). The DNA rearrangement associated with facioscapulohumeral muscular dystrophy involves a heterochromatin-associated repetitive element: implications for a role of chromatin structure in the pathogenesis of the disease. Chromosome Res. Int. J. Mol. Supramol. Evol. Asp. Chromosome Biol..

[15] Gabriels J (1999). Nucleotide sequence of the partially deleted D4Z4 locus in a patient with FSHD identifies a putative gene within each 3.3 kb element. Gene.

[16] Dixit M (2007). DUX4, a candidate gene of facioscapulohumeral muscular dystrophy, encodes a transcriptional activator of PITX1. Proc. Natl. Acad. Sci. U. S. A..

[17] Snider L (2009). RNA transcripts, miRNA-sized fragments and proteins produced from D4Z4 units: new candidates for the pathophysiology of facioscapulohumeral dystrophy. Hum. Mol. Genet..

[18] Geng LN (2012). DUX4 activates germline genes, retroelements, and immune mediators: implications for facioscapulohumeral dystrophy. Dev. Cell.

[19] Hewitt JE (2015). Loss of epigenetic silencing of the DUX4 transcription factor gene in facioscapulohumeral muscular dystrophy. Hum. Mol. Genet..

[20] Clapp J (2007). Evolutionary conservation of a coding function for D4Z4, the tandem DNA repeat mutated in facioscapulohumeral muscular dystrophy. Am. J. Hum. Genet..

[21] Davis W, De Sousa PA, Schultz RM (1996). Transient expression of translation initiation factor eIF-4C during the 2-cell stage of the preimplantation mouse embryo: identification by mRNA differential display and the role of DNA replication in zygotic gene activation. Dev. Biol..

[22] Sonehara H, Nagata M, Aoki F (2008). Roles of the first and second round of DNA replication in the regulation of zygotic gene activation in mice. J. Reprod. Dev..

[23] Lemmers RJ (2010). A unifying genetic model for facioscapulohumeral muscular dystrophy. Science.

[24] Leidenroth A (2012). Evolution of DUX gene macrosatellites in placental mammals. Chromosoma.

[25] Flint J (1997). Sequence comparison of human and yeast telomeres identifies structurally distinct subtelomeric domains. Hum. Mol. Genet..

[26] Ooga M, Fulka H, Hashimoto S, Suzuki MG, Aoki F (2016). Analysis of chromatin structure in mouse preimplantation embryos by fluorescent recovery after photobleaching. Epigenetics.

[27] Ahmed K (2010). Global chromatin architecture reflects pluripotency and lineage commitment in the early mouse embryo. PLoS ONE.

[28] Akiyama T, Suzuki O, Matsuda J, Aoki F (2011). Dynamic replacement of histone H3 variants reprograms epigenetic marks in early mouse embryos. PLoS Genet..

[29] Probst AV (2010). A strand-specific burst in transcription of pericentric satellites is required for chromocenter formation and early mouse development. Dev. Cell.

[30] Schultz RM (1993). Regulation of zygotic gene activation in the mouse. BioEssays News Rev. Mol. Cell. Dev. Biol..

[31] Percharde M (2018). A LINE1-nucleolin partnership regulates early development and ESC identity. Cell.

[32] Eidahl JO (2016). Mouse Dux is myotoxic and shares partial functional homology with its human paralog DUX4. Hum. Mol. Genet..

[33] Mitsuhashi H (2018). Functional domains of the FSHD-associated DUX4 protein. Biol. Open.

[34] Nothias JY, Majumder S, Kaneko KJ, DePamphilis ML (1995). Regulation of gene expression at the beginning of mammalian development. J. Biol. Chem..

[35] Quinn P, Begley AJ (1984). Effect of human seminal plasma and mouse accessory gland extracts on mouse fertilization in vitro. Aust. J. Biol. Sci..

[36] Lawitts JA, Biggers JD (1993). Culture of preimplantation embryos. Methods Enzymol..

