# Supplementary material for: Expression of Dux family genes in early preimplantation embryos
Source: Sci Rep. 2020 Nov 10;10:19396. doi: 10.1038/s41598-020-76538-9 (PMC7655946; doi:10.1038/s41598-020-76538-9)
Supplement: Supplementary file 1 — Supplementary Information. [file 41598_2020_76538_MOESM1_ESM.pdf]

Expression of *Dux* family genes in early preimplantation embryos

Kenta Sugie[1], Satoshi Funaya[1], Machika Kawamura[1], Toshinobu Nakamura[2], Masataka G. Suzuki[1], and Fugaku Aoki[1]*

[1]*Department of Integrated Biosciences, Graduate School of Frontier Sciences, The University of Tokyo, Kashiwa, 277-8562 Japan.*

[2]*Department of Bio-Science, Nagahama Institute of Bio-Science and Technology, Shiga 526-0829, Japan.*

**Address correspondence to: Fugaku Aoki, Department of Integrated Biosciences, Graduate School of Frontier Sciences, The University of Tokyo, Seimei-Building 302,*
5-1-5 Kashiwanoha, Kashiwa 277-8652, Japan, E-mail: aokif@edu.k.u-tokyo.ac.jp

Author contributions:

FA conceived and supervised the study; KS and FA designed the experiments; KS performed the experiments; KS, SF, MK, MGS, and FA analyzed the data; and KS and FA wrote the manuscript.

Supplemental Table. S1. Primers and PCR conditions used for RT-PCR

Target gene	Primer		Annealing temperature	Cycle
Dux family	Forward	5'-GCCCTGCTATCAACTTTCAAGAAG-3'	57 ℃	36
	Reverse	5'-AGGCTTGCCCTAGGATCCTGAG-3'		
Zfp352	Forward	5'-AAAGCCTTGATCCTCAGGTG-3'	57 ℃	36
	Reverse	5'-GCCGAAGAGTTTTTCTGAGG-3'		
Zscan4d	Forward	5'-AAGAGGTGAGGTGGAGGAGT-3'	57 ℃	37
	Reverse	5'-AAGGTCTTTTGCTGGTGCCT-3'		
Wsb1	Forward	5'-CTCCAAGGCAAGTCCCTAGC-3'	57 ℃	36
	Reverse	5'-TGTAAAGGGCGCTCTGTCTG-3'		
Galk1	Forward	5'-GGCAGGGAGCTAATGAGCAA-3'	57 ℃	36
	Reverse	5'-GGGCAGCTGACCTCATAGTC-3'		
rabbit α-globin	Forward	5'-GTGGGACAGGAGCTTGAAAT-3'	58 ℃	26
	Reverse	5'-GCAGCCACGGTGGCGAGTAT-3'		

Supplemental Table. S2. Primers used for DNA sequencing

Target	Primers	
Dux full length CDS (2025 bps)	Forwarad	
	M13F (vector origin)	5'-GTAAAACGACGGCCAG-3'
	Dux origin	5'-TCCAGGCCACAGGGTAGAG-3'
		5'-TGGAGACCCTGGTGGCA-3'
		5'-TCCCAAGTGGCACAGCG-3'
	Reverse	
	M13R (vector origin)	5'-CAGGAAACAGCTATGAC-3'
	Dux origin	5'-CTCTACCCTGTGGCCTGGA-3'
		5'-TGCCACCAGGGTCTCCA-3'
		5'-GGGAAGGCTTGGACTCTCTGA-3'
Part of *Dux* CDS (31-889 bps)	Forwarad	
	M13F (vector origin)	5'-GTAAAACGACGGCCAG-3'
	Reverse	
	M13R (vector origin)	5'-CAGGAAACAGCTATGAC-3'

Supplemental Fig. S1


```
Dux       ATGGCAGAAGCTGGCAGCCCTGTTGGTGGCAGTGGTGTGGCACGGGAATCCCGGCGGCGC  60
DuxG1     ATGGCAGAAGCTGGCAGCCCTGTTGGTGGCAGTGGTGTGGCACGGGAATCCCGGCGGCGC  60
DuxG2     ATGGCAGAAGCTGGCAGCCCTGTTGGTGGCAGTGGTGTGGCACGGGAATCCCGGCGGCGC  60
DuxG3     ATGGCAGAAGCTGGCAGCCCTGTTGGTGGCAGTGGTGTGGCACGGGAATCCCGGCGGCGC  60
DuxG4     ATGGCAGAAGCTGGCAGCCCTGTTGGTGGCAGTGGTGTGGCACGGGAATCCCGGCGGCAC  60
DuxG5     ATGGCAGAAGCTGGCAGCCCTGTTGGTGGCAGTGGTGTGGCACGGGAATCCCGGCGGCGC  60
DuxG6     ATGGCAGAAGCTGGCAGCCCTGTTGGTGGCAGTGGTGTGGCACGGGAATCCCGGCGGCGC  60
DuxG7     ATGGCAGAAGCTGGCAGCCCTGTTGGTGGCAGTGGTGTGGCACGGGAATCCCGGCGGCGC  60
DuxG8     ATGGCAGAAGCTGGCAGCCCTGTTGGTGGCAGTGGTGTGGCACGGGAATCCCGGCGGCGC  60
DuxG9     ATGGCAGAAGCTGGCAGCCCTGTTGGTGGCAGTGGTGTGGCACGGGAATCCCGGCGGCGC  60
DuxG10    ATGGCAGAAGCTGGCAGCCCTGTTGGTGGCAGTGGTGTGGCACGGGAATCCCGGCGGCGC  60
DuxG11    ATGGCAGAAGCTGGCAGCCCTGTTGGTGGCAGTGGTGTGGCACGGGAATCCCGGCGGCGC  60
DuxG12    ATGGCAGAAGCTGGCAGCCCTGTTGGTGGCAGTGGTGTGGCACGGGAATCCCGGCGGCGC  60
DuxG13    ATGGCAGAAGCTGGCAGCCCTGTTGGTGGCAGTGGTGTGGCACGGGAATCCCGGCGGCGC  60
DuxG14    ATGGCAGAAGCTGGCAGCCCTGTTGGTGGCAGTGGTGTGGCACGGGAATCCCGGCGGCGC  60
DuxG15    ATGGCAGAAGCTGGCAGCCCTGTTGGTGGCAGTGGTGTGGCACGGGAATCCCGGCGGCAC  60
DuxG16    ATGGCAGAAGCTGGCAGCCCTGTTGGTGGCAGTGGTGTGGCACGGGAATCCCGGCGGCGC  60
DuxG17    ATGGCAGAAGCTGGCAGCCCTGTTGGTGGCAGTGGTGTGGCACGGGAATCCCGGCGGCGC  60
DuxG18    ATGGCAGAAGCTGGCAGCCCTGTTGGTGGCAGTGGTGTGGCACGGGAATCCCGGCGGCGC  60
DuxG19    ATGGCAGAAGCTGGCAGCCCTGTTGGTGGCAGTGGTGTGGCACGGGAATCCCGGCGGCGC  60
DuxG20    ATGGCAGAAGCTGGCAGCCCTGTTGGTGGCAGTGGTGTGGCACGGGAATCCCGGCGGCGC  60
DuxG21    ATGGCAGAAGCTGGCAGCCCTGTTGGTGGCAGTGGTGTGGCACGGGAATCCCGGCGGCGC  60
DuxG22    ATGGCAGAAGCTGGCAGCCCTGTTGGTGGCAGTGGTGTGGCACGGGAATCCCGGCGGCGC  60
DuxG23    ATGGCAGAAGCTGGCAGCCCTGTTGGTGGCAGTGGTGTGGCACGGGAATCCCGGCGGCGC  60
                                                                    *

Dux       AGGAAGACGGTTTGGCAGGCCTGGCAAGAGCAGGCCCTGCTATCAACTTTCAAGAAGAAG 120
DuxG1     AGGAAGACGGTTTGGCAGGCCTGGCAAGAGCAGGCCCTGCTATCAACTTTCAAGAAGAAG 120
DuxG2     AGGAAGACGGTTTGGCAGGCCTGGCAAGAGCAGGCCCTGCTATCAACTTTCAAGAAGAAG 120
DuxG3     AGGAAGACGGTTTGGCAGGCCTGGCAAGAGCAGGCCCTGCTATCAACTTTCAAGAAGAAG 120
DuxG4     AGGAAGACGGTTTGGCAGGCCTGGCAAGAGCAGGCCCTGCTATCAACTTTCAAGAAGAAG 120
DuxG5     AGGAAGACGGTTTGGCAGGCCTGGCAAGAGCAGGCCCTGCTATCAACTTTCAAGAAGAAG 120
DuxG6     AGGAAGACGGTTTGGCAGGCCTGGCAAGAGCAGGCCCTGCTATCAACTTTCAAGAAGAAG 120
DuxG7     AGGAAGACGGTTTGGCAGGCCTGGCAAGAGCAGGCCCTGCTATCAACTTTCAAGAAGAAG 120
DuxG8     AGGAAGACGGTTTGGCAGGCCTGGCAAGAGCAGGCCCTGCTATCAACTTTCAAGAAGAAG 120
DuxG9     AGGAAGACGGTTTGGCAGGCCTGGCAAGAGCAGGCCCTGCTATCAACTTTCAAGAAGAAG 120
DuxG10    AGGAAGACGGTTTGGCAGGCCGGGCAAGAGCAGGCCCTGCTATCAACTTTCAAGAAGAAG 120
DuxG11    AGGAAGACGGTTTGGCAGGCCTGGCAAGAGCAGGCCCTGCTATCAACTTTCAAGAAGAAG 120
DuxG12    AGGAAGACGGTTTGGCAGGCCTGGCAAGAGCAGGCCCTGCTATCAACTTTCAAGAAGAAG 120
DuxG13    AGGAAGACGGTTTGGCAGGCCTGGCAAGAGCAGGCCCTGCTATCAACTTTCAAGAAGAAG 120
DuxG14    AGGAAGACGGTTTGGCAGGCCTGGCAAGAGCAGGCCCTGCTATCAACTTTCAAGAAGAAG 120
DuxG15    AGGAAGACGGTTTGGCAGGCCTGGCAAGAGCAGGCCCTGCTATCAACTTTCAAGAAGAAG 120
DuxG16    AGGAAGACGGTTTGGCAGGCCGGGCAAGAGCAGGCCCTGCTATCAACTTTCAAGAAGAAG 120
DuxG17    AGGAAGACGGTTTGGCAGGCCTGGCAAGAGCAGGCCCTGCTATCAACTTTCAAGAAGAAG 120
DuxG18    AGGAAGACGGTTTGGCAGGCCTGGCAAGAGCAGGCCCTGCTATCAACTTTCAAGAAGAAG 120
DuxG19    AGGAAGACAGTTTGGCAGGCCTGGCAAGAGCAGGCCCTGCTATCAACTTTCAAGAAGAAG 120
DuxG20    AGGAAGACGGTTTGGCAGGCCTGGCAAGAGCAGGCCCTGCTATCAACTTTCAAGAAGAAG 120
DuxG21    AGGAAGACGGTTTGGCAGGCCTGGCAAGAGCAGGCCCTGCTATCAACTTTCAAGAAGAAG 120
DuxG22    AGGAAGACGGTTTGGCAGGCCTGGCAAGAGCAGGCCCTGCTATCAACTTTCAAGAAGAAG 120
DuxG23    AGGAAGACGGTTTGGCAGGCCTGGCAAGAGCAGGCCCTGCTATCAACTTTCAAGAAGAAG 120
                  *            *

Dux       AGATACCTGAGCTTCAAGGAGAGGAAGGAGCTGGCCAAGCGAATGGGGGTCTCAGATTGC 180
DuxG1     AGATACCTGAGCTTCAAGGAGAGGAAGGAGCTGGCCAAGCGAATGGGGGTCTCAGATTGC 180
DuxG2     AGATACCTGAGCTTCAAGGAGAGGAAGGAGCTGGCCAAGCGAATGGGGGTCTCAGATTGC 180
DuxG3     AGATACCTGAGCTTCAAGGAGAGGAAGGAGCTGGCCAAGCGAATGGGGGTCTCAGATTGC 180
DuxG4     AGATACCTGAGCTTCAAGGAGAGGAAGGAGCTGGCCAAGCGAATGGGGGTCTCAGATTGC 180
DuxG5     AGATACCTGAGCTTCAAGGAGAGGAAGGAGCTGGCCAAGCGAATGGGGGTCTCAGATTGC 180
DuxG6     AGATACCTGAGCTTCAAGGAGAGGAAGGAGCTGGCCAAGCGAATGGGGGTCTCAGATTGC 180
DuxG7     AGATACCTGAGCTTCAAGGAGAGGAAGGAGCTGGCCAAGCAAATGGGGGTCTCAGATTGC 180
DuxG8     AGATACCTGAGCTTCAAGGAGAGGAAGGAGCTGGCCAAGCGAATGGGGGTCTCAGATTGC 180
DuxG9     AGATACCTGAGCTTCAAGGAGAGGAAGGAGCTGGCCAAGCAAATGGGGGTCTCAGATTGC 180
DuxG10    AGATACCTGAGCTTCAAGGAGAGGAAGGAGCTGGCCAAGCGAATGGGGGTCTCAGATTGC 180
DuxG11    AGATACCTGAGCTTCAAGGAGAGGAAGGAGCTGGCCAAGCGAATGGGGGTCTCAGATTGC 180
DuxG12    AGATACCTGAGCTTCAAGGAGAGGAAGGAGCTGGCCAAGCGAATGGGGGTCTCAGATTGC 180
DuxG13    AGATACCTGAGCTTCAAGGAGAGGAAGGAGCTGGCCAAGCGAATGGGGGTCTCAGATTGC 180
DuxG14    AGATACCTGAGCTTCAAGGAGAGGAAGGAGCTGGCCAAGCGAATGGGGGTCTCAGATTGC 180
DuxG15    AGATACCTGAGCTTCAAGGAGAGGAAGGAGCTGGCCAAGCGAATGGGGGTCTCAGATTGC 180
DuxG16    AGATACCTGAGCTTCAAGGAGAGGAAGGAGCTGGCCAAGCGAATGGGGGTCTCAGATTGC 180
DuxG17    AGATACCTGAGCTTCAAGGAGAGGAAGGAGCTGGCCAAGCAAATGGGGGTCTCAGATTGC 180
DuxG18    AGATACCTGAGCTTCAAGGAGAGGAAGGGGCTGGCCAAGCGAATGGGGGTCTCAGATTGC 180
DuxG19    AGATACCTGAGCTTCAAGGAGAGGAAGGAGCTGGCCAAGCGAATGGGGGTCTCAGATTGC 180
DuxG20    AGATACCTGAGCTTCAAGGAGAGGAAGGAGCTGGCCAAGCGAATGGGGGTCTCAGATTGC 180
DuxG21    AGATACCTGAGCTTCAAGGAGAGGAAGGAGCTGGCCAAGCGAATGGGGGTCTCAGATTGC 180
DuxG22    AGATACCTGAGCTTCAAGGAGAGGAAGGAGCTGGCCAAGCGAATGGGGGTCTCAGATTGC 180
DuxG23    AGATACCTGAGCTTCAAGGAGAGGAAGGAGCTGGCCAAGCGAATGGGGGTCTCAGATTGC 180
                                      *           *
```

```
Dux       CGCATCCGCGTGTGGTTTCAGAACCGCAGGAATCGCAGTGGAGAGGAGGGGCATGCCTCA 240
DuxG1     CGCATCCGCGTGTGGTTTCAGAACCGCAGGAATCGCAGTGGAGAGGAGGGGCATGCCTCA 240
DuxG2     CGCATCCGCGTGTGGTTTCAGAACCGCAGGAATCGCAGTGGAGAGGAGGGGCATGCCTCA 240
DuxG3     CGCATCCGCGTGTGGTTTCAGAACCGCAGGAATCGCAGTGGAGAGGAGGGGCATGCCTCA 240
DuxG4     CGCATCCGCGTGTGGTTTCAGAACCGCAGGAATCGCAGTGGAGAGGAGGGGCATGCCTCA 240
DuxG5     CGCATCCGCGTGTGGTTTCAGAACCGCAGGAATCGCAGTGGAGAGGAGGGGCATGCCTCA 240
DuxG6     CGCATCCGCGTGTGGTTTCAGAACCGCAGGAATCGCAGTGGAGAGGAGGGGCATGCCTCA 240
DuxG7     CGCATCCACGTGTGGTTTCAGAACCGCAGGAATCGCAGTGGAGAGGAGGGGCATGCCTCA 240
DuxG8     CGCATCCGCGTGTGGTTTCAGAACCGCAGGAATCGCAGTGGAGAGGAGGGGCATGCCTCA 240
DuxG9     CGCATCCGCGTGTGGTTTCAGAACCGCAGGAATCGCAGTGGAGAGGAGGGGCATGCCTCA 240
DuxG10    CGCATCCGCGTGTGGTTTCAGAACCGCAGGAATCGCAGTGGAGAGGAGGGGCATGCCTCA 240
DuxG11    CGCATCCGCGTGTGGTTTCAGAACCGCAGGAATCGCAGTGGAGAGGAGGGGCATGCCTCA 240
DuxG12    CGCATCCGCGTGTGGTTTCAGAACCGCAGGAATCGCAGTGGAGAGGAGGGGCATGCCTCA 240
DuxG13    CGCATCCGCGTGTGGTTTCAGAACCGCAGGAATCGCAGTGGAGAGGAGGGGCATGCCTCA 240
DuxG14    CGCATCCGCGTGTGGTTTCAGAACCGCAGGAATCGCAGTGGAGAGGAGGGGCATGCCTCA 240
DuxG15    CGCATCCGCGTGTGGTTTCAGAACCGCAGGAATCGCAGTGGAGAGGAGGGGCATGCCTCA 240
DuxG16    CGCATCCGCGTGTGGTTTCAGAACCGCAGGAATCGCAGTGGAGAGGAGGGGCATGCCTCA 240
DuxG17    CGCATCCGCGTGTGGTTTCAGAACCGCAGGAATCGCAGTGGAGAGGAGGGGCATGCCTCA 240
DuxG18    CGCATCCGCGTGTGGTTTCAGAACCGCAGGAATCGCAGTGGAGAGGAGGGGCATGCCTCA 240
DuxG19    CGCATCCGCGTGTGGTTTCAGAACCGCAGGAATCGCAGTGGAGAGGAGGGGCATGCCTCA 240
DuxG20    CGCATCCGCGTGTGGTTTCAGAACCGCAGGAATCGCAGTGGAGAGGAGGGGCATGCCTCA 240
DuxG21    CGCATCCGCGTGTGGTTTCAGAACCGCAGGAATCGCAGTGGAGAGGAGGGGCATGCCTCA 240
DuxG22    CGCATCCGCGTGTGGTTTCAGAACCGCAGGAATCGCAGTGGAGAGGAGGGGCATGCCTCA 240
DuxG23    CGCATCCGCGTGTGGTTTCAGAACCGCAGGAATCGCAGTGGAGAGGAGGGGCATGCCTCA 240
                  *

Dux       AAGAGGTCCATCAGAGGCTCCAGGCGGCTAGCCTCGCCACAGCTCCAGGAAGAGCTTGGA 300
DuxG1     AAGAGGTCCATCAGAGGCTCCAGGCGGCTAGCCTCGCCACAGCTCCAGGAAGAGCTTGGA 300
DuxG2     AAGAGGTCCATCAGAGGCTCCAGGCGGCTAGCCTCGCCACAGCTCCAGGAAGAGCTTGGA 300
DuxG3     AAGAGGTCCATCAGAGGCTCCAGGCGGCTAGCCTCGCCACAGCTCCAGGAAGAGCTTGGA 300
DuxG4     AAGAGGTCCATCAGAGGCTCCAGGCGGCTAGCCTCGCCACAGCTCCAGGAAGAGCTTGGA 300
DuxG5     AAGAGGTCCATCAGAGGCTCCAGGCGGCTAGCCTCGCCACAGCTCCAGGAAGAGCTTGGA 300
DuxG6     AAGAGGTCCATCAGAGGCTCCAGGCGGCTAGCCTCGCCACAGCTCCAGGAAGAGCTTGGA 300
DuxG7     AAGAGGTCCATCAGAGGCTCCAGGCGGCTAGCCTCGCCACAGCTCCAGGAAGAGCTTGGA 300
DuxG8     AAGAGGTCCATCAGAGGCTCCAGGCGGCTAGCCTCGCCACAGCTCCAGGAAGAGCTTGGA 300
DuxG9     AAGAGGTCCATCAGAGGCTCCAGGCGGCTAGCCTCGCCACAGCTCCAGGAAGAGCTTGGA 300
DuxG10    AAGAGGTCCATCAGAGGCTCCAGGCGGCTAGCCTCGCCACAGCTCCAGGAAGAGCTTGGA 300
DuxG11    AAGAGGTCCATCAGAGGCTCCAGGCGGCTAGCCTCGCCACAGCTCCAGGAAGAGCTTGGA 300
DuxG12    AAGAGGTCCATCAGAGGCTCCAGGCGGCTAGCCTCGCCACAGCTCCAGGAAGAGCTTGGA 300
DuxG13    AAGAGGTCCATCAGAGGCTCCAGGCGGCTAGCCTCGCCACAGCTCCAGGAAGAGCTTGGA 300
DuxG14    AAGAGGTCCATCAGAGGCTCCAGGCGGCTAGCCTCGCCACAGCTCCAGGAAGAGCTTGGA 300
DuxG15    AAGAGGTCCATCAGAGGCTCCAGGCGGCTAGCCTCGCCACAGCTCCAGGAAGAGCTTGGA 300
DuxG16    AAGAGGTCCATCAGAGGCTCCAGGCGGCTAGCCTCGCCACAGCTCCAGGAAGAGCTTGGA 300
DuxG17    AAGAGGTCCATCAGAGGCTCCAGGCGGCTAGCCTCGCCACAGCTCCAGGAAGAGCTTGGA 300
DuxG18    AAGAGGTCCATCAGAGGCTCCAGGCGGCTAGCCTCGCCACAGCTCCAGGAAGAGCTTGGA 300
DuxG19    AAGAGGTCCATCAGAGGCTCCAGGCGGCTAGCCTCGCCACAGCTCCAGGAAGAGCTTGGA 300
DuxG20    AAGAGGTCCATCAGAGGCTCCAGGCGGCTAGCCTCGCCACAGCTCCAGGAAGAGCTTGGA 300
DuxG21    AAGAGGTCCATCAGAGGCTCCAGGCGGCTAGCCTCGCCACAGCTCCAGGAAGAGCTTGGA 300
DuxG22    AAGAGGTCCATCAGAGGCTCCAGGCGGCTAGCCTCGCCACAGCTCCAGGAAGAGCTTGGA 300
DuxG23    AAGAGGTCCATCAGAGGCTCCAGGCGGCTAGCCTCGCCACAGCTCCAGGAAGAGCTTGGA 300

Dux       TCCAGGCCACAGGGTAGAGGCATGCGCTCATCTGGCAGAAGGCCTCGCACTCGACTCACC 360
DuxG1     TCCAGGCCACAGGGTAGAGGCATGCGCTCATCTGGCAGAAGGCCTCGCACTCGACTCACC 360
DuxG2     TCCAGGCCACAGGGTAGAGGCATGCGCTCATCTGGCAGAAGGCCTCGCACTCGACTCACC 360
DuxG3     TCCAGGCCACAGGGTAGAGGCATGCGCTCACCTGGCAGAAGGCCTCGCACTCGACTCACC 360
DuxG4     TCCAGGCCACAGGGTAGAGGCATGCGCTCATCTGGCAGAAGGCCTCGCACTCGACTCACC 360
DuxG5     TCCAGGCCACAGGGTAGAGGCATGCGCTCATCTGGCAGAAGGCCTCGCACTCGACTCACC 360
DuxG6     TCCAGGCCACAGGGTAGAGGCATGCGCTCATCTGGCAGAAGGCCTCGCACTCGACTCACC 360
DuxG7     TCCAGGCCACAGGGTAGAGGTATGCGCTCATCTGGCAGAAGGCCTCGCACTCGACTCACC 360
DuxG8     TCCAGGCCACAGGGTAGAGGCATGCGCTCATCTGGCAGAAGGCCTCGCACTCGACTCACC 360
DuxG9     TCCAGGCCACAGGGTAGAGGCATGCGCTCATCTGGCAGAAGGCCTCGCACTCGACTCACC 360
DuxG10    TCCAGGCCACAGGGTAGAGGCATGCGCTCATCTGGCAGAAGGCCTCGCACTCGACTCACC 360
DuxG11    TCCAGGCCACAGGGTAGAGGCATGCGCTCATCTGGCAGAAGGCCTCGCACTCGACTCACC 360
DuxG12    TCCAGGCCACAGGGTAGAGGCATGCGCTCATCTGGCAGAAGGCCTCGCACTCGACTCACC 360
DuxG13    TCCAGGCCACAGGGTAGAGGCATGCGCTCATCTGGCAGAAGGCCTCGCACTCGACTCACC 360
DuxG14    TCCAGGCCACAGGGTAGAGGCATGCGCTCATCTGGCAGAAGGCCTCGCACTCGACTCACC 360
DuxG15    TCCAGGCCACAGGGTAGAGGCATGCGCTCATCTGGCAGAAGGCCTCGCACTCGACTCACC 360
DuxG16    TCCAGGCCACAGGGTAGAGGCATGCGCTCATCTGGCAGAAGGCCTCGCACTCGACTCACC 360
DuxG17    TCCAGGCCACAGGGTAGAGGCATGCGCTCATCTGGCAGAAGGCCTCGCACTCGACTCACC 360
DuxG18    TCCAGGCCACAGGGTAGAGGCATGCGCTCATCTGGCAGAAGGCCTCGCACTCGACTCACC 360
DuxG19    TCCAGGCCACAGGGTAGAGGCATGCGCTCATCTGGCAGAAGGCCTCGCACTCGACTCACC 360
DuxG20    TCCAGGCCACAGGGTAGAGGCATGCGCTCATCTGGCAGAAGGCCTCGCACTCGACTCACC 360
DuxG21    TCCAGGCCACAGGGTAGAGGCATGCGCTCATCTGGCAGAAGGCCTCGCACTCGACTCACC 360
DuxG22    TCCAGGCCACAGGGTAGAGGCATGCGCTCATCTGGCAGAAGGCCTCGCACTCGACTCACC 360
DuxG23    TCCAGGCCACAGGGTAGAGGCATGCGCTCATCTGGCAGAAGGCCTCGCACTCGACTCACC 360
                              *         *
```

```
Dux     TCGCTACAGCTCAGGATCCTAGGGCAAGCCTTTGAGAGGAACCCACGACCAGGCTTTGCT 420
DuxG1   TCGCTACAGCTCAGGATCCTAGGGCAAGCCTTTGAGAGGAACCCACGACCAGGCTTTGCT 420
DuxG2   TCGCTACAGCTCAGGATCCTAGGGCAAGCCTTTGAGAGGAACCCACGACCAGGCTTTGCT 420
DuxG3   TCGCTACAGCTCAGGATCCTAGGGCAAGCCTTTGAGAGGAACCCACGACCAGGCTTTGCT 420
DuxG4   TCGCTACAGCTCAGGATCCTAGGGCAAGCCTTTGAGAGGAACCCACGACCAGGCTTTGCT 420
DuxG5   TCGCTACAGCTCAGGATCCTAGGGCAAGCCTTTGAGAGGAACCCACGACCAGGCTTTGCT 420
DuxG6   TCGCTACAGCTCAGGATCCTAGGGCAAGCCTTTGAGAGGAACCCACGACCAGGCTTTGCT 420
DuxG7   TCGCTACAGCTCAGGATCCTAGGGCAAGCCTTTGAGAGGAACCCACGACCAGGCTTTGCT 420
DuxG8   TCGCTACAGCTCAGGATCCTAGGGCAAGCCTTTGAGAGGAACCCACGACCAGGCTTTGCT 420
DuxG9   TCGCTACAGCTCAGGATCCTAGGGCAAGCCTTTGAGAGGAATCCACGACCAGGCTTTGCT 420
DuxG10  TCGCTACAGCTCAGGATCCTAGGGCAAGCCTTTGAGAGGAACCCACGACCAGGCTTTGCT 420
DuxG11  TCGCTACAGCTCAGGATCCTAGGGCAAGCCTTTGAGAGGAACCCACGACCAGGCTTTGCT 420
DuxG12  TCGCTACAGCTCAGGATCCTAGGGCAAGCCTTTGAGAGGAACCCACGACCAGGCTTTGCT 420
DuxG13  TCGCTACAGCTCAGGATCCTAGGGCAAGCCTTTGAGAGGAACCCACGACCAGGCTTTGCT 420
DuxG14  TCGCTACAGCTCAGGATCCTAGGGCAAGCCTTTGAGAGGAACCCACGACCAGGCTTTGCT 420
DuxG15  TCGCTACAGCTCAGGATCCTAGGGCAAGCCTTTGAGAGGAACCCACGACCAGGCTTTGCT 420
DuxG16  TCGCTACAGCTCAGGATCCTAGGGCAAGCCTTTGAGAGGAACCCACGACCAGGCTTTGCT 420
DuxG17  TCGCTACAGCTCAGGATCCTAGGGCAAGCCTTTGAGAGGAATCCACGACCAGGCTTTGCT 420
DuxG18  TCGCTACAGCTCAGGATCCTAGGGCAAGCCTTTGAGAGGAACCCACGACCAGGCTTTGCT 420
DuxG19  TCGCTACAGCTCAGGATCCTAGGGCAAGCCTTTGAGAGGAACCCACGACCAGGCTTTGCT 420
DuxG20  TCGCTACAGCTCAGGATCCTAGGGCAAGCCTTTGAGAGGAACCCACGACCAGGCTTTGCT 420
DuxG21  TCGCTACAGCTCAGGATCCTAGGGCAAGCCTTTGAGAGGAACCCACGACCAGGCTTTGCT 420
DuxG22  TCGCTACAGCTCAGGATCCTAGGGCAAGCCTTTGAGAGGAACCCACGACCAGGCTTTGCT 420
DuxG23  TCGCTACAGCTCAGGATCCTAGGGCAAGCCTTTGAGAGGAACCCACGACCAGGCTTTGCT 420
                                                 *

Dux     ACCAGGGAGGAGCTGGCGCGTGACACAGGGTTGCCCGAGGACACGATCCACATATGGTTT 480
DuxG1   ACCAGGGAGGAGCTGGCGCGTGACACAGGGTTGCCCGAGGACACGATCCACATATGGTTT 480
DuxG2   ACCAGGGAGGAGCTGGCGCGTGACACAGGGTTGCCCGAGGACACGATCCACATATGGTTT 480
DuxG3   ACCAGGGAGGAGCTGGCGCGTGACACAGGGTTGCCCGAGGACACGATCCACATATGGTTT 480
DuxG4   ACCAGGGAGGAGCTGGCGCGTGACACAGGGTTGCCCGAGGACACGATCCACATATGGTTT 480
DuxG5   ACCAGGGAGGAGCTGGCGCGTGACACAGGGTTGCCCGAGGACACGATCCACATATGGTTT 480
DuxG6   ACCAGGGAGGAGCTGGCGCGTGACACAGGGTTGCCCGAGGACACGATCCACATATGGTTT 480
DuxG7   ACCAGGGAGGAGCTGGCGCGTGACACAGGGTTGCCCGAGGACACGATCCACATATGGTTT 480
DuxG8   ACCAGGGAGGAGCTGGCGCGTGACACAGGGTTGCCCGAGGACACGATCCACATATGGTTT 480
DuxG9   ACCAGGGAGGAGCTGGCGCGTGACACAGGGTTGCCCGAGGACACGATCCACATATGGTTT 480
DuxG10  ACCAGGGAGGAGCTGGCGCGTGACACAGGGTTGCCCGAGGACACGATCCACATATGGTTT 480
DuxG11  ACCAGGGAGGAGCTGGCGCGTGACACAGGGTTGCCCGAGGACACGATCCACATATGGTTT 480
DuxG12  ACCAGGGAGGAGCTGGCGCGTGACACAGGGATGCCCGAGGACACGATCCACATATGGTTT 480
DuxG13  ACCAGGGAGGAGCTGGCGCGTGACACAGGGTTGCCCGAGGACACGATCCACATATGGTTT 480
DuxG14  ACCAGGGAGGAGCTGGCGCGTGACACAGGGTTGCCCGAGGACACGATCCACATATGGTTT 480
DuxG15  ACCAGGGAGGAGCTGGCGCGTGACACAGGGTTGCCCGAGGACACGATCCACATATGGTTT 480
DuxG16  ACCAGGGAGGAGCTGGCGCGTGACACAGGGTTGCCCGAGGACACGATCCACATATGGTTT 480
DuxG17  ACCAGGGAGGAGCTGGCGCGTGACACAGGGTTGCCCGAGGACACGATCCACATATGGTTT 480
DuxG18  ACCAGGGAGGAGCTGGCGCGTGACACAGGGTTGCCCGAGGACACGATCCACATATGGTTT 480
DuxG19  ACCAGGGAGGAGCTGGCGCGTGACACAGGGTTGCCCGAGGACACGATCCACATATGGTTT 480
DuxG20  ACCAGGGAGGAGCTGGCGCGTGACACAGGGTTGCCCGAGGACACGATCCACATATGGTTT 480
DuxG21  ACCAGGGAGGAGCTGGCGCGTGACACAGGGTTGCCCGAGGACACGATCCACATATGGTTT 480
DuxG22  ACCAGGGAGGAGCTGGCGCGTGACACAGGGTTGCCCGAGGACACGATCCACATATGGTTT 480
DuxG23  ACCAGGGAGGAGCTGGCGCGTGACACAGGGTTGCCCGAGGACACGATCCACATATGGTTT 480
                                      *

Dux     CAAAACCGAAGAGCTCGGCGGCGCCACAGGAGGGGCAGGCCCACAGCTCAAGATCAAGAC 540
DuxG1   CAAAACCGAAGAGCTCGGCGGCGCCACAGGAGGGGCAGGCCCACAGCTCAAGATCAAGAC 540
DuxG2   CAAAACCGAAGAGCTCGGCGGCGCCACAGGAGGGGCAGGCCCACAGCTCAAGATCAAGAC 540
DuxG3   CAAAACCGAAGAGCTCGGCGGCGCCACAGGAGGGGCAGGCCCACAGCTCAAGATCAAGAC 540
DuxG4   CAAAACCGAAGAGCTCGGCGGCGCCACAGGAGGGGCAGGCCCACAGCTCAAGATCAAGAC 540
DuxG5   CAAAACCGAAGAGCTCGGCGGCGCCACAGGAGGGGCAGGCCCACAGCTCAAGATCAAGAC 540
DuxG6   CAAAACCGAAGAGCTCGGCGGCGCCACAGGAGGGGCAGGCCCACAGCTCAAGATCAAGAC 540
DuxG7   CAAAACCAAAGAGCTCGGCGGCCCCACAGGAGGGGCAGGCCCACAGCTCAAGATCAAGAC 540
DuxG8   CAAAACCGAAGAGCTCGGCGGCGCCACAGGAGGGGCAGGCCCACAGCTCAAGATCAAGAC 540
DuxG9   CAAAACCGAAGAGCTCGGCGGCGCCACAGGAGGGGCAGGCCCACAGCTCAAGATCAAGAC 540
DuxG10  CAAAACCGAAGAGCTCGGCGGCGCCACAGGAGGGGCAGGCCCACAGCTCAAGATCAAGAC 540
DuxG11  CAAAACCGAAGAGCTCGGCGGCGCCACAGGAGGGGCAGGCCCACAGCTCAAGATCAAGAC 540
DuxG12  CAAAACCGAAGAGCTCGGCGGCGCCACAGGAGGGGCAGGCCCACAGCTCAAGATCAAGAC 540
DuxG13  CAAAACCGAAGAGCTCGGCGGCGCCACAGGAGGGGCAGGCCCACAGCTCAAGATCAAGAC 540
DuxG14  CAAAACCGAAGAGCTCGGCGGCGCCACAGGAGGGGCAGGCCCACAGCTCAAGATCAAGAC 540
DuxG15  CAAAACCGAAGAGCTCGGCGGCGCCACAGGAGGGGCAGGCCCACAGCTCAAGATCAAGAC 540
DuxG16  CAAAACCGAAGAGCTCGGCGGCGCCACAGGAGGGGCAGGCCCACAGCTCAAGATCAAGAC 540
DuxG17  CAAAACCGAAGAGCTCGGCGGCGCCACAGGAGGGGCAGGCCCACAGCTCAAGATCAAGAC 540
DuxG18  CAAAACCGAAGAGCTCGGCGGCGCCACAGGAGGGGCAGGCCCACAGCTCAAGATCAAGAC 540
DuxG19  CAAAACCGAAGAGCTCGGCGGCGCCACAGGAGGGGCAGGCCCACAGCTCAAGATCAAGAC 540
DuxG20  CAAAACCGAAGAGCTCGGCGGCGCCACAGGAGGGGCAGGCCCACAGCTCAAGATCAAGAC 540
DuxG21  CAAAACCGAAGAGCTCGGCGGCGCCACAGGAGGGGCAGGCCCACAGCTCAAGATCAAGAC 540
DuxG22  CAAAACCGAAGAGCTCGGCGGCGCCACAGAAGGGGCAGGCCCACAGCTCAAGATCAAGAC 540
DuxG23  CAAAACCGAAGAGCTCGGCGGCGCCACAGGAGGGGCAGGCCCACAGCTCAAGATCAAGAC 540
               *              *      *
```

```
Dux     TTGCTGGCGTCACAAGGGTCGGATGGGGCCCCTGCAGGTCCGGAAGGCAGAGAGCGTGAA 600
DuxG1   TTGCTGGCGTCACAAGGGTCGGATGGGGCCCCTGCAGGTCCGGAAGGCAGAGAGCGTGAA 600
DuxG2   TTGCTGGCGTCACAAGGGTCGGATGGGGCCCCTGCAGGTCCGGAAGGCAGAGAGCGTGAA 600
DuxG3   TTGCTGGCGTCACAAGGGTCGGATGGGGCCCCTGCAGGTCCGGAAGGCAGAGAGCGTGAA 600
DuxG4   TTGCTGGCGTCACAAGGGTCGGATGGGGCCCCTGCAGGTCCGGAAGGCAGAGAGCGTGAA 600
DuxG5   TTGCTGGCGTCACAAGGGTCGGATGGGGCCCCTGCAGGTCCGGAAGGCAGAGAGCGTGAA 600
DuxG6   TTGCTGGCGTCACAAGGGTCGGATGGGGCCCCTGCAGGTCCGGAAGGCAGAGAGCGTGAA 600
DuxG7   TTGCTGGCGTCACAAGGGTGGGACGGGGCCCATGCAGGTCCGGAAGGCAGAGAGCGTGAA 600
DuxG8   TTGCTGGCGTCACAAGGGTCGGATGGGGCCCCTGCAGGTCCGGAAGGCAGAGAGCGTGAA 600
DuxG9   TTGCTGGCGTCACAAGGGTCGGATGGGGCCCCTGCAGGTCCGGAAGGCAGAGAGCGTGAA 600
DuxG10  TTGCTGGCGTCACAAGGGTCGGATGGGGCCCCTGCAGGTCCGGAAGGCAGAGAGCGTGAA 600
DuxG11  TTGCTGGCGTCACAAGGGTCGGATGGGGCCCCTGCAGGTCCGGAAGGCAGAGAGCGTGAA 600
DuxG12  TTGCTGGCGTCACAAGGGTCGGATGGGGCCCCTGCAGGTCCGGAAGGCAGAGAGCGTGAA 600
DuxG13  TTGCTGGCGTCACAAGGGTCGGATGGGGCCCCTGCAGGTCCGGAAGGCAGAGAGCGTGAA 600
DuxG14  TTGCTGGCGTCACAAGGGTCGGATGGGGCCCCTGCAGGTCCGGAAGGCAGAGAGCGTGAA 600
DuxG15  TTGCTGGCGTCACAAGGGTCGGATGGGGCCCCTGCAGGTCCGGAAGGCAGAGAGCGTGAA 600
DuxG16  TTGCTGGCGTCACAAGGGTCGGATGGGGCCCCTGCAGGTCCGGAAGGCAGAGAGCGTGAA 600
DuxG17  TTGCTGGCGTCACAAGGGTCGGATGGGGCCCCTGCAGGTCCGGAAGGCAGAGAGCGTGAA 600
DuxG18  TTGCTGGCGTCACAAGGGTCGGATGGGGCCCCTGCAGGTCCGGAAGGCAGAGAGCGTGAA 600
DuxG19  TTGCTGGCGTCACAAGGGTCGGATGGGGCCCCTGCAGGTCCGGAAGGCAGAGAGCGTGAA 600
DuxG20  TTGCTGGTGTCACAAGGGTCGGATGGGGCCCCTGCAGGTCCGGAAGGCAGAGAGCGTGAA 600
DuxG21  TTGCTGGCGTCACAAGGGTCGGATGGGGCCCCTGCAGGTCCGGAAGGCAGAGAGCGTGAA 600
DuxG22  TTGCTGGCATCACAAGGGTCGGATGGGGCCCCTGCAGGTCCGGAAGGCAGAGAGCGTGAA 600
DuxG23  TTGCTGGCGTCACAAGGGTCGGATGGGGCCCCTGCAGGTCCGGAAGGCAGAGAGCGTGAA 600
               **          *   *       *

Dux     GGTGCCCAGGAGAACTTGTTGCCACAGGAAGAAGCAGGAAGTACGGGCATGGATACCTCG 660
DuxG1   GGTGCCCAGGAGAACTTGTTGCCACAGGAAGAAGCAGGAAGTACGGGCATGGATACCTCG 660
DuxG2   GGTGCCCAGGAGAACTTGTTGCCACAGGAAGAAGCAGAAAGTACGGGCATGGATACCTCG 660
DuxG3   GGTGCCCAGGAGAACTTGTTGCCACAGGAAGAAGCAGGAAGTACGGGCATGGATACCTCG 660
DuxG4   GGTGCCCAGGAGAACTTGTTGCCACAGGAAGAAGCAGGAAGTACGGGCATGGATACCTCG 660
DuxG5   GGTGCCCAGGAGAACTTGTTGCCACAGGAAGAAGCAGGAAGTACGGGCATGGATACCTCG 660
DuxG6   GGTGCCCAGGAGAACTTGTTGCCACAGGAAGAAGCAGGAAGTACGGGCATGGATACCTCG 660
DuxG7   GGTGCCCAGGAGAACTTGTTGCCACAGGAAGAAGCAGGAAGTACGGGCATGGATACCTCG 660
DuxG8   GGTGCCCAGGAGAACTTGTTGCCACAGGAAGAAGCAGGAAGTACGGGCATGGATACCTCG 660
DuxG9   GGTGCCCAGGAGAACTTGTTGCCACAGGAAGAAGCAGGAAGTACGGGCATGGATACCTCG 660
DuxG10  GGTGCCCAGGAGAACTTGTTGCCACAGGAAGAAGCAGGAAGTACCGGCATGGATACCTCG 660
DuxG11  GGTGCCCAGGAGAACTTGTTGCCACAGGAAGAAGCAGGAAGTACGGGCATGGATACCTCG 660
DuxG12  GGTGCCCAGGAGAACTTGTTGCCACAGGAAGAAGCAGGAAGTACGGGCATGGATACCTCG 660
DuxG13  GGTGCCCAGGAGAACTTGTTGCCACAGGAAGAAGCAGGAAGTACGGGCATGGATACCTCG 660
DuxG14  GGTGCCCAGGAGAACTTGTTGCCACAGGAAGAAGCAGGAAGTACGGGCATGGATACCTCG 660
DuxG15  GGTGCCCAGGAGAACTTGTTGCCACAGGAAGAAGCAGGAAGTACGGGCATGGATACCTCG 660
DuxG16  GGTGCCCAGGAGAACTTGTTGCCACAGGAAGAAGCAGGAAGTACCGGCATGGATACCTCG 660
DuxG17  GGTGCCCAGGAGAACTTGTTGCCACAGGAAGAAGCAGGAAGTACGGGCATGGATACCTCG 660
DuxG18  GGTGCCCAGGAGAACTTGTTGCCACAGGAAGAAGCAGGAAGTACGGGCATGGATACCTCG 660
DuxG19  GGTGCCCAGGAGAACTTGTTGCCACAGGAAGAAGCAGGAAGTACGGGCATGGATACCTCG 660
DuxG20  GGTGCCCAGGAGAACTTGTTGCCACAGGAAGAAGCAGGAAGTACGGGCATGGATACCTCG 660
DuxG21  GGTGCCCAGGAGAACTTGTTGCCACAGGAAGAAGCAGAAAGTACGGGCATGGATACCTCG 660
DuxG22  GGTGCCCAGGAGAACTTGTTGCCACAGGAAGAAGCAGGAAGTACGGGGATGGATACCTCG 660
DuxG23  GGTGCCCAGGAGAACTTGTTGCCACAGGAAGAAGCAGGAAGTACGGGCATGGATACCTCG 660
                                             *      *  *

Dux     AGCCCTAGCGACTTGCCCTCCTTCTGCGGAGAGTCCCAGCCTTTCCAAGTGGCACAGCCC 720
DuxG1   AGCCCTAGCGACTTGCCCTCCTTCTGCGGAGAGTCCCAGCCTTTCCAAGTGGCACAGCCC 720
DuxG2   AGCCCTAGCGACTTGCCCTCCTTCTGCGGAGAGTCCCAGCCTTTCCAAGTGGCACAGCCC 720
DuxG3   AGCCCTAGCGACTTGCCCTCCTTCTGCGGAGAGTCCCAGCCTTTCCAAGTGGCACAGCCC 720
DuxG4   AGCCCTAGCGACTTGCCCTCCTTCTGCGGAGAGTCCCAGCCTTTCCAAGTGGCACAGCCC 720
DuxG5   AGCCCTAGCGACTTGCCCTCCTTCTGCGGAGAGTCCCAGCCTTTCCAAGTGGCACAGCCC 720
DuxG6   AGCCCTAGCGACTTGCCCTCCTTCTGCGGAGAGTCCCAGCCTTTCCAAGTGGCACAGCCC 720
DuxG7   AGCCCTAGCGACTTGCCCTCCTTCTGCGGAGAGTCCCAGCCTTTCCAAGTGGCACAGCCC 720
DuxG8   AGCCCTAGCGACTTGCCCTCCTTCTGCGGAGAGTCCCAGCCTTTCCAAGTGGCACAGCCC 720
DuxG9   AGCCCTAGCGACTTGCCCTCCTTCTGCGGAGAGTCCCAGCCTTTCCAAGTGGCACAGCCC 720
DuxG10  AGCCCTAGCGACTTGCCCTCCTTCTGCGGAGAGTCCCAGCCTTTCCAAGTGGCACAGCCC 720
DuxG11  AGCCCTAGCGACTTGCCCTCCTTCTGCGGAGAGTCCCAGCCTTTCCAAGTGGCACAGCCC 720
DuxG12  AGCCCTAGCGACTTGCCCTCCTTCTGCGGAGAGTCCCAGCCTTTCCAAGTGGCACAGCCC 720
DuxG13  AGCCCTAGCGACTTGCCCTCCTTCTGCGGAGAGTCCCAGCCTTTCCAAGTGGCACAGCCC 720
DuxG14  AGCCCTAGCGACTTGCCCTCCTTCTGCGGAGAGTCCCAGCCTTTCCAAGTGGCACAGCCC 720
DuxG15  AGCCCTAGCGACTTGCCCTCCTTCTGCGGAGAGTCCCAGCCTTTCCAAGTGGCACAGCCC 720
DuxG16  AGCCCTAGCGACTTGCCCTCCTTCTGCGGAGAGTCCCAGCCTTTCCAAGTGGCACAGCCC 720
DuxG17  AGCCCTAGCGACTTGCCCTCCTTCTGCGGAGAGTCCCAGCCTTTCCAAGTGGCACAGCCC 720
DuxG18  AGCCCTAGCGACTTGCCCTCCTTCTGCGGAGAGTCCCAGCCTTTCCAAGTGGCACAGCCC 720
DuxG19  AGCCCTAGCGACTTGCCCTCCTTCTGCGGAGAGTCCCAGCCTTTCCAAGTGGCACAGCCC 720
DuxG20  AGCCCTAGCGACTTGCCCTCCTTCTGCGGAGAGTCCCAGCCTTTCCAAGTGGCACAGCCC 720
DuxG21  AGCCCTAGCGACTTGCCCTCCTTCTGCGGAGAGTCCCAGCCTTTCCAAGTGGCACAGCCC 720
DuxG22  AGCCCTAGCGACTTGCCCTCCTTCTGCGGAGAGTCCCAGCCTTTCCAAGTGGCACAGCCC 720
DuxG23  AGCCCTAGCGACTTGCCCTCCTTCTGCGGAGAGTCCCAGCCTTTCCAAGTGGCACAGCCC 720
```

Dux	CGTGGAGCAGGCCAACAAGAGGCCCCCACTCGAGCAGGCAACGCAGGCTCTCTGGAACCC	780
DuxG1	CGTGGAGCAGGCCAACAAGAGGCCCCCACTC**A**AGCAGGCAACGCAGGCTCTCTGGAACCC	780
DuxG2	CGTGGAGCAGGCCAACAAGAGGCCCCCACTCGAGCAGGCAACGCAGGCTCTCTGGAACCC	780
DuxG3	CGTGGAGCAGGCCAACAAGAGGCCCCCACTCGAGCAGGCAACGCAGGCTCTCTGGAACCC	780
DuxG4	CGTGGAGCAGGCCAACAAGAGGCCCCCACTCGAGCAGGCAACGCAGGCTCTCTGGAACCC	780
DuxG5	CGTGGAGCAGGCCAA**A**AAGAGG**A**CCCCACTCGAGCAGGCAACGCAGGCTCTCTGGAACCC	780
DuxG6	CGTGGAGCAGGCCAACAAGAGGCCCCCACTCGAGCAGGCAACGCAGGCTCTCTGGAACCC	780
DuxG7	CGTGGAGCAGGCCAACAAGAGGCCCCCACTCGAGCAGGCAACGCAGGCTCTCTGGAACCC	780
DuxG8	CGTGGAGCAGGCCAACAAGAGGCCCCCACTCGAGCAGGCAACGCAGGCTCTCTGGAACCC	780
DuxG9	CGTGGAGCAGGCCAACAAGAGGCCCCCACTC**A**AGCAGGCAACGCAGGCTCTCTGGAACCC	780
DuxG10	CGTGGAGCAGGCCAACAAGAGGCCCCCACTC**A**AGCAGGCAACGCAGGCTCTCTGGAACCC	780
DuxG11	CGTGGAGCAGGCCAACAAGAGGCCCCCACTCGAGCAGGCAACGCAGGCTCTCTGGAACCC	780
DuxG12	CGTGGAGCAGGCCAACAAGAGGCCCCCACTCGAGCAGGCAACGCAGGCTCTCTGGAACCC	780
DuxG13	CGTGGAGCAGGCCAACAAGAGGCCCCCACTC**A**AGCAGGCAACGCAGGCTCTCTGGAACCC	780
DuxG14	CGTGGAGCAGGCCAA**A**AAGAGG**A**CCCCACTCGAGCAGGCAACGCAGGCTCTCTGGAACCC	780
DuxG15	CGTGGAGCAGGCCAACAAGAGGCCCCCACTC**A**AGCAGGCAACGCAGGCTCTCTGGAACCC	780
DuxG16	CGTGGAGCAGGCCAACAAGAGGCCCCCACTC**A**AGCAGGCAACGCAGGCTCTCTGGAACCC	780
DuxG17	CGTGGAGCAGGCCAACAAGAGGCCCCCACTC**A**AGCAGGCAACGCAGGCTCTCTGGAACCC	780
DuxG18	CGTGGAGCAGGCCAACAAGAGGCCCCCACTC**A**AGCAGGCAACGCAGGCTCTCTGGAACCC	780
DuxG19	CGTGGAGCAGGCCAACAAGAGGCCCCCACTC**A**AGCAGGCAACGCAGGCTCTCTGGAACCC	780
DuxG20	CGTGGAGCAGGCCAACAAGAGGCCCCCACTCGAGCAGGCAACGCAGGCTCTCTGGAACCC	780
DuxG21	CGTGGAGCAGGCCAACAAGAGGCCCCCACTCGAGCAGGCAACGCAGGCTCTCTGGAACCC	780
DuxG22	CGTGGAGCAGGCCAACAAGAGGCCCCCACTC**A**AGCAGGCAACGCAGGCTCTCTGGAACCC	780
DuxG23	CGTGGAGCAGGCCAACAAGAGGCCCCCACTC**A**AGCAGGCAACGCAGGCTCTCTGGAACCC	780
	* * *	

Dux	CTCCTTGATCAGCTGCTGGATGAAGTCCAAGTAGAAGAGCCTGCTCCAGCCCCTCTGAAT	840
DuxG1	CTCCTTGATCAGCTGCTGGATGAAGTCCAAGTAGAAGAGCCTGCTCCAGCCCCTCTGAAT	840
DuxG2	CTCCTTGATCAGCTGCTGGATGAAGTCCAAGTAGAAGAGCCTGCTCCAGCCCCTCTGAAT	840
DuxG3	CTCCTTGATCAGCTGCTGGATGAAGTCCAAGTAGAAGAGCCTGCTCCAGCCCCTCTGAAT	840
DuxG4	CTCCTTGATCAGCTGCTGGATGAAGTCCAAGTAGAAGAGCCTGCTCCAGCCCCTCTGAAT	840
DuxG5	CTCCTTGATCAGCTGCTGGATGAAGTCCAAGTAGAAGAGCCTGCTCCAGCCCCTCTGAAT	840
DuxG6	CTCCTTGATCAGCTGCTGGATGAAGTCCAAGTAGAAGAGCCTGCTCCAGCCCCTCTGAAT	840
DuxG7	CTCCTTGATCAGCTGCTGGATGAAGTCCAAGTAGAAGAGCCTGCTCCAGCCCCTCTGAAT	840
DuxG8	CTCCTTGATCAGCTGCTGGATGAAGTCCAAGTAGAAGAGCCTGCTCCAGCCCCTCTGAAT	840
DuxG9	CTCCTTGATCAGCTGCTGGATGAAGTCCAAGTAGAAGAGCCTGCTCCAGCCCCTCTGAAT	840
DuxG10	CTCCTTGATCAGCTGCTGGATGAAGTCCAAGTAGAAGAGCCTGCTCCAGCCCCTCTGAAT	840
DuxG11	CTCCTTGATCAGCTGCTGGATGAAGTCCAAGTAGAAGAGCCTGCTCCAGCCCCTCTGAAT	840
DuxG12	CTCCTTGATCAGCTGCTGGATGAAGTCCAAGTAGAAGAGCCTGCTCCAGCCCCTCTGAAT	840
DuxG13	CTCCTTGATCAGCTGCTGGATGAAGTCCAAGTAGAAGAGCCTGCTCCAGCCCCTCTGAAT	840
DuxG14	CTCCTTGATCAGCTGCTGGATGAAGTCCAAGTAGAAGAGCCTGCTCCAGCCCCTCTGAAT	840
DuxG15	CTCCTTGATCAGCTGCTGGATGAAGTCCAAGTAGAAGAGCCTGCTCCAGCCCCTCTGAAT	840
DuxG16	CTCCTTGATCAGCTGCTGGATGAAGTCCAAGTAGAAGAGCCTGCTCCAGCCCCTCTGAAT	840
DuxG17	CTCCTTGATCAGCTGCTGGATGAAGTCCAAGTAGAAGAGCCTGCTCCAGCCCCTCTGAAT	840
DuxG18	CTCCTTGATCAGCTGCTGGATGAAGTCCAAGTAGAAGAGCCTGCTCCAGCCCCTCTGAAT	840
DuxG19	CTCCTTGATCAGCTGCTGGATGAAGTCCAAGTAGAAGAGCCTGCTCCAGCCCCTCTGAAT	840
DuxG20	CTCCTTGATCAGCTGCTGGATGAAGTCCAAGTAGAAGAGCCTGCTCCAGCCCCTCTGAAT	840
DuxG21	CTCCTTGATCAGCTGCTGGATGAAGTCCAAGTAGAAGAGCCTGCTCCAGCCCCTCTGAAT	840
DuxG22	CTCCTTGATCAGCTGCTGGATGAAGTCCAAGTAGAAGAGCCTGCTCCAGCCCCTCTGAAT	840
DuxG23	CTCCTTGATCAGCTGCTGGATGAAGTCCAAGTAGAAGAGCCTGCTCCAGCCCCTCTGAAT	840

Dux	TTGGATGGAGACCCTGGTGGCAGGGTGCATGAAGGTTCCCAGGAGAGCTTTTGGCCACAG	900
DuxG1	TTGGATGGAGACCCTGGTGGCAGGGTGCATGAAGGTTCCCAGGAGAGCTTTTGGCCACAG	900
DuxG2	TTGGATGGAGACCCTGGTGGCAGGGTGCATGAAGGTTCCCAGGAGAGCTTTTGGCCACAG	900
DuxG3	TTGGATGGAGACCCTGGTGGCAGGGTGCATGAAGGTTCCCAGGAGAGCTTT**A**GGCCACAG	900
DuxG4	TTGGATGGAGACCCTGGTGGCAGGGTGCATGAAGGTTCCCAGGAGAGCTTTTGGCCACAG	900
DuxG5	TTGGATGGAGACCCTGGTGGCAGGGTGCATGAAGGTTCCCAGGAGAGCTTTTGGCCACAG	900
DuxG6	TTGGATGGAGACCCTGGTGGCAGGGTGCATGAAGGTTCCCAGGAGAGCTTT**A**GGCCACAG	900
DuxG7	TTGGATGGAGACCCTGGTGGCAGGGTGCATGAAGGTTCCCAGGAGAGCTTTTGGCCACAG	900
DuxG8	TTGGATGGAGACCCTGGTGGCAGGGTGCATGAAGGTTCCCAGGAGAGCTTT**A**GGCCACAG	900
DuxG9	TTGGATGGAGACCCTGGTGGCAGGGTGCATGAAGGTTCCCAGGAGAGCTTTTGGCCACAG	900
DuxG10	TTGGATGGAGACCCTGGTGGCAGGGTGCATGAAGGTTCCCAGGAGAGCTTTTGGCCACAG	900
DuxG11	TTGGATGGAGACCCTGGTGGCAGGGTGCATGAAGGTTCCCAGGAGAGCTTT**A**GGCCACAG	900
DuxG12	TTGGATGGAGACCCTGGTGGCAGGGTGCATGAAGGTTCCCAGGAGAGCTTTTGGCCACAG	900
DuxG13	TTGGATGGAGACCCTGGTGGCAGGGTGCATGAAGGTTCCCAGGAGAGCTTTTGGCCACAG	900
DuxG14	TTGGATGGAGACCCTGGTGGCAGGGTGCATGAAGGTTCCCAGGAGAGCTTTTGGCCACAG	900
DuxG15	TTGGATGGAGACCCTGGTGGCAGGGTGCATGAAGGTTCCCAGGAGAGCTTTTGGCCACAG	900
DuxG16	TTGGATGGAGACCCTGGTGGCAGGGTG**T**ATGAAGGTTCCCAGGAGAGCTTTTGGCCACAG	900
DuxG17	TTGGATGGAGACCCTGGTGGCAGGGTGCATGAAGGTTCCCAGGAGAGCTTTTGGCCACAG	900
DuxG18	TTGGATGGAGACCCTGGTGGCAGGGTGCATGAAGGTTCCCAGGAGAGCTTTTGGCCACAG	900
DuxG19	TTGGATGGAGACCCTGGTGGCAGGGTGCATGAAGGTTCCCAGGAGAGCTTTTGGCCACAG	900
DuxG20	TTGGATGGAGACCCTGGTGGCAGGGTGCATGAAGGTTCCCAGGAGAGCTTTTGGCCACAG	900
DuxG21	TTGGATGGAGACCCTGGTGGCAGGGTGCATGAAGGTTCCCAGGAGAGCTTTTGGCCACAG	900
DuxG22	TTGGATGGAGACCCTGGTGGCAGGGTGCATGAAGGTTCCCAGGAGAGCTTTTGGCCACAG	900
DuxG23	TTGGATGGAGACCCTGGTGGCAGGGT**T**CATGAAGGTTCCCAGGAGAGCTTTTGGCCACAG	900
	* * *	

```
Dux      GAAGAAGCAGGAAGTACAGGCATGGATACTTCTAGCCCCAGCGACTCAAACTCCTTCTGC 960
DuxG1    GAAGAAGCAGGAAGTACAGGCATGGATACTTCTAGCCCCAGCGACTCAAACTCCTTCTGC 960
DuxG2    GAAGAAGCAGGAAGTACAGGCATGGATACTTCTAGCCCCAGCGACTCAAACTCCTTCTGC 960
DuxG3    GAAGAAGCAGGAAGTACAGGCATGGATACTTCTAGCCCCAGCGACTCAAACTCCTTCTGC 960
DuxG4    GAAGAAGCAGGAAGTACAGGCATGGATACTTCTAGCCCCAGCGACTCAAACTCCTTCTGC 960
DuxG5    GAAGAAGCAGGAAGTACAGGCATGGATACTTCTAGCCCCAGCGACTCAAACTCCTTCTGC 960
DuxG6    GAAGAAGCAGGAAGTACAGGCATGGATACTTCTAGCCCCAGCGACTCAAACTCCTTCTGC 960
DuxG7    GAAGAAGCAGGAAGTACAGGCATGGATACTTCTAGCCCCAGCGACTCAAACTCCTTCTGC 960
DuxG8    GAAGAAGCAGGAAGTACAGGCATGGATACTTCTAGCCCCAGCGACTCAAACTCCTTCTGC 960
DuxG9    GAAGAAGCAGGAAGTACAGGCATGGATACTTCTAGCCCCAGCGACTCAAACTCCTTCTGC 960
DuxG10   GAAGAAGCAGGAAGTACAGGCATGGATACTTCTAGCCCCAGCGACTCAAACTCCTTCTGC 960
DuxG11   GAAGAAGCAGGAAGTACAGGCATGGATACTTCTAGCCCCAGCGACTCAAACTCCTTCTGC 960
DuxG12   GAAGAAGCAGGAAGTACAGGCATGGATACTTCTAGCCCCAGCGACTCAAACTCCTTCTGC 960
DuxG13   GAAGAAGCAGGAAGTACAGGCATGGATACTTCTAGCCCCAGCGACTCAAACTCCTTCTGC 960
DuxG14   GAAGAAGCAGGAAGTACAGGCATGGATACTTCTAGCCCCAGCGACTCAAACTCCTTCTGC 960
DuxG15   GAAGAAGCAGGAAGTACAGGCCTGGATACTTCTAGCCCCAGCGACTCAAACTCCTTCTGC 960
DuxG16   GAAGAAGCAGGAAGTACAGGCCTGGATACTTCAAGCCCCAGCGACTCAAACTCCTTCTGC 960
DuxG17   GAAGAAGCAGGAAGTACAGGCATGGATACTTCTAGCCCCAGCGACTCAAACTCCTTCTGC 960
DuxG18   GAAGAAGCAGGAAGTACAGGCATGGATACTTCTAGCCCCAGCGACTCAAACTCCTTCTGC 960
DuxG19   GAAGAAGCAGGAAGTACAGGCATGGATACTTCTAGCCCCAGCGACTCAAACTCCTTCTGC 960
DuxG20   GAAGAAGCAGGAAGTACAGGCATGGATACTTCTAGCCCCAGCGACTCAAACTCCTTCTGC 960
DuxG21   GAAGAAGCAGGAAGTACAGGCATGGATACTTCTAGCCCCAGCGACTCAAACTCCTTCTGC 960
DuxG22   GAAGAAGCAGGAAGTACAGGCATGGATACTTCTAGCCCCAGCGACTCAAACTCCTTCTGC 960
DuxG23   GAAGAAGCAGGAAGTACAGGCATGGATACTTCTAGCCCCAGCGACTCAAACTCCTTCTGC 960
                              *           *

Dux      AGAGAGTCCCAGCCTTCCCAAGTGGCACAGCCCTGTGGAGCGGGCCAAGAAGATGCCCGC 1020
DuxG1    AGAGAGTCCCAGCCTTCCCAAGTGGCACAGCCCTGTGGAGCGGGCCAAGAAGATGCCCGC 1020
DuxG2    AGAGAGTCCCAGCCTTCCCAAGTGGCACAGCCCTGTGGAGCGGGCCAAGAAGATGCCCGC 1020
DuxG3    AGAGAGTCCCAGCCTTCCCAAGTGGCACAGCCCTGTGGAGCGGGCCAAGAAGATGCCCGC 1020
DuxG4    AGAGAGTCCCAGCCTTCCCAAGTGGCACAGCCCTGTGGAGCGGGCCAAGAAGATGCCCGC 1020
DuxG5    AGAGAGTCCCAGCCTTCCCAAGTGGCACAGCCCTGTGGAGCGGGCCAAGAAGATGCCCGC 1020
DuxG6    AGAGAGTCCCAGCCTTCCCAAGTGGCACAGCCCTGTGGAGCGGGCCAAGAAGATGCCCGC 1020
DuxG7    AGAGAGTCCCAGCCTTCCCAAGTGGCACAGCCCTGTGGAGCGGGCCAAGAAGATGCCCGC 1020
DuxG8    AGAGAGTCCCAGCCTTCCCAAGTGGCACAGCCCTGTGGAGCGGGCCAAGAAGATGCCCGC 1020
DuxG9    AGAGAGTCCCAGCCTTCCCAAGTGGCACAGCCCTGTGGAGCGGGCCAAGAAGATGCCCGC 1020
DuxG10   AGAGAGTCCCAGCCTTCCCAAGTGGCACAGCCCTGTGGAGCGGGCCAAGAAGATGCCCGC 1020
DuxG11   AGAGAGTCCCAGCCTTCCCAAGTGGCACAGCCCTGTGGAGCGGGCCAAGAAGATGCCCGC 1020
DuxG12   AGAGAGTCCCAGCCTTCCCAAGTGGCACAGCCCTGTGGAGCGGGCCAAGAAGATGCCCGC 1020
DuxG13   AGAGAGTCCCAGCCTTCCCAAGTGGCACAGCCCTGTGGAGCGGGCCAAGAAGATGCCCGC 1020
DuxG14   AGAGAGTCCCAGCCTTCCCAAGTGGCACAGCCCTGTGGAGCGGGCCAAGAAGATGCCCGC 1020
DuxG15   AGAGAGTCCCAGCCTTCCCAAGTGGCACAGCCCTGTGGAGCGGGCCAAGAAGATGCCCGC 1020
DuxG16   AGAGAGTCCCAGCCTTCCCAAGTGGCACAGCCCTGTGGAGCGGGCCAAGAAGATGCCCGC 1020
DuxG17   AGAGAGTCCCAGCCTTCCCAAGTGGCACAGCCCTGTGGAGCGGGCCAAGAAGATGCCCGC 1020
DuxG18   AGAGAGTCCCAGCCTTCCCAAGTGGCACAGCCCTGTGGAGCGGGCCAAGAAGATGCCCGC 1020
DuxG19   AGAGAGTCCCAGCCTTCCCAAGTGGCACAGCCCTGTGGAGCGGGCCAAGAAGATGCCCGC 1020
DuxG20   AGAGAGTCCCAGCCTTCCCAAGTGGCACAGCCCTGTGGAGCGGGCCAAGAAGATGCCCGC 1020
DuxG21   AGAGAGTCCCAGCCTTCCCAAGTGGCACAGCCCTGTGGAGCGGGCCAAGAAGATGCCCGC 1020
DuxG22   AGAGAGTCCCAGCCTTCCCAAGTGGCACAGCCCTGTGGAGCGGGCCAAGAAGATGCCCGC 1020
DuxG23   AGAGAGTCCCAGCCTTCCCAAGTGGCACAGCCCTGTGGAGCGGGCCAAGAAGATGCCCGC 1020

Dux      ACTCAAGCAGACAGCACAGGCCCTCTGGAACTCCTCCTCCTTGATCAACTGCTGGACGAA 1080
DuxG1    ACTCAAGCAGACAGCACAGGCCCTCTGGAACTCCTCCTCCTTGATCAACTGCTGGACGAA 1080
DuxG2    ACTCAAGCAGACAGCACAGGCCCTCTGGAACTCCTCCTCCTTGATCAACTGCTGGACGAA 1080
DuxG3    ACTCAAGCAGACAGCACAGGCCCTCTGGAACTCCTCCTCCTTGATCAACTGCTGGACGAA 1080
DuxG4    ACTCAAGCAGACAGCACAGGCCCTCTGGAACTCCTCCTCCTTGATCAACTGCTGGACGAA 1080
DuxG5    ACTCAAGCAGACAGCACAGGCCCTCTGGAACTCCTCCTCCTTGATCAACTGCTGGACGAA 1080
DuxG6    ACTCAAGCAGACAGCACAGGCCCTCTGGAACTCCTCCTCCTTGATCAACTGCTGGACGAA 1080
DuxG7    ACTCAAGCAGACAGCACAGGCCCTCTGGAACTCCTCCTCCTTGATCAACTGCTGGACGAA 1080
DuxG8    ACTCAAGCAGACAGCACAGGCCCTCTGGAACTCCTCCTCCTTGATCAACTGCTGGACGAA 1080
DuxG9    ACTCAAGCAGACAGCACAGGCCCTCTGGAACTCCTCCTCCTTGATCAACTGCTGGACGAA 1080
DuxG10   ACTCAAGCAGACAGCACAGGCCCTCTGGAACTCCTCCTCCTTGATCAACTGCTGGACGAA 1080
DuxG11   ACTCAAGCAGACAGCACAGGCCCTCTGGAACTCCTCCTCCTTGATCAACTGCTGGACGAA 1080
DuxG12   ACTCAAGCAGACAGCACAGGCCCTCTGGAACTCCTCCTCCTTGATCAACTGCTGGACGAA 1080
DuxG13   ACTCAAGCAGACAGCACAGGCCCTCTGGAACTCCTCCTCCTTAATCAACTGCTGGAAGAA 1080
DuxG14   ACTCAAGCAGACAGCACAGGCCCTCTGGAACTCCTCCTCCTTGATCAACTGCTGGACGAA 1080
DuxG15   ACTCAAGCAGACAGCACAGGCCCTCTGGAACTCCTCCTCCTTGATCAACTGCTGGACGAA 1080
DuxG16   ACTCAAGCAGACAGCACAGGCCCTCTGGAACTCCTCCTCCTTGATCAACTGCTGGACGAA 1080
DuxG17   ACTCAAGCAGACAGCACAGGCCCTCTGGAACTCCTCCTCCTTGATCAACTGCTGGACGAA 1080
DuxG18   ACTCAAGCAGACAGCACAGGCCCTCTGGAACTCCTCCTCCTTGATCAACTGCTGGACGAA 1080
DuxG19   ACTCAAGCAGACAGCACAGGCCCTCTGGAACTCCTCCTCCTTGATCAACTGCTGGACGAA 1080
DuxG20   ACTCAAGCAGACAGCACAGGCCCTCTGGAACTCCTCCTCCTTGATCAACTGCTGGACGAA 1080
DuxG21   ACTCAAGCAGACAGCACAGGCCCTCTGGAACTCCTCCTCCTTGATCAACTGCTGGACGAA 1080
DuxG22   ACTCAAGCAGACAGCACAGGCCCTCTGGAACTCCTCCTCCTTGATCAACTGCTGGACGAA 1080
DuxG23   ACTCAAGCAGACAGCACAGGCCCTCTGGAACTCCTCCTCCTTGATCAACTGCTGGACGAA 1080
                                                   *              *
```

```
Dux       GTCCAAAAGGAAGAGCATGTGCCAGTCCCACTGGATTGGGGTAGAAATCCTGGCAGCAGG 1140
DuxG1     GTCCAAAAGGAAGAGCATGTGCCAGTCCCACTGGATTGGGGTAGAAATCCTGGCAGCAGG 1140
DuxG2     GTCCAAAAGGAAGAGCATGTGCCAGTCCCACTGGATTGGGGTAGAAATCCTGGCAGCAGG 1140
DuxG3     GTCCAAAAGGAAGAGCATGTGCCAGTCCCACTGGATTGGGGTAGAAATCCTGGCAGCAGG 1140
DuxG4     GTCCAAAAGGAAGAGCATGTGCCAGTCCCACTGGATTGGGGTAGAAATCCTGGCAGCAGG 1140
DuxG5     GTCCAAAAGGAAGAGCATGTGCCAGTCCCACTGGATTGGGGTAGAAATCCTGGCAGCAGG 1140
DuxG6     GTCCAAAAGGAAGAGCATGTGCCAGTCCCACTGGATTGGGGTAGAAATCCTGGCAGCAGG 1140
DuxG7     GTCCAAAAGGAAGAGCATGTGCCAGTCCCACTGGATTGGGGTAGAAATCCTGGCAGCAGG 1140
DuxG8     GTCCAAAAGGAAGAGCATGTGCCAGTCCCACTGGATTGGGGTAGAAATCCTGGCAGCAGG 1140
DuxG9     GTCCAAAAGGAAGAGCATGTGCCAGTCCCACTGGATTGGGGTAGAAATCCTGGCAGCAGG 1140
DuxG10    GTCCAAAAGGAAGAGCATGTGCCAGTCCCACTGGATTGGGGTAGAAATCCTGGCAGCAGG 1140
DuxG11    GTCCAAAAGGAAGAGCATGTGCCAGTCCCACTGGATTGGGGTAGAAATCCTGGCAGCAGG 1140
DuxG12    GTCCAAAAGGAAGAGCATGTGCCAGTCCCACTGGATTGGGGTAGAAATCCTGGCAGCAGG 1140
DuxG13    GTCCAAAAGGAAGAGCATGTGCCAGTCCCACTGGATTGGGGTAGAAATCCTGGCAGCAGG 1140
DuxG14    GTCCAAAAGGAAGAGCATGTGCCAGTCCCACTGGATTGGGGTAGAAATCCTGGCAGCAGG 1140
DuxG15    GTCCAAAAGGAAGAGCATGTGCCAGTCCCACTGGATTGGGGTAGAAATCCTGGCAGCAGG 1140
DuxG16    GTCCAAAAGGAAGAGCATGTGCCAGTCCCACTGGATTGGGGTAGAAATCCTGGCAGCAGG 1140
DuxG17    GTCCAAAAGGAAGAGCATGTGCCAGTCCCACTGGATTGGGGTAGAAATCCTGGCAGCAGG 1140
DuxG18    GTCCAAAAGGAAGAGCATGTGCCAGTCCCACTGGATTGGGGTAGAAATCCTGGCAGCAGG 1140
DuxG19    GTCCAAAAGGAAGAGCATGTGCCAGTCCCACTGGATTGGGGTAGAAATCCTGGCAGCAGG 1140
DuxG20    GTCCAAAAGGAAGAGCATGTGCCAGTCCCACTGGATTGGGGTAGAAATCCTGGCAGCAGG 1140
DuxG21    GTCCAAAAGGAAGAGCATGTGCCAGTCCCACTGGATTGGGGTAGAAATCCTGGCAGCAGG 1140
DuxG22    GTCCAAAAGGAAGAGCATGTGCCAGTCCCACTGGATTGGGGTAGAAATCCTGGCAGCAGG 1140
DuxG23    GTCCAAAAGGAAGAGCATGTGCCAGTCCCACTGGATTGGGGTAGAAATCCTGGCAGCAGG 1140

Dux       GAGCATGAAGGTTCCCAGGACAGCTTACTGCCCCTGGAGGAAGCAGTAAATTCGGGCATG 1200
DuxG1     GAGCATGAAGGTTCCCAGGACAGCTTACTGCCCCTGGAGGAAGCAGTAAATTCGGGCATG 1200
DuxG2     GAGCATGAAGGTTCCCAGGACAGCTTACTGCCCCTGGAGGAAGCAGTAAATTCGGGCATG 1200
DuxG3     GAGCATGAAGGTTCCCAGGACAGCTTACTGCCCCTGGAGGAAGCAGTAAATTCGGGCATG 1200
DuxG4     GAGCATGAAGGTTCCCAGGACAGCTTACTGCCCCTGGAGGAAGCAGTAAATTCGGGCATG 1200
DuxG5     GAGCATGAAGGTTCCCAGGACAGCTTACTGCCCCTGGAGGAAGCAGTAAATTCGGGCATG 1200
DuxG6     GAGCATGAAGGTTCCCAGGACAGCTTACTGCCCCTGGAGGAAGCAGTAAATTCGGGCATG 1200
DuxG7     GAGCATGAAGGTTCCCAGGACAGCTTACTGCCCCTGGAGGAAGCAGTAAATTCGGGCATG 1200
DuxG8     GAGCATGAAGGTTCCCAGGACAGCTTACTGCCCCTGGAGGAAGCAGTAAATTCGGGCATG 1200
DuxG9     GAGCATGAAGATTCCCAGGACAGCTTACTGCCCCTGGAGGAAGCAGTAAATTCGGGCATG 1200
DuxG10    GAGCATGAAGGTTCCCAGGACAGCTTACTGCCCCTGGAGGAAGCAGTAAATTCGGGCATG 1200
DuxG11    GAGCATGAAGGTTCCCAGGACAGCTTACTGCCCCTGGAGGAAGCAGTAAATTCGGGCATG 1200
DuxG12    GAGCATGAAGGTTCCCAGGACAGCTTACTGCCCCTGGAGGAAGCAGTAAATTCGGGCATG 1200
DuxG13    GAGCATGAAGGTTCCCAGGACAGCTTACTGCCCCTGGAGGAAGCAGTAAATTCGGGCATG 1200
DuxG14    GAGCATGAAGGTTCCCAGGACAGCTTACTGCCCCTGGAGGAAGCAGTAAATTCGGGCATG 1200
DuxG15    GAGCATGAAGGTTCCCAGGACAGCTTACTGCCCCTGGAGGAAGCAGTAAATTCGGGCATG 1200
DuxG16    GAGCATGAAGGTTCCCAGGACAGCTTACTGCCCCTGGAGGAAGCAGTAAATTCGGGCATG 1200
DuxG17    GAGCATGAAGGTTCCCAGGACAGCTTACTGCCCCTGGAGGAAGCAGTAAATTCGGGCATG 1200
DuxG18    GAGCATGAAGGTTCCCAGGACAGCTTACTGCCCCTGGAGGAAGCAGTAAATTCGGGCATG 1200
DuxG19    GAGCATGAAGGTTCCCAGGACAGCTTACTGCCCCTGGAGGAAGCAGTAAATTCGGGCATG 1200
DuxG20    GAGCATGAAGGTTCCCAGGACAGCTTACTGCCCCTGGAGGAAGCAGTAAATTCGGGCATG 1200
DuxG21    GAGCATGAAGGTTCCCAGGACAGCTTACTGCCCCTGGAGGAAGCAGTAAATTCGGGCATG 1200
DuxG22    GAGCATGAAGGTTCCCAGGACAGCTTACTGCCCCTGGAGGAAGCAGTAAATTCGGGCATG 1200
DuxG23    GAGCATGAAGGTTCCCAGGACAGCTTACTGCCCCTGGAGGAAGCAGTAAATTCGGGCATG 1200
                    *

Dux       GATACCTCGATCCCTAGCATCTGGCCAACCTTCTGCAGAGAATCCCAGCCTCCCCAAGTG 1260
DuxG1     GATACCTCGATCCCTAGCATCTGGCCAACCTTCTGCAGAGAATCCCAGCCTCCCCAAGTG 1260
DuxG2     GATACCTCGATCCCTAGCATCTGGCCAACCTTCTGCAGAGAATCCCAGCCTCCCCAAGTG 1260
DuxG3     GATACCTCGATCCCTAGCATCTGGCCAACCTTCTGCAGAGAATCCCAGCCTCCCCAAGTG 1260
DuxG4     GATACCTCGATCCCTAGCATCTGGCCAACCTTCTGCAGAGAATCCCAGCCTCCCCAAGTG 1260
DuxG5     GATACCTCGATCCCTAGCATCTGGCCAACCTTCTGCAGAGAATCCCAGCCTCCCCAAGTG 1260
DuxG6     GATACCTCGATCCCTAGCATCTGGCCAACCTTCTGCAGAGAATCCCAGCCTCCCCAAGTG 1260
DuxG7     GATACCTCGATCCCTAGCATCTGGCCAACCTTCTGCAGAGAATCCCAGCCTCCCCAAGTG 1260
DuxG8     GATACCTCGATCCCTAGCATCTGGCCAACCTTCTGCAGAGAATCCCAGCCTCCCCAAGTG 1260
DuxG9     GATACCTCGATCCCTAGCATCTGGCCAACCTTCTGCAGAGAATCCCAGCCTCCCCAAGTG 1260
DuxG10    GATACCTCGATCCCTAGCATCTGGCCAACCTTCTGCAGAGAATCCCAGCCTCCCCAAGTG 1260
DuxG11    GATACCTCGATCCCTAGCATCTGGCCAACCTTCTGCAGAGAATCCCAGCCTCCCCAAGTG 1260
DuxG12    GATACCTCGATCCCTAGCATCTGGCCAACCTTCTGCAGAGAATCCCAGCCTCCCCAAGTG 1260
DuxG13    GATACCTCGATCCCTAGCATCTGGCCAACCTTCTGCAGAGAATCCCAGCCTCCCCAAGTG 1260
DuxG14    GATACCTCGATCCCTAGCATCTGGCCAACCTTCTGCAGAGAATCCCAGCCTCCCCAAGTG 1260
DuxG15    GATACCTCGATCCCTAGCATCTGGCCAACCTTCTGCAGAGAATCCCAGCCTCCCCAAGTG 1260
DuxG16    GATACCTCGATCCCTAGCATCTGGCCAACCTTCTGCAGAGAATCCCAGCCTCCCCAAGTG 1260
DuxG17    GATACCTCGATCCCTAGCATCTGGCCAACCTTCTGCAGAGAATCCCAGCCTCCCCAAGTG 1260
DuxG18    GATACCTCGATCCCTAGCATCTGGCCAACCTTCTGCAGAGAATCCCAGCCTCCCCAAGTG 1260
DuxG19    GATACCTCGATCCCTAGCATCTGGCCAACCTTCTGCAGAGAATCCCAGCCTCCCCAAGTG 1260
DuxG20    GATACCTCGATCCCTAGCATCTGGCCAACCTTCTGCAGAGAATCCCAGCCTCCCCAAGTG 1260
DuxG21    GATACCTCGATCCCTAGCATCTGGCCAACCTTCTGCAGAGAATCCCAGCCTCCCCAAGTG 1260
DuxG22    GATACCTCGATCCCTAGCATCTGGCCAACCTTCTGCAGAGAATCCCAGCCTCCCCAAGTG 1260
DuxG23    GATACCTCGATCCCTAGCATCTGGCCAACCTTCTGCAGAGAATCCCAGCCTCCCCAAGTG 1260
```

Dux GCACAGCCCTCTGGACCAGGCCAAGCACAGGCCCCCACTCAAGGTGGGAACACGGACCCC 1320
DuxG1 GCACAGCCCTCTGGACCAGGCCAAGCACAGGCCCCCACTCAAGGTGGGAACACGGACCCC 1320
DuxG2 GCACAGCCCTCTGGACCAGGCCAAGCACAGGCCCCCACTCAAGGTGGGAACACGGACCCC 1320
DuxG3 GCACAGCCCTCTGGACCAGGCCAAGCAC**C**GGCCCCCACTCAAGGTGGGAACACGGACCCC 1320
DuxG4 GCACAGCCCTCTGGACCAGGCCAAGCACAGGCCCCCACTCAAGGTGGGAACACGGACCCC 1320
DuxG5 GCACAGCCCTCTGGACCAGGCCAAGCACAGGCCCCCACTCAAGGTGGGAACACGGACCCC 1320
DuxG6 GCACAGCCCTCTGGACCAGGCCAAGCAC**C**GGCCCCCACTCAAGGTGGGAACACGGACCCC 1320
DuxG7 GCACAGCCCTCTGGACCAGGCCAAGCACAGGCCCCCACTCAAGGTGGGAACACGGACCCC 1320
DuxG8 GCACAGCCCTCTGGACCAGGCCAAGCAC**C**GGCCCCCACTCAAGGTGGGAACACGGACCCC 1320
DuxG9 GCACAGCCCTCTGGACCAGGCCAAGCACAGGCCCCCACTCAAGGTGGGAACACGGACCCC 1320
DuxG10 GCACAGCCCTCTGGACCAGGCCAAGCACAGGCCCCCACTCAAGGTGGGAACACGGACCCC 1320
DuxG11 GCACAGCCCTCTGGACCAGGCCAAGCAC**C**GGCCCCCACTCAAGGTGGGAACACGGACCCC 1320
DuxG12 GCACAGCCCTCTGGACCAGGCCAAGCACAGGCCCCCACTCAAGGTGGGAACACGGACCCC 1320
DuxG13 GCACAGCCCTCTGGACCAGGCCAAGCACAGGCCCCCACTCAAGGTGGGAACACGGACCCC 1320
DuxG14 GCACAGCCCTCTGGACCAGGCCAAGCACAGGCCCCCACTCAAGGTGGGAACACGGACCCC 1320
DuxG15 GCACAGCCCTCTGGACCAGGCCAAGCAC**C**GGCCCCCACTCAAGGTGGGAACACGGACCCC 1320
DuxG16 GCACAGCCCTCTGGACCAGGCCAAGCACAGGCCCCCACTCAAGGTGGGAACACGGACCCC 1320
DuxG17 GCACAGCCCTCTGGACCAGGCCAAGCAC**C**GGCCCCCACTCAAGGTGGGAACACGGACCCC 1320
DuxG18 GCACAGCCCTCTGGACCAGGCCAAGCACAGGCCCCCACTCAAGGTGGGAACACGGACCCC 1320
DuxG19 GCACAGCCCTCTGGACCAGGCCAAGCACAGGCCCCCACTCAAGGTGGGAACACGGACCCC 1320
DuxG20 GCACAGCCCTCTGGACCAGGCCAAGCACAGGCCCCCACTCAAGGTGGGAACACGGACCCC 1320
DuxG21 GCACAGCCCTCTGGACCAGGCCAAGCACAGGCCCCCACTCAAGGTGGGAACACGGACCCC 1320
DuxG22 GCACAGCCCTCTGGACCAGGCCAAGCACAGGCCCCCACTCAAGGTGGGAACACGGACCCC 1320
DuxG23 GCACAGCCCTCTGGACCAGGCCAAGCACAGGCCCCCACTCAAGGTGGGAACACGGACCCC 1320
*

Dux CTGGAGCTCTTCCTCTATCAACTGTTGGATGAAGTCCAAGTAGAAGAGCATGCTCCAGCC 1380
DuxG1 CTGGAGCTCTTCCTCTATCAACTGTTGGATGAAGTCCAAGTAGAAGAGCATGCTCCAGCC 1380
DuxG2 CTGGAGCTCTTCCTCTATCAACTGTTGGATGAAGTCCAAGTAGAAGAGCATGCTCCAGCC 1380
DuxG3 CTGGAGCTCTTCCTCTATCAACTGTTGGATGAAGTCCAAGTAGAAGAGCATGCTCCAGCC 1380
DuxG4 CTGGAGCTCTTCCTCTATCAACTGTTGGATGAAGTCCAAGTAGAAGAGCATGCTCCAGCC 1380
DuxG5 CTGGAGCTCTTCCTCTATCAACTGTTGGATGAAGTCCAAGTAGAAGAGCATGCTCCAGCC 1380
DuxG6 CTGGAGCTCTTCCTCTATCAACTGTTGGATGAAGTCCAAGTAGAAGAGCATGCTCCAGCC 1380
DuxG7 CTGGAGCTCTTCCTCTATCAACTGTTGGATGAAGTCCAAGTAGAAGAGCATGCTCCAGCC 1380
DuxG8 CTGGAGCTCTTCCTCTATCAACTGTTGGATGAAGTCCAAGTAGAAGAGCATGCTCCAGCC 1380
DuxG9 CTGGAGCTCTTCCTCTATCAACTGTTGGATGAAGTCCAAGTAGAAGAGCATGCTCCAGCC 1380
DuxG10 CTGGAGCTCTTCCTCTATCAACTGTTGGATGAAGTCCAAGTAGAAGAGCATGCTCCAGCC 1380
DuxG11 CTGGAGCTCTTCCTCTATCAACTGTTGGATGAAGTCCAAGTAGAAGAGCATGCTCCAGCC 1380
DuxG12 CTGGAGCTCTTCCTCTATCAACTGTTGGATGAAGTCCAAGTAGAAGAGCATGCTCCAGCC 1380
DuxG13 CTGGAGCTCTTCCTCTATCAACTGTTGGATGAAGTCCAAGTAGAAGAGCATGCTCCAGCC 1380
DuxG14 CTGGAGCTCTTCCTCTATCAACTGTTGGATGAAGTCCAAGTAGAAGAGCATGCTCCAGCC 1380
DuxG15 CTGGAGCTCTTCCTCTATCAACTGTTGGATGAAGTCCAAGTAGAAGAGCATGCTCCAGCC 1380
DuxG16 CTGGAGCTCTTCCTCTATCAACTGTTGGATGAAGTCCAAGTAGAAGAGCATGCTCCAGCC 1380
DuxG17 CTGGAGCTCTTCCTCTATCAACTGTTGGATGAAGTCCAAGTAGAAGAGCATGCTCCAGCC 1380
DuxG18 CTGGAGCTCTTCCTCTATCAACTGTTGGATGAAGTCCAAGTAGAAGAGCATGCTCCAGCC 1380
DuxG19 CTGGAGCTCTTCCTCTATCAACTGTTGGATGAAGTCCAAGTAGAAGAGCATGCTCCAGCC 1380
DuxG20 CTGGAGCTCTTCCTCTATCAACTGTTGGATGAAGTCCAAGTAGAAGAGCATGCTCCAGCC 1380
DuxG21 CTGGAGCTCTTCCTCTATCAACTGTTGGATGAAGTCCAAGTAGAAGAGCATGCTCCAGCC 1380
DuxG22 CTGGAGCTCTTCCTCTATCAACTGTTGGATGAAGTCCAAGTAGAAGAGCATGCTCCAGCC 1380
DuxG23 CTGGAGCTCTTCCTCTATCAACTGTTGGATGAAGTCCAAGTAGAAGAGCATGCTCCAGCC 1380

Dux CCTCTGAATTGGGATGTAGATCCTGGTGGCAGGGTGCATGAAGGTTCGTGGGAGAGCTTT 1440
DuxG1 CCTCTGAATTGGGATGTAGATCCTGGTGGCAGGGTGCATGAAGGTTCGTGGGAGAGCTTT 1440
DuxG2 CCTCTGAATTGGGATGTAGATCCTGGTGGCAGGGTGCATGAAGGTTCGTGGGAGAGCTTT 1440
DuxG3 CCTCTGAATTGGGATGTAGATCCTGGTGGCAGGGTGCATGAAGGTTCGTGGGAGAGCTTT 1440
DuxG4 CCTCTGAATTGGGATGTAGATCCTGGTGGCAGGGTGCATGAAGGTTCGTGGGAGAGCTTT 1440
DuxG5 CCTCTGAATTGGGATGTAGATCCTGGTGGCAGGGTGCATGAAGGTTCGTGGGAGAGCTTT 1440
DuxG6 CCTCTGAATTGGGATGTAGATCCTGGTGGCAGGGTGCATGAAGGTTCGTGGGAGAGCTTT 1440
DuxG7 CCTCTGAATTGGGATGTAGATCCTGGTGGCAGGGTGCATGAAGGTTCGTGGGAGAGCTTT 1440
DuxG8 CCTCTGAATTGGGATGTAGATCCTGGTGGCAGGGTGCATGAAGGTT**T**GTGGGAGAGCTTT 1440
DuxG9 CCTCTGAATTGGGATGTAGAT**T**CTGGTGGCAGGGTGCATGAAGGTTCGTGGGAGAGCTTT 1440
DuxG10 CCTCTGAATTGGGATGTAGATCCTGGTGGCAGGGTGCATGAAGGTTCGTGGGAGAGCTTT 1440
DuxG11 CCTCTGAATTGGGATGTAGATCCTGGTGGCAGGGTGCATGAAGGTT**T**GTGGGAGAGCTTT 1440
DuxG12 CCTCTGAATTGGGATGTAGATCCTGGTGGCAGGGTGCATGAAGGTTCGTGGGAGAGCTTT 1440
DuxG13 CCTCTGAATTGGGATGTAGATCCTGGTGGCAGGGTGCATGAAGGTTCGTGGGAGAGCTTT 1440
DuxG14 CCTCTGAATTGGGATGTAGATCCTGGTGGCAGGGTGCATGAAGGTTCGTGGGAGAGCTTT 1440
DuxG15 CCTCTGAATTGGGATGTAGATCCTGGTGGCAGGGTGCATGAAGGTTCGTGGGAGAGCTTT 1440
DuxG16 CCTCTGAATTGGGATGTAGATCCTGGTGGCAGGGTGCATGAAGGTTCGTGGGAGAGCTTT 1440
DuxG17 CCTCTGAATTGGGATGTAGATCCTGGTGGCAGGGTGCATGAAGGTT**T**GTGGGAGAGCTTT 1440
DuxG18 CCTCTGAATTGGGATGTAGATCCTGGTGGCAGGGTGCATGAAGGTTCGTGGGAGAGCTTT 1440
DuxG19 CCTCTGAATTGGGATGTAGATCCTGGTGGCAGGGTGCATGAAGGTTCGTGGGAGAGCTTT 1440
DuxG20 CCTCTGAATTGGGATGTAGATCCTGGTGGCAGGGTGCATGAAGGTTCGTGGGAGAGCTTT 1440
DuxG21 CCTCTGAATTGGGATGTAGATCCTGGTGGCAGGGTGCATGAAGGTTCGTGGGAGAGCTTT 1440
DuxG22 CCTCTGAATTGGGATGTAGATCCTGGTGGCAGGGTGCATGAAGGTTCGTGGGAGAGCTTT 1440
DuxG23 CCTCTGAATTGGGATGTAGATCCTGGTGGCAGGG**G**GCATGAAGGTTCGTGGGAGAGCTTT 1440
* * *

```
Dux     TGGCCACAGGAAGAAGCAGGAAGTACAGGCCTGGATACTTCAAGCCCCAGCGACTCAAAC 1500
DuxG1   TGGCCACAGGAAGAAGCAGGAAGTACAGGCCTGGATACTTCAAGCCCCAGCGACTCAAAC 1500
DuxG2   TGGCCACAGGAAGAAGCAGGAAGTACAGGCCTGGATACTTCAAGCCCCAGCGACTCAAAC 1500
DuxG3   TGGCCACAGGAAGAAGCAGGAAGTACAGGCCTGGATACTTCAAGCCCCAGCGACTCAAAC 1500
DuxG4   TGGCCACAGGAAGAAGCAGGAAGTACAGGCATGGATACTTCTAGCCCCAGCGACTCAAAC 1500
DuxG5   TGGCCACAGGAAGAAGCAGGAAGTACAGGCCTGGATACTTCAAGCCCCAGCGACTCAAAC 1500
DuxG6   TGGCCACAGGAAGAAGCAGGAAGTACAGGCCTGGATACTTCAAGCCCCAGCGACTCAAAC 1500
DuxG7   TGGCCACAGGAAGAAGCAGGAAGTACAGGCCTGGATACTTCAAGCCCCAGCGACTCAAAC 1500
DuxG8   TGGCCACAGGAAGAAGCAGGAAGTACAGGCCTGGATACTTCAAGCCCCAGCGACTCAAAC 1500
DuxG9   TGGCCACAGGAAGAAGCAGGAAGTACAGGCCTGGATACTTCAAGCCCCAGCGACTCAAAC 1500
DuxG10  TGGCCACAGGAAGAAGCAGGAAGTACAGGCCTGGATACTTCAAGCCCCAGCGACTCAAAC 1500
DuxG11  TGGCCACAGGAAGAAGCAGGAAGTACAGGCCTGGATACTTCAAGCCCCAGCGACTCAAAC 1500
DuxG12  TGGCCACAGGAAGAAGCAGGAAGTACAGGCCTGGATACTTCAAGCCCCAGCGACTCAAAC 1500
DuxG13  TGGCCACAGGAAGAAGCAGGAAGTACAGGCCTGGATACTTCAAGCCCCAGCGACTCAAAC 1500
DuxG14  TGGCCACAGGAAGAAGCAGGAAGTACAGGCCTGGATACTTCAAGCCCCAGCGACTCAAAC 1500
DuxG15  AGGCCACAGGAAGAAGCAGGAAGTACAGGCCTGGATACTTCAAGCCCCAGCGACTCAAAC 1500
DuxG16  TGGCCACAGGAAGAAGCAGGAAGTACAGGCCTGGATACTTCAAGCCCCAGCGACTCAAAC 1500
DuxG17  TGGCCACAGGAAGAAGCAGGAAGTACAGGCCTGGATACTTCAAGCCCCAGCGACTCAAAC 1500
DuxG18  TGGCCACAGGAAGAAGCAGGAAGTACAGGCCTGGATACTTCAAGCCCCAGCGACTCAAAC 1500
DuxG19  TGGCCACAGGAAGAAGCAGGAAGTACAGGCCTGGATACTTCAAGCCCCAGCGACTCAGAC 1500
DuxG20  TGGCCACAGGAAGAAGCAGGAAGTACAGGCCTGGATACTTCAAGCCCCAGCGACTCAAAC 1500
DuxG21  TGGCCACAGGAAGAAGCAGGAAGTACAGGCCTGGATACTTCAAGCCCCAGCGACTCAGAC 1500
DuxG22  TGGCCACAGGAAGAAGCAGGAAGTACAGGCCTGGATACTTCAAGCCCCAGCGACTCAAAC 1500
DuxG23  TGGCCACAGGAAGAAGCAGGAAGTACAGGCCTGGATACTTCAAGCCCCAGCGACTCAAAC 1500
        *                             *          *               *

Dux     TCCTTCTTCAGAGAGTCCAAGCCTTCCCAAGTGGCACAGCGCCGTGGAGCGGGCCAAGAA 1560
DuxG1   TCCTTCTTCAGAGAGTCCAAGCCTTCCCAAGTGGCACAGCGCCGTGGAGCGGGCCAAGAA 1560
DuxG2   TCCTTCTTCAGAGAGTCCAAGCCTTCCCAAGTGGCACAGCGCCGTGGAGCGGGCCAAGAA 1560
DuxG3   TCCTTCTTCAGAGAGTCCAAGCCTTCCCAAGTGGCACAGCGCCGTGGAGCGGGCCAAGAA 1560
DuxG4   TCCTTCTTCAGAGAGTCCAAGCCTTCCCAAGTGGCACAGCGCCGTGGAGCGGGCCAAGAA 1560
DuxG5   TCCTTCTTCAGAGAGTCCAAGCCTTCCCAAGTGGCACAGCGCCGTGGAGCGGGCCAAGAA 1560
DuxG6   TCCTTCTTCAGAGAGTCCAAGCCTTCCCAAGTGGCACAGCGCCGTGGAGCGGGCCAAGAA 1560
DuxG7   TCCTTCTTCAGAGAGTCCAAGCCTTCCCAAGTGGCACAGCGCCGTGGAGCGGGCCAAGAA 1560
DuxG8   TCCTTCTTCAGAGAGTCCAAGCCTTCCCAAGTGGCACAGCGCCGTGGAGCGGGCCAAGAA 1560
DuxG9   TCCTTCTTCAGAGAGTCCAAGCCTTCCCAAGTGGCACAGCGCCGTGGAGCGGGCCAAGAA 1560
DuxG10  TCCTTCTTCAGAGAGTCCAAGCCTTCCCAAGTGGCACAGCGCCGTGGAGCGGGCCAAGAA 1560
DuxG11  TCCTTCTTCAGAGAGTCCAAGCCTTCCCAAGTGGCACAGCGCCGTGGAGCGGGCCAAGAA 1560
DuxG12  TCCTTCTTCAGAGAGTCCAAGCCTTCCCAAGTGGCACAGCGCCGTGGAGCGGGCCAAGAA 1560
DuxG13  TCCTTCTTCAGAGAGTCCAAGCCTTCCCAAGTGGCACAGCGCCGTGGAGCGGGCCAAGAA 1560
DuxG14  TCCTTCTTCAGAGAGTCCAAGCCTTCCCAAGTGGCACAGCGCCGTGGAGCGGGCCAAGAA 1560
DuxG15  TCCTTCTTCAGAGAGTCCAAGCCTTCCCAAGTGGCACAGCGCCGTGGAGCGGGCCAAGAA 1560
DuxG16  TCCTTCTTCAGAGAGTCCAAGCCTTCCCAAGTGGCACAGCGCCGTGGAGCGGGCCAAGAA 1560
DuxG17  TCCTTCTTCAGAGAGTCCAAGCCTTCCCAAGTGGCACAGCGCCGTGGAGCGGGCCAAGAA 1560
DuxG18  TCCTTCTTCAGAGAGTCCAAGCCTTCCCAAGTGGCACAGCGCCGTGGAGCGGGCCAAGAA 1560
DuxG19  TCCTTCTGCAGAGAGTCCAAGCCTTCCCAAGTGGCACAGCGCCGTGGAGCGGGCCAAGAA 1560
DuxG20  TCCTTCTTCAGAGAGTCCAAGCCTTCCCAAGTGGCACAGCGCCGTGGAGCGGGCCAAGAA 1560
DuxG21  TCCTTCTTCAGAGAGTCCAAGCCTTCCCAAGTGGCACAGCGCCGTGGAGCGGGCCAAGAA 1560
DuxG22  TCCTTCTTCAGAGAGTCCAAGCCTTCCCAAGTGGCACAGCGCCGTGGAGCGGGCCAAGAA 1560
DuxG23  TCCTTCTGCAGAGAGTCCAAGCCTTCCCAAGTGGCACAGCGCCGTGGAGCGGGCCAAGAA 1560

Dux     GATGCCCGCACTCAAGCAGACAGCACAGGCCCTCTGGAACTCCTCCTCTTTGATCAACTG 1620
DuxG1   GATGCCCGCACTCAAGCAGACAGCACAGGCCCTCTGGAACTCCTCCTCTTTGATCAACTG 1620
DuxG2   GATGCCCGCACTCAAGCAGACAGCACAGGCCCTCTGGAACTCCTCCTCTTTGATCAACTG 1620
DuxG3   GATGCCCGCACTCAAGCAGACAGCACAGGCCCTCTGGAACTCCTCCTCTTTGATCAACTG 1620
DuxG4   GATGCCCGCACTCAAGCAGACAGCACAGGCCCTCTGGAACTCCTCCTCTTTGATCAACTG 1620
DuxG5   GATGCCCGCACTCAAGCAGACAGCACAGGCCCTCTGGAACTCCTCCTCTTTGATCAACTG 1620
DuxG6   GATGCCCGCACTCAAGCAGACAGCACAGGCCCTCTGGAACTCCTCCTCTTTGATCAACTG 1620
DuxG7   GATGCCCGCACTCAAGCAGACAGCACAGGCCCTCTGGAACTCCTCCTCTTTGATCAACTG 1620
DuxG8   GATGCCCGCACTCAAGCAGACAGCACAGGCCCTCTGGAACTCCTCCTCTTTGATCAACTG 1620
DuxG9   GATGCCCGCACTCAAGCAGACAGCACAGGCCCTCTGGAACTCCTCCTCTTTGATCAACTG 1620
DuxG10  GATGCCCGCACTCAAGCAGACAGCACAGGCCCTCTGGAACTCCTCCTCTTTGATCAACTG 1620
DuxG11  GATGCCCGCACTCAAGCAGACAGCACAGGCCCTCTGGAACTCCTCCTCTTTGATCAACTG 1620
DuxG12  GATGCCCGCACTCAAGCAGACAGCACAGGCCCTCTGGAACTCCTCCTCTTTGATCAACTG 1620
DuxG13  GATGCCCGCACTCAAGCAGACAGCACAGGCCCTCTGGAACTCCTCCTCCTTGATCAACTG 1620
DuxG14  GATGCCCGCACTCAAGCAGACAGCACAGGCCCTCTGGAACTCCTCCTCTTTGATCAACTG 1620
DuxG15  GATGCCCGCACTCAAGCAGACAGCACAGGCCCTCTGGAACTCCTCCTCTTTGATCAACTG 1620
DuxG16  GATGCCCGCACTCAAGCAGACAGCACAGGCCCTCTGGAACTCCTCCTCTTTGATCAACTG 1620
DuxG17  GATGCCCGCACTCAAGCAGACAGCACAGGCCCTCTGGAACTCCTCCTCTTTGATCAACTG 1620
DuxG18  GATGCCCGCACTCAAGCAGACAGCACAGGCCCTCTGGAACTCCTCCTCTTTGATCAACTG 1620
DuxG19  GATGCCCGCACTCAAGCAGACAGCACAGGCCCTCTGGAACTCCTCCTCCTTGATCAACTG 1620
DuxG20  GATGCCCGCACTCAAGCAGACAGCACAGGCCCTCTGGAACTCCTCCTCTTTGATCAACTG 1620
DuxG21  GATGCCCGCACTCAAGCAGACAGCACAGGCCCTCTGGAACTCCTCCTCTTTGATCAACTG 1620
DuxG22  GATGCCCGCACTCAAGCAGACAGCACAGGCCCTCTGGAACTCCTCCTCTTTGATCAACTG 1620
DuxG23  GATGCCCGCACTCAAGCAGACAGCACAGGCCCTCTGGAACTCCTCCTCTTTGATCAACTG 1620
                                                        *
```

Dux CTGGACGAAGTCCAAAAGGAAGAGCATGTGCCAGCCCCACTGGATTGGGGTAGAAATCCT 1680
DuxG1 CTGGACGAAGTCCAAAAGGAAGAGCATGTGCCAGCCCCACTGGATTGGGGTAGAAATCCT 1680
DuxG2 CTGGACGAAGTCCAAAAGGAAGAGCATGTGCCAGCCCCACTGGATTGGGGTAGAAATCCT 1680
DuxG3 CTGGACGAAGTCCAAAAGGAAGAGCATGTGCCAGCCCCACTGGATTGGGGTAGAAATCCT 1680
DuxG4 CTGGACGAAGTCCAAAAGGAAGAGCATGTGCCAGCCCCACTGGATTGGGGTAGAAATCCT 1680
DuxG5 CTGGACGAAGTCCAAAAGGAAGAGCATGTGCCAGCCCCACTGGATTGGGGTAGAAATCCT 1680
DuxG6 CTGGACGAAGTCCAAAAGGAAGAGCATGTGCCAGCCCCACTGGATTGGGGTAGAAATCCT 1680
DuxG7 CTGGACGAAGTCCAAAAGGAAGAGCATGTGCCAGCCCCACTGGATTGGGGTAGAAATCCT 1680
DuxG8 CTGGACGAAGTCCAAAAGGAAGAGCATGTGCCAGCCCCACTGGATTGGGGTAGAAATCCT 1680
DuxG9 CTGGACGAAGTCCAAAAGGAAGAGCATGTGCCAGCCCCACTGGATTGGGGTAGAAATCCT 1680
DuxG10 CTGGACGAAGTCCAAAAGGAAGAGCATGTGCCAGCCCCACTGGATTGGGGTAGAAATCCT 1680
DuxG11 CTGGACGAAGTCCAAAAGGAAGAGCATGTGCCAGCCCCACTGGATTGGGGTAGAAATCCT 1680
DuxG12 CTGGACGAAGTCCAAAAGGAAGAGCATGTGCCAGCCCCACTGGATTGGGGTAGAAATCCT 1680
DuxG13 CTGGAC**A**AAGTCCAAAAGGAAGAGCATGTGCCAGCCCCACTGGATTGGGGTAGAAATCCT 1680
DuxG14 CTGGACGAAGTCCAAAAGGAAGAGCATGTGCCAGCCCCACTGGATTGGGGTAGAAATCCT 1680
DuxG15 CTGGACGAAGTCCAAAAGGAAGAGCATGTGCCAGCCCCACTGGATTGGGGTAGAAATCCT 1680
DuxG16 CTGGACGAAGTCCAAAAGGAAGAGCATGTGCCAGCCCCACTGGATTGGGGTAGAAATCCT 1680
DuxG17 CTGGACGAAGTCCAAAAGGAAGAGCATGTGCCAGCCCCACTGGATTGGGGTAGAAATCCT 1680
DuxG18 CTGGACGAAGTCCAAAAGGAAGAGCATGTGCCAGCCCCACTGGATTGGGGTAGAAATCCT 1680
DuxG19 CTGGACGAAGTCCAAAAGGAAGAGCATGTGCCAGCCCCACTGGATTGGGGTAGAAATCCT 1680
DuxG20 CTGGACGAAGTCCAAAAGGAAGAGCATGTGCCAGCCCCACTGGATTGGGGTAGAAATCCT 1680
DuxG21 CTGGACGAAGTCCAAAAGGAAGAGCATGTGCCAGCCCCACTGGATTGGGGTAGAAATCCT 1680
DuxG22 CTGGACGAAGTCCAAAAGGAAGAGCATGTGCCAGCCCCACTGGATTGGGGTAGAAATCCT 1680
DuxG23 CTGGACGAAGTCCAAAAGGAAGAGCATGTGCCAGCCCCACTGGATTGGGGTAGAAATCCT 1680
      *

Dux GGCAGCATGGAGCATGAAGGTTCCCAGGACAGCTTACTGCCCCTGGAGGAAGCAGCAAAT 1740
DuxG1 GGCAGCATGGAGCATGAAGGTTCCCAGGACAGCTTACTGCCCCTGGAGGAAGCAGCAAAT 1740
DuxG2 GGCAGCATGGAGCATGAAGGTTCCCAGGACAGCTTACTGCCCCTGGAGGAAGCAGCAAAT 1740
DuxG3 GGCAGCATGGAGCATGAAGGTTCCCAGGACAGCTTACTGCCCCTGGAGGAAGCAGCAAAT 1740
DuxG4 GGCAGCATGGAGCATGAAGGTTCCCAGGACAGCTTACTGCCCCTGGAGGAAGCAGCAAAT 1740
DuxG5 GGCAGCATGGAGCATGAAGGTTCCCAGGACAGCTTACTGCCCCTGGAGGAAGCAGCAAAT 1740
DuxG6 GGCAGCATGGAGCATGAAGGTTCCCAGGACAGCTTACTGCCCCTGGAGGAAGCAGCAAAT 1740
DuxG7 GGCAGCATGGAGCATGAAGGTTCCCAGGACAGCTTACTGCCCCTGGAGGAAGCAGCAAAT 1740
DuxG8 GGCAGCATGGAGCATGAAGGTTCCCAGGACAGCTTACTGCCCCTGGAGGAAGCAGCAAAT 1740
DuxG9 GGCAGCATGGAGCATGAAGGTTCCCAGGACAGCTTACTGCCCCTGGAGGAAGCAGCAAAT 1740
DuxG10 GGCAGCATGGAGCATGAAGGTTCCCAGGACAGCTTACTGCCCCTGGAGGAAGCAGCAAAT 1740
DuxG11 GGCAGCATGGAGCATGAAGGTTCCCAGGACAGCTTACTGCCCCTGGAGGAAGCAGCAAAT 1740
DuxG12 GGCAGCATGGAGCATGAAGGTTCCCAGGACAGCTTACTGCCCCTGGAGGAAGCAGCAAAT 1740
DuxG13 GGCAGCATGGAGCATGAAGGTTCCCAGGACAGCTTACTGCCCCTGGAGGAAGCAGCAAAT 1740
DuxG14 GGCAGCA**G**GGAGCATGAAGGTTCCCAGGACAGCTTACTGCCCCTGGAGGAAGCAGCAAAT 1740
DuxG15 GGCAGCATGGAGCATGAAGGTTCCCAGGACAGCTTACTGCCCCTGGAGGAAGCAGCAAAT 1740
DuxG16 GGCAGCATGGAGCATGAAGGTTCCCAGGACAGCTTACTGCCCCTGGAGGAAGCAGCAAAT 1740
DuxG17 GGCAGCATGGAGCATGAAGGTTCCCAGGACAGCTTACTGCCCCTGGAGGAAGCAGCAAAT 1740
DuxG18 GGCAGCATGGAGCATGAAGGTTCCCAGGACAGCTTACTGCCCCTGGAGGAAGCAGCAAAT 1740
DuxG19 GGCAGCATGGAGCATGAAGGTTCCCAGGACAGCTTACTGCCCCTGGAGGAAGCAGCAAAT 1740
DuxG20 GGCAGCATGGAGCATGAAGGTTCCCAGGACAGCTTACTGCCCCTGGAGGAAGCAGCAAAT 1740
DuxG21 GGCAGCATGGAGCATGAAGGTTCCCAGGACAGCTTACTGCCCCTGGAGGAAGCAGCAAAT 1740
DuxG22 GGCAGCATGGAGCATGAAGGTTCCCAGGACAGCTTACTGCCCCTGGAGGAAGCAGCAAAT 1740
DuxG23 GGCAGCATGGAGCATGAAGGTTCCCAGGACAGCTTACTGCCCCTGGAGGAAGCAGCAAAT 1740
       *

Dux TCGGGCAGGGATACCTCGATCCCTAGCATCTGGCCAGCCTTCTGCAGAAAATCCCAGCCT 1800
DuxG1 TCGGGCAGGGATACCTCGATCCCTAGCATCTGGCCAGCCTTCTGCAGAAAATCCCAGCCT 1800
DuxG2 TCGGGCAGGGATACCTCGATCCCTAGCATCTGGCCAGCCTTCTGCAGAAAATCCCAGCCT 1800
DuxG3 TCGGGCAGGGATACCTCGATCCCTAGCATCTGGCCAGCCTTCTGCAGAAAATCCCAGCCT 1800
DuxG4 TCGGGCAGGGATACCTCGATCCCTAGCATCTGGCCAGCCTTCTGCAGAAAATCCCAGCCT 1800
DuxG5 TCGGGCAGGGATACCTCGATCCCTAGCATCTGGCCAGCCTTCTGCAGAAAATCCCAGCCT 1800
DuxG6 TCGGGCAGGGATACCTCGATCCCTAGCATCTGGCCAGCCTTCTGCAGAAAATCCCAGCCT 1800
DuxG7 TCGGGCAGGGATACCTCGATCCCTAGCATCTGGCCAGCCTTCTGCAGAAAATCCCAGCCT 1800
DuxG8 TCGGGCAGGGATACCTCGATCCCTAGCATCTGGCCAGCCTTCTGCAGAAAATCCCAGCCT 1800
DuxG9 TCGGGCAGGGATACCTCGATCCCTAGCATCTGGCCAGCCTTCTGCAGAAAATCCCAGCCT 1800
DuxG10 TCGGGCAGGGATACCTCGATCCCTAGCATCTGGCCAGCCTTCTGCAGAAAATCCCAGCCT 1800
DuxG11 TCGGGCAGGGATACCTCGATCCCTAGCATCTGGCCAGCCTTCTGCAGAAAATCCCAGCCT 1800
DuxG12 TCGGGCAGGGATACCTCGATCCCTAGCATCTGGCCAGCCTTCTGCAGAAAATCCCAGCCT 1800
DuxG13 TCGGGCAGGGATACCTCGATCCCTAGCATCTGGCCAGCCTTCTGCAGAAAATCCCAGCCT 1800
DuxG14 TCGGGCA**T**GGATACCTCGATCCCTAGCATCTGGCCA**A**CCTTCTGCAGA**G**AATCCCAGCCT 1800
DuxG15 TCGGGCAGGGATACCTCGATCCCTAGCATCTGGCCAGCCTTCTGCAGAAAATCCCAGCCT 1800
DuxG16 TCGGGCAGGGATACCTCGATCCCTAGCATCTGGCCAGCCTTCTGCAGAAAATCCCAGCCT 1800
DuxG17 TCGGGCAGGGATACCTCGATCCCTAGCATCTGGCCAGCCTTCTGCAGAAAATCCCAGCCT 1800
DuxG18 TCGGGCAGGGATACCTCGATCCCTAGCATCTGGCCAGCCTTCTGCAGAAAATCCCAGCCT 1800
DuxG19 TCGGGCAGGGATACCTCGATCCCTAGCATCTGGCCAGCCTTCTGCAGAAAATCCCAGCCT 1800
DuxG20 TCGGGCAGGGATACCTCGATCCCTAGCATCTGGCCAGCCTTCTGCAGAAAATCCCAGCCT 1800
DuxG21 TCGGGCAGGGATACCTCGATCCCTAGCATCTGGCCAGCCTTCTGCAGAAAATCCCAGCCT 1800
DuxG22 TCGGGCAGGGATACCTCGATCCCTAGCATCTGGCCAGCCTTCTGCAGAAAATCCCAGCCT 1800
DuxG23 TCGGGCAGGGATACCTCGATCCCTAGCATCTGGCCAGCCTTCTGCAGAAAATCCCAGCCT 1800
       *   *   *

```
Dux      CCCCAAGTGGCACAGCCCTCTGGACCAGGCCAAGCACAGGCCCCCATTCAAGGTGGGAAC 1860
DuxG1    CCCCAAGTGGCACAGCCCTCTGGACCAGGCCAAGCACAGGCCCCCATTCAAGGTGGGAAC 1860
DuxG2    CCCCAAGTGGCACAGCCCTCTGGACCAGGCCAAGCACAGGCCCCCATTCAAGGTGGGAAC 1860
DuxG3    CCCCAAGTGGCACAGCCCTCTGGACCAGGCCAAGCACAGGCCCCCATTCAAGGTGGGAAC 1860
DuxG4    CCCCAAGTGGCACAGCCCTCTGGACCAGGCCAAGCACAGGCCCCCATTCAAGGTGGGAAC 1860
DuxG5    CCCCAAGTGGCACAGCCCTCTGGACCAGGCCAAGCACAGGCCCCCATTCAAGGTGGGAAC 1860
DuxG6    CCCCAAGTGGCACAGCCCTCTGGACCAGGCCAAGCACAGGCCCCCATTCAAGGTGGGAAC 1860
DuxG7    CCCCAAGTGGCACAGCCCTCTGGACCAGGCCAAGCACAGGCCCCCATTCAAGGTGGGAAC 1860
DuxG8    CCCCAAGTGGCACAGCCCTCTGGACCAGGCCAAGCACAGGCCCCCATTCAAGGTGGGAAC 1860
DuxG9    CCCCAAGTGGCACAGCCCTCTGGACCAGGCCAAGCACAGGCCCCCATTCAAGGTGGGAAC 1860
DuxG10   CCCCAAGTGGCACAGCCCTCTGGACCAGGCCAAGCACAGGCCCCCATTCAAGGTGGGAAC 1860
DuxG11   CCCCAAGTGGCACAGCCCTCTGGACCAGGCCAAGCACAGGCCCCCATTCAAGGTGGGAAC 1860
DuxG12   CCCCAAGTGGCACAGCCCTCTGGACCAGGCCAAGCACAGGCCCCCATTCAAGGTGGGAAC 1860
DuxG13   CCCCAAGTGGCACAGCCCTCTGGACCAGGCCAAGCACAGGCCCCCATTCAAGGTGGGAAC 1860
DuxG14   CCCCAAGTGGCACAGCCCTCTGGACCAGGCAAAGCACAGGCCCCGACTCAAGGTGGGAAC 1860
DuxG15   CCCCAAGTGGCACAGCCCTCTGGACCAGGCCAAGCACAGGCCCCCATTCAAGGTGGGAAC 1860
DuxG16   CCCCAAGTGGCACAGCCCTCTGGACCAGGCCAAGCACAGGCCCCCATTCAAGGTGGGAAC 1860
DuxG17   CCCCAAGTGGCACAGCCCTCTGGACCAGGCCAAGCACAGGCCCCCATTCAAGGTGGGAAC 1860
DuxG18   CCCCAAGTGGCACAGCCCTCTGGACCAGGCCAAGCACAGGCCCCCATTCAAGGTGGGAAC 1860
DuxG19   CCCCAAGTGGCACAGCCCTCTGGACCAGGCCAAGCACAGGCCCCCATTCAAGGTGGGAAC 1860
DuxG20   CCCCAAGTGGCACAGCCCTCTGGACCAGGCCAAGCACAGGCCCCCATTCAAGGTGGGAAC 1860
DuxG21   CCCCAAGTGGCACAGCCCTCTGGACCAGGCCAAGCACAGGCCCCCATTCAAGGTGGGAAC 1860
DuxG22   CCCCAAGTGGCACAGCCCTCTGGACCAGGCCAAGCACAGGCCCCCATTCAAGGTGGGAAC 1860
DuxG23   CCCCAAGTGGCACAGCCCTCTGGACCAGGCCAAGCACAGGCCCCCATTCAAGGTGGGAAC 1860
                                       *             * *

Dux      ACGGACCCCCTGGAGCTCTTCCTTGATCAACTGCTGACCGAAGTCCAACTTGAGGAGCAG 1920
DuxG1    ACGGACCCCCTGGAGCTCTTCCTTGATCAACTGCTGACCGAAGTCCAACTTGAGGAGCAG 1920
DuxG2    ACGGACCCCCTGGAGCTCTTCCTTGATCAACTGCTGACCGAAGTCCAACTTGAGGAGCAG 1920
DuxG3    ACGGACCCCCTGGAGCTCTTCCTTGATCAACTGCTGACCGAAGTCCAACTTGAGGAGCAG 1920
DuxG4    ACGGACCCCCTGGAGCTCTTCCTTGATCAACTGCTGACCGAAGTCCAACTTGAGGAGCAG 1920
DuxG5    ACGGACCCCCTGGAGCTCTTCCTTGATCAACTGCTGACCGAAGTCCAACTTGAGGAGCAG 1920
DuxG6    ACGGACCCCCTGGAGCTCTTCCTTGATCAACTGCTGACCGAAGTCCAACTTGAGGAGCAG 1920
DuxG7    ACGGACCCCCTGGAGCTCTTCCTTGATCAACTGCTGACCGAAGTCCAACTTGAGGAGCAG 1920
DuxG8    ACGGACCCCCTGGAGCTCTTCCTTGATCAATTGCTGACCGAAGTCCAACTTGAGGAGCAG 1920
DuxG9    ACGGACCCCCTGGAGCTCTTCCTTGATCAACTGCTGACCGAAGTCCAACTTGAGGAGCAG 1920
DuxG10   ACGGACCCCCTGGAGCTCTTCCTTGATCAACTGCTGACCGAAGTCCAACTTGAGGAGCAG 1920
DuxG11   ACGGACCCCCTGGAGCTCTTCCTTGATCAACTGCTGACCGAAGTCCAACTTGAGGAGCAG 1920
DuxG12   ACGGACCCCCTGGAGCTCTTCCTTGATCAACTGCTGACCGAAGTCCAACTTGAGGAGCAG 1920
DuxG13   ACGGACCCCCTGGAGCTCTTCCTTGATCAACTGCTGACCGAAGTCCAACTTGAGGAGCAG 1920
DuxG14   ACGGACCCTCTGGAGCTCTTCCTTGATCAACTGCTGACCGAAGTCCAACTTGAGGAGCAG 1920
DuxG15   ACGGACCCCCTGGAGCTCTTCCTTGATCAACTGCTGACCGAAGTCCAACTTGAGGAGCAG 1920
DuxG16   ACGGACCCCCTGGAGCTCTTCCTTGATCAACTGCTGACCGAAGTCCAACTTGAGGAGCAG 1920
DuxG17   ACGGACCCCCTGGAGCTCTTCCTTGATCAACTGCTGACCGAAGTCCAACTTGAGGAGCAG 1920
DuxG18   ACGGACCCCCTGGAGCTCTTCCTTGATCAACTGCTGACCGAAGTCCAACTTGAGGAGCAG 1920
DuxG19   ACGGACCCCCTGGAGCTCTTCCTTGATCAACTGCTGACCGAAGTCCAACTTGAGGAGCAG 1920
DuxG20   ACGGACCCCCTGGAGCTCTTCCTTGATCAACTGCTGACCGAAGTCCAACTTGAGGAGCAG 1920
DuxG21   ACGGACCCCCTGGAGCTCTTCCTTGATCAACTGCTGACCGAAGTCCAACTTGAGGAGCAG 1920
DuxG22   ACGGACCCCCTGGAGCTCTTCCTTGATCAACTGCTGACCGAAGTCCAACTTGAGGAGCAG 1920
DuxG23   ACGGACCCCCTGGAGCTCTTCCTTGATCAACTGCTGACCGAAGTCCAACTTGAGGAGCAG 1920
                 *                     *

Dux      GGGCCTGCCCCTGTGAATGTGGAGGAAACATGGGAGCAAATGGACACAACACCTGATCTG 1980
DuxG1    GGGCCTGCCCCTGTGAATGTGGAGGAAACATGGGAGCAAATGGACACAACACCTGATCTG 1980
DuxG2    GGGCCTGCCCCTGTGAATGTGGAGGAAACATGGGAGCAAATGGACACAACACCTGATCTG 1980
DuxG3    GGGCCTGCCCCTGTGAATGTGGAGGAAACATGGGAGCAAATGGACACAACACCTGATCTG 1980
DuxG4    GGGCCTGCCCCTGTGAATGTGGAGGAAACATGGGAGCAAATGGACACAACACCTGATCTG 1980
DuxG5    GGGCCTGCCCCTGTGAATGTGGAGGAAACATGGGAGCAAATGGACACAACACCTGATCTG 1980
DuxG6    GGGCCTGCCCCTGTGAATGTGGAGGAAACATGGGAGCAAATGGACACAACACCTGATCTG 1980
DuxG7    GGGCCTGCCCCTGTGAATGTGGAGGAAACATGGGAGCAAATGGACACAACACCTGATCTG 1980
DuxG8    GGGCCTGCCCCTGTGAATGTGGAGGAAACATGGGAGCAAATGGACACAACACCTGATCTG 1980
DuxG9    GGGCCTGCCCCTGTGAATGTGGAGGAAACATGGGAGCAAATGGACACAACACCTGATCTG 1980
DuxG10   GGGCCTGCCCCTGTGAATGTGGAGGAAACATGGGAGCAAATGGACACAACACCTGATCTG 1980
DuxG11   GGGCCTGCCCCTGTGAATGTGGAGGAAACATGGGAGCAAATGGACACAACACCTGATCTG 1980
DuxG12   GGGCCTGCCCCTGTGAATGTGGAGGAAACATGGGAGCAAATGGACACAACACCTGATCTG 1980
DuxG13   GGGCCTGCCCCTGTGAATGTGGAGGAAACATGGGAGCAAATGGACACAACACCTGATCTG 1980
DuxG14   GGGCCTTCCCCTGTGAATGTGGAGGAAACATGGGAGCAAATGGACACAACACCTGATCTG 1980
DuxG15   GGGCCTGCCCCTGTGAATGTGGAGGAAACATGGGAGCAAATGGACACAACACCTGATCTG 1980
DuxG16   GGGCCGGCCCCTGTGAATGTGGAGGAAACATGGGAGCAAATGGACACAACACCTGATCTG 1980
DuxG17   GGGCCTGCCCCTGTGAATGTGGAGGAAACATGGGAGCAAATGGACACAACACCTGATCTG 1980
DuxG18   GGGCCTGCCCCTGTGAATGTGGAGGAAACATGGGAGCAAATGGACACAACACCTGATCTG 1980
DuxG19   GGGCCTGCCCCTGTGAATGTGGAGGAAACATGGGAGCAAATGGACACAACACCTGATCTG 1980
DuxG20   GGGCCTGCCCCTGTGAATGTGGAGGAAACATGGGAGCAAATGGACACAACACCTGGTCTG 1980
DuxG21   GGGCCTGCCCCTGTGAATGTGGAGGAAACATGGGAGCAAATGGACACAACACCTGATCTG 1980
DuxG22   GGGCCTGCCCCTGTGAATGTGGAGGAAACATGGGAGCAAATGGACACAACACCTGATCTG 1980
DuxG23   GGGCCTGCCCCTGTGAATGTGGAGGAAACATGGGAGCAAATGGACACAACACCTGATCTG 1980
              **                                                *
```

```
Dux      C C T C T C A C T T C A G A A G A A T A T C A G A C T C T T C T A G A T A T G C T C T G A  2025
DuxG1    C C T C T C A C T T C A G A A G A A T A T C A G A C T C T T C T A G A T A T G C T C T G A  2025
DuxG2    C C T C T C A C T T C A G A A G A A T A T C A G A C T C T T C T A G A T A T G C T C T G A  2025
DuxG3    C C T C T C A C T T C A G A A G A A T A T C A G A C T C T T C T A G A T A T G C T C T G A  2025
DuxG4    C C T C T C A C T T C A G A A G A A T A T C A G A C T C T T C T A G A T A T G C T C T G A  2025
DuxG5    C C T C T C A C T T C A G A A G A A T A T C A G A C T C T T C T A G A T A T G C T C T G A  2025
DuxG6    C C T C T C A C T T C A G A A G A A T A T C A G A C T C T T C T A G A T A T G C T C T G A  2025
DuxG7    C C T C T C A C T T C A G A A G A A T A T C A G A C T C T T C T A G A T A T G C T C T G A  2025
DuxG8    C C T C T C A C T T C A G A A G A A T A T C A G A C T C T T C T A G A T A T G C T C T G A  2025
DuxG9    C C T C T C A C T T C A G A A G A A T A T C A G A C T C T T C T A G A T A T G C T C T G A  2025
DuxG10   C C T C T C A C T T C A G A A G A A T A T C A G A C T C T T C T A G A T A T G C T C T G A  2025
DuxG11   C C T C T C A C T T C A G A A G A A T A T C A G A C T C T T C T A G A T A T G C T C T G A  2025
DuxG12   C C T C T C A C T T C A G A A G A A T A T C A G A C T C T T C T A G A T A T G C T C T G A  2025
DuxG13   C C T C T C A C T T C A G A A G A A T A T C A G A C T C T T C T A G A T A T G C T C T G A  2025
DuxG14   C C T C T C A C T C C A G A A G A A T A T C A G A C T C T T C T A G A T A T G C T C T G A  2025
DuxG15   C C T C T C A C T T C A G A A G A A T A T C A G A C T C T T C T A G A T A T G C T C T G A  2025
DuxG16   C C T C T C A C T T C A G A A G A A T A T C A G A C T C T T C T A G A T A T G C T C T G A  2025
DuxG17   C C T C T C A C T T C A G A A G A A T A T C A G A C T C T T C T A G A T A T G C T C T G A  2025
DuxG18   C C T C T C A C T T C A G A A G A A T A T C A G A C T C T T C T A G A T A T G C T C T G A  2025
DuxG19   C C T C T C A C T T C A G A A G A A T A T C A G A C T C T T C T A G A T A T G C T C T G A  2025
DuxG20   C C T C T C A C T T C A G A A G A A T A T C A G A C T C T T C T A G A T A T G C T C T G A  2025
DuxG21   C C T C T C A C T T C A G A A G A A T A T C A G A C T C T T C T A G A T A T G C T C T G A  2025
DuxG22   C C T C T C A C T T C A G A A G A A T A T C A G A C T C T T C T A G A T A T G C T C T G A  2025
DuxG23   C C T C T C A C T T C A G A A G A A T A T C A G A C T C T T C T A G A T A T G C T C T G A  2025
                           *
```

Supplemental Figure S1. The nucleotide sequences of *Dux* family genes

The *Dux* sequence (top) was referenced from NCBI database. Red letters indicate the mutation sites when compared with the *Dux* sequence.

Supplemental Fig. S2

Fig. S2: Page 1/3

```
Dux      ------------------------------31 AGTGGTGTGGCACGGGAATCCCGGCGGCGC 60
DuxR1    ------------------------------31 AGTGGTGTGGCACGGGAATCCCGGCGGCGC 60
DuxR2    ------------------------------31 AGTGGTGTGGCACGGGAATCCCGGCGGCGC 60
DuxR3    ------------------------------31 AGTGGTGTGGCACGGGAATCCCGGCGGCGC 60
DuxR4    ------------------------------31 AGTGGTGTGGCACGGGAATCCCGGCGGCGC 60
DuxR5    ------------------------------31 AGTGGTGTGGCACGGGAATCCCGGCGGCGC 60
DuxR6    ------------------------------31 AGTGGTGTGGCACGGGAATCCCGGCGGCAC 60
DuxR7    ------------------------------31 AGTGGTGTGGCACGGGAATCCCGGCGGCGC 60
DuxR8    ------------------------------31 AGTGGTGTGGCACGGGAATCCCGGCGGCGC 60
DuxR9    ------------------------------31 AGTGGTGTGGCACGGGAATCCCGGCGGCAC 60
DuxR10   ------------------------------31 AGTGGTGTGGCACGGGAATCCCGGCGGCGC 60
DuxR11   ------------------------------31 AGTGGTGTGGCACGGGAATCCCGGCGGCGC 60
DuxR12   ------------------------------31 AGTGGTGTGGCACGGGAATCCCGGCGGCGC 60
DuxR13   ------------------------------31 AGTGGTGTGGCACGGGAATCCCGGCGGCGC 60
DuxR14   ------------------------------31 AGTGGTGTGGCACGGGAATCCCGGCGGCGC 60
                                                                      *

Dux      AGGAAGACGGTTTGGCAGGCCTGGCAAGAGCAGGCCCTGCTATCAACTTTCAAGAAGAAG 120
DuxR1    AGGAAGACGGTTTGGCAGGCCTGGCAAGAGCAGGCCCTGCTATCAACTTTCAAGAAGAAG 120
DuxR2    AGGAAGACGGTTTGGCAGGCCTGGCAAGAGCAGGCCCTGCTATCAACTTTCAAGAAGAAG 120
DuxR3    AGGAAGACGGTTTGGCAGGCCTGGCAAGAGCAGGCCCTGCTATCAACTTTCAAGAAGAAG 120
DuxR4    AGGAAGACGGTTTGGCAGGCCTGGCAAGAGCAGGCCCTGCTATCAACTTTCAAGAAGAAG 120
DuxR5    AGGAAGACGGTTTGGCAGGCCTGGCAAGAGCAGGCCCTGCTATCAACTTTCAAGAAGAAG 120
DuxR6    AGGAAGACGGTTTGGCAGGCCTGGCAAGAGCAGGCCCTGCTATCAACTTTCAAGAAGAAG 120
DuxR7    AGGAAGACGGTTTGGCAGGCCTGGCAAGAGCAGGCCCTGCTATCAACTTTCAAGAAGAAG 120
DuxR8    AGGAAGACGGTTTGGCAGGCCTGGCAAGAGCAGGCCCTGCTATCAACTTTCAAGAAGAAG 120
DuxR9    AGGAAGACGGTTTGGCAGGCCTGGCAAGAGCAGGCCCTGCTATCAACTTTCAAGAAGAAG 120
DuxR10   AGGAAGACGGTTTGGCAGGCCTGGCAAGAGCAGGCCCTGCTATCAACTTTCAAGAAGAAG 120
DuxR11   AGGAAGACGGTTTGGCAGGCCGGGCAAGAGCAGGCCCTGCTATCAACTTTCAAGAAGAAG 120
DuxR12   AGGAAGACAGTTTGGCAGGCCTGGCAAGAGCAGGCCCTGCTATCAACTTTCAAGAAGAAG 120
DuxR13   AGGAAGACGGTTTGGCAGGCCTGGCAAGAGCAGGCCCTGCTATCAACTTTCAAGAAGAAG 120
DuxR14   AGGAAGACGGTTTGGCAGGCCTGGCAAGAGCAGGCCCTGCTATCAACTTTCAAGAAGAAG 120
                 *            *

Dux      AGATACCTGAGCTTCAAGGAGAGGAAGGAGCTGGCCAAGCGAATGGGGGTCTCAGATTGC 180
DuxR1    AGATACCTGAGCTTCAAGGAGAGGAAGGAGCTGGCCAAGCGAATGGGGGTCTCAGATTGC 180
DuxR2    AGATACCTGAGCTTCAAGGAGAGGAAGGAGCTGGCCAAGCGAATGGGGGTCTCAGATTGC 180
DuxR3    AGATACCTGAGCTTCAAGGAGAGGAAGGAGCTGGCCAAGCGAATGGGGGTCTCAGATTGC 180
DuxR4    AGATACCTGAGCTTCAAGGAGAGGAAGGAGCTGGCCAAGCGAATGGGGGTCTCAGATTGC 180
DuxR5    AGATACCTGAGCTTCAAGGAGAGGAAGGAGCTGGCCAAGCAAATGGGGGTCTCAGATTGC 180
DuxR6    AGATACCTGAGCTTCAAGGAGAGGAAGGAGCTGGCCAAGCGAATGGGGGTCTCAGATTGC 180
DuxR7    AGATACCTGAGCTTCAAGGAGAGGAAGGAGCTGGCCAAGCGAATGGGGGTCTCAGATTGC 180
DuxR8    AGATACCTGAGCTTCAAGGAGAGGAAGGAGCTGGCCAAGCGAATGGGGGTCTCAGATTGC 180
DuxR9    AGATACCTGAGCTTCAAGGAGAGGAAGGAGCTGGCCAAGCGAATGGGGGTCTCAGATTGC 180
DuxR10   AGATACCTGAGCTTCAAGGAGAGGAAGGAGCTGGCCAAGCAAATGGGGGTCTCAGATTGC 180
DuxR11   AGATACCTGAGCTTCAAGGAGAGGAAGGAGCTGGCCAAGCGAATGGGGGTCTCAGATTGC 180
DuxR12   AGATACCTGAGCTTCAAGGAGAGGAAGGAGCTGGCCAAGCGAATGGGGGTCTCAGATTGC 180
DuxR13   AGATACCTGAGCTTCAAGGAGAGGAAGGAGCTGGCCAAGCGAATGGGGGTCTCAGATTGC 180
DuxR14   AGATACCTGAGCTTCAAGGAGAGGAAGGAGCTGGCCAAGCGAATGGGGGTCTCAGATTGC 180
                                                 *

Dux      CGCATCCGCGTGTGGTTTCAGAACCGCAGGAATCGCAGTGGAGAGGAGGGGCATGCCTCA 240
DuxR1    CGCATCCGCGTGTGGTTTCAGAACCGCAGGAATCGCAGTGGAGAGGAGGGGCATGCCTCA 240
DuxR2    CGCATCCGCGTGTGGTTTCAGAACCGCAGGAATCGCAGTGGAAAGGAGGGGCATGCCTCA 240
DuxR3    CGCATCCGCGTGTGGTTTCAGAACCGCAGGAATCGCAGTGGAGAGGAGGGGCATGCCTCA 240
DuxR4    CGCATCCGCGTGTGGTTTCAGAACCGCAGGAATCGCAGTGGAGAGGAGGGGCATGCCTCA 240
DuxR5    CGCATCCGCGTGTGGTTTCAGAACCGCAGGAATCGCAGTGGAGAGGAGGGGCATGCCTCA 240
DuxR6    CGCATCCGCGTGTGGTTTCAGAACCGCAGGAATCGCAGTGGAGAGGAGGGGCATGCCTCA 240
DuxR7    CGCATCCGCGTGTGGTTTCAGAACCGCAGGAATCGCAGTGGAGAGGAGGGGCATGCCTCA 240
DuxR8    CGCATCCGCGTGTGGTTTCAGAACCGCAGGAATCGCAGTGGAGAGGAGGGGCATGCCTCA 240
DuxR9    CGCATCCGCGTGTGGTTTCAGAACCGCAGGAATCGCAGTGGAGAGGAGGGGCATGCCTCA 240
DuxR10   CGCATCCGCGTGTGGTTTCAGAACCGCAGGAATCGCAGTGGAGAGGAGGGGCATGCCTCA 240
DuxR11   CGCATCCGCGTGTGGTTTCAGAACCGCAGGAATCGCAGTGGAGAGGAGGGGCATGCCTCA 240
DuxR12   CGCATCCGCGTGTGGTTTCAGAACCGCAGGAATCGCAGTGGAGAGGAGGGGCATGCCTCA 240
DuxR13   CGCATCCGCGTGTGGTTTCAGAACCGCAGGAATCGCAGTGGAGAGGAGGGGCATGCCTCA 240
DuxR14   CGCATCCGCGTGTGGTTTCAGAACCGCAGGAATCGCAGTGGAGAGGAGGGGCATGCCTCA 240
                                                   *

Dux      AAGAGGTCCATCAGAGGCTCCAGGCGGCTAGCCTCGCCACAGCTCCAGGAAGAGCTTGGA 300
DuxR1    AAGAGGTCCATCAGAGGCTCCAGGCGGCTAGCCTCGCCACAGCTCCAGGAAGAGCTTGGA 300
DuxR2    AAGAGGTCCATCAGAGGCTCCAGGCGGCTAGCCTCGCCACAGCTCCAGGAAGAGCTTGGA 300
DuxR3    AAGAGGTCCATCAGAGGCTCCAGGCGGCTAGCCTCGCCACAGCTCCAGGAAGAGCTTGGA 300
DuxR4    AAGAGGTCCATCAGAGGCTCCAGGCGGCTAGCCTCGCCACAGCTCCAGGAAGAGCTTGGA 300
DuxR5    AAGAGGTCCATCAGAGGCTCCAGGCGGCTAGCCTCGCCACAGCTCCAGGAAGAGCTTGGA 300
DuxR6    AAGAGGTCCATCAGAGGCTCCAGGCGGCTAGCCTCGCCACAGCTCCAGGAAGAGCTTGGA 300
DuxR7    AAGAGGTCCATCAGAGGCTCCAGGCGGCTAGCCTCGCCACAGCTCCAGGAAGAGCTTGGA 300
DuxR8    AAGAGGTCCATCAGAGGCTCCAGGCGGCTAGCCTCGCCACAGCTCCAGGAAGAGCTTGGA 300
DuxR9    AAGAGGTCCATCAGAGGCTCCAGGCGGCTAGCCTCGCCACAGCTCCAGGAAGAGCTTGGA 300
DuxR10   AAGAGGTCCATCAGAGGCTCCAGGCGGCTAGCCTCGCCACAGCTCCAGGAAGAGCTTGGA 300
DuxR11   AAGAGGTCCATCAGAGGCTCCAGGCGGCTAGCCTCGCCACAGCTCCAGGAAGAGCTTGGA 300
DuxR12   AAGAGGTCCATCAGAGGCTCCAGGCGGCTAGCCTCGCCACAGCTCCAGGAAGAGCTTGGA 300
DuxR13   AAGAGGTCCATCAGAGGCTCCAGGCGGCTAGCCTCGCCACAGCTCCAGGAAGAGCTTGGA 300
DuxR14   AAGAGGTCCATCAGAGGCTCCAGGCGGCTAGCCTCGCCACAGCTCCAGGAAGAGCTTGGA 300
```

Dux	TCCAGGCCACAGGGTAGAGGCATGCGCTCATCTGGCAGAAGGCCTCGCACTCGACTCACC	360
DuxR1	TCCAGGCCACAGGGTAGAGGCATGCGCTCATCTGGCAGAAGGCCTCGCACTCGACTCACC	360
DuxR2	TCCAGGCCACAGGGTAGAGGCATGCGCTCATCTGGCAGAAGGCCTCGCACTCGACTCACC	360
DuxR3	TCCAGGCCACAGGGTAGAGGCATGCGCTCATCTGGCAGAAGGCCTCGCACTCGACTCACC	360
DuxR4	TCCAGGCCACAGGGTAGAGGCATGCGCTCATCTGGCAGAAGGCCTCGCACTCGACTCACC	360
DuxR5	TCCAGGCCACAGGGTAGAGGCATGCGCTCATCTGGCAGAAGGCCTCGCACTCGACTCACC	360
DuxR6	TCCAGGCCACAGGGTAGAGGCATGCGCTCATCTGGCAGAAGGCCTCGCACTCGACTCACC	360
DuxR7	TCCAGGCCACAGGGTAGAGGCATGCGCTCATCTGGCAGAAGGCCTCGCACTCGACTCACC	360
DuxR8	TCCAGGCCACAGGGTAGAGGCATGCGCTCATCTGGCAGAAGGCCTCGCACTCGACTCACC	360
DuxR9	TCCAGGCCACAGGGTAGAGGCATGCGCTCATCTGGCAGAAGGCCTCGCACTCGACTCACC	360
DuxR10	TCCAGGCCAC**G**GGGTAGAGGCATGCGCTCATCTGGCAGAAGGCCTCGCACTCGACTCACC	360
DuxR11	TCCAGGCCACAGGGTAGAGGCATGCGCTCATCTGGCAGAAGGCCTCGCACTCGACTCACC	360
DuxR12	TCCAGGCCACAGGGTAGAGGCATGCGCTCATCTGGCAGAAGGCCTCGCACTCGACTCACC	360
DuxR13	TCCAGGCCACAGGGTAGAGGCATGCGCTCATCTGGCAGAAGGCCTCGCACTCGACTCACC	360
DuxR14	TCCAGGCCACAGGGTAGAGGCATGCGCTCATCTGGCAGAAGGCCTCGCACTCGACTCACC	360
	*	

Dux	TCGCTACAGCTCAGGATCCTAGGGCAAGCCTTTGAGAGGAACCCACGACCAGGCTTTGCT	420
DuxR1	TCGCTACAGCTCAGGATCCTAGGGCAAGCCTTTGAGAGGAACCCACGACCAGGCTTTGCT	420
DuxR2	TCGCTACAGCTCAGGATCCTAGGGCAAGCCTTTGAGAGGAACCCACGACCAGGCTTTGCT	420
DuxR3	TCGCTACAGCTCAGGATCCTAGGGCAAGCCTTTGAGAGGAACCCACGACCAGGCTTTGCT	420
DuxR4	TCGCTACAGCTCAGGATCCTAGGGCAAGCCTTTGAGAGGAACCCACGACCAGGCTTTGCT	420
DuxR5	TCGCTACAGCTCAGGATCCTAGGGCAAGCCTTTGAGAGGAA**T**CCACGACCAGGCTTTGCT	420
DuxR6	TCGCTACAGCTCAGGATCCTAGGGCAAGCCTTTGAGAGGAACCCACGACCAGGCTTTGCT	420
DuxR7	TCGCTACAGCTCAGGATCCTAGGGCAAGCCTTTGAGAGGAACCCACGACCAGGCTTTGCT	420
DuxR8	TCGCTACAGCTCAGGATCCTAGGGCAAGCCTTTGAGAGGAACCCACGACCAGGCTTTGCT	420
DuxR9	TCGCTACAGCTCAGGATCCTAGGGCAAGCCTTTGAGAGGAACCCACGACCAGGCTTTGCT	420
DuxR10	TCGCTACAGCTCAGGATCCTAGGGCAAGCCTTTGAGAGGAA**T**CCACGACCAGGCTTTGCT	420
DuxR11	TCGCTACAGCTCAGGATCCTAGGGCAAGCCTTTGAGAGGAACCCACGACCAGGCTTTGCT	420
DuxR12	TCGCTACAGCTCAGGATCCTAGGGCAAGCCTTTGAGAGGAACCCACGACCAGGCTTTGCT	420
DuxR13	TCGCTACAGCTCAGGATCCTAGGGCAAGCCTTTGAGAGGAACCCACGACCAGGCTTTGCT	420
DuxR14	TCGCTACAGCTCAGGATCCTAGGGCAAGCCTTTGAGAGGAACCCACGACCAGGCTTTGCT	420
	*	

Dux	ACCAGGGAGGAGCTGGCGCGTGACACAGGGTTGCCCGAGGACACGATCCACATATGGTTT	480
DuxR1	ACCAGGGAGGAGCTGGCGCGTGACACAGGGTTGCCCGAGGACACGATCCACATATGGTTT	480
DuxR2	ACCAGGGAGGAGCTGGCGCGTGACACAGGGTTGCCCGAGGACACGATCCACATATGGTTT	480
DuxR3	ACCAGGGAGGAGCTGGCGCGTGACACAGGG**A**TGCCCGAGGACACGATCCACATATGGTTT	480
DuxR4	ACCAGGGAGGAGCTGGCGCGTGACACAGGGTTGCCCGAGGACACGATCCACATATGGTTT	480
DuxR5	ACCAGGGAGGAGCTGGCGCGTGACACAGGGTTGCCCGAGGACACGATCCACATATGGTTT	480
DuxR6	ACCAGGGAGGAGCTGGCGCGTGACACAGGGTTGCCCGAGGACACGATCCACATATGGTTT	480
DuxR7	ACCAGGGAGGAGCTGGCGCGTGACACAGGGTTGCCCGAGGACACGATCCACATATGGTTT	480
DuxR8	ACCAGGGAGGAGCTGGCGCGTGACACAGGGTTGCCCGAGGACACGATCCACATATGGTTT	480
DuxR9	ACCAGGGAGGAGCTGGCGCGTGACACAGGGTTGCCCGAGGACACGATCCACATATGGTTT	480
DuxR10	ACCAGGGAGGAGCTGGCGCGTGACACAGGGTTGCCCGAGGACACGATCCACATATGGTTT	480
DuxR11	ACCAGGGAGGAGCTGGCGCGTGACACAGGGTTGCCCGAGGACACGATCCACATATGGTTT	480
DuxR12	ACCAGGGAGGAGCTGGCGCGTGACACAGGGTTGCCCGAGGACACGATCCACATATGGTTT	480
DuxR13	ACCAGGGAGGAGCTGGCGCGTGACACAGGGTTGCCCGAGGACACGATCCACATATGGTTT	480
DuxR14	ACCAGGGAGGAGCTGGCGCGTGACACAGGGTTGCCCGAGGACACGATCCACATATGGTTT	480
	*	

Dux	CAAAACCGAAGAGCTCGGCGGCGCCACAGGAGGGGCAGGCCCACAGCTCAAGATCAAGAC	540
DuxR1	CAAAACCGAAGAGCTCGGCGGCGCCACAGGAGGGGCAGGCCCACAGCTCAAGATCAAGAC	540
DuxR2	CAAAACCGAAGAGCTCGGCGGCGCCACAGGAGGGGCAGGCCCACAGCTCAAGATCAAGAC	540
DuxR3	CAAAACCGAAGAGCTCGGCGGCGCCACAGGAGGGGCAGGCCCACAGCTCAAGATCAAGAC	540
DuxR4	CAAAACCGAAGAGCTCGGCGGCGCCACAGGAGGGGCAGGCCCACAGCTCAAGATCAAGAC	540
DuxR5	CAAAACCGAAGAGCTCGGCGGCGCCACAGGAGGGGCAGGCCCACAGCTCAAGATCAAGAC	540
DuxR6	CAAAACCGAAGAGCTCGGCGGCGCCACAGGAGGGGCAGGCCCACAGCTCAAGATCAAGAC	540
DuxR7	CAAAACCGAAGAGCTCGGCGGCGCCACAG**A**AGGGGCAGGCCCACAGCTCAAGATCAAGAC	540
DuxR8	CAAAACCGAAGAGCTCGGCGGCGCCACAGGAGGGGCAGGCCCACAGCTCAAGATCAAGAC	540
DuxR9	CAAAACCGAAGAGCTCGGCGGCGCCACAGGAGGGGCAGGCCCACAGCTCAAGATCAAGAC	540
DuxR10	CAAAACCGAAGAGCTCGGCGGCGCCACAGGAGGGGCAGGCCCACAGCTCAAGATCAAGAC	540
DuxR11	CAAAACCGAAGAGCTCGGCGGCGCCACAGGAGGGGCAGGCCCACAGCTCAAGATCAAGAC	540
DuxR12	CAAAACCGAAGAGCTCGGCGGCGCCACAGGAGGGGCAGGCCCACAGCTCAAGATCAAGAC	540
DuxR13	CAAAACCGAAGAGCTCGGCGGCGCCACAG**A**AGGGGCAGGCCCACAGCTCAAGATCAAGAC	540
DuxR14	CAAAACCGAAGAGCTCGGCGGCGCCACAGGAGGGGCAGGCCCACAGCTCAAGATCAAGAC	540
	*	

Dux	TTGCTGGCGTCACAAGGGTCGGATGGGGCCCCTGCAGGTCCGGAAGGCAGAGAGCGTGAA	600
DuxR1	TTGCTGGCGTCACAAGGGTCGGATGGGGCCCCTGCAGGTCCGGAAGGCAGAGAGCGTGAA	600
DuxR2	TTGCTGGCGTCACAAGGGTCGGATGGGGCCCCTGCAGGTCCGGAAGGCAGAGAGCGTGAA	600
DuxR3	TTGCTGGCGTCACAAGGGTCGGATGGGGCCCCTGCAGGTCCGGAAGGCAGAGAGCGTGAA	600
DuxR4	TTGCTGGCGTCACAAGGGTCGGATGGGGCCCCTGCAGGTCCGGAAGGCAGAGAGCGTGAA	600
DuxR5	TTGCTGGCGTCACAAGGGTCGGATGGGGCCCCTGCAGGTCCGGAAGGCAGAGAGCGTGAA	600
DuxR6	TTGCTGGCGTCACAAGGGTCGGATGGGGCCCCTGCAGGTCCGGAAGGCAGAGAGCGTGAA	600
DuxR7	TTGCTGGC**A**TCACAAGGGTCGGATGGGGCCCCTGCAGGTCCGGAAGGCAGAGAGCGTGAA	600
DuxR8	TTGCTGG**T**GTCACAAGGGTCGGATGGGGCCCCTGCAGGTCCGGAAGGCAGAGAGCGTGAA	600
DuxR9	TTGCTGGCGTCACAAGGGTCGGATGGGGCCCCTGCAGGTCCGGAAGGCAGAGAGCGTGAA	600
DuxR10	TTGCTGGCGTCACAAGGGTCGGATGGGGCCCCTGCAGGTCCGGAAGGCAGAGAGCGTGAA	600
DuxR11	TTGCTGGCGTCACAAGGGTCGGATGGGGCCCCTGCAGGTCCGGAAGGCAGAGAGCGTGAA	600
DuxR12	TTGCTGGCGTCACAAGGGTCGGATGGGGCCCCTGCAGGTCCGGAAGGCAGAGAGCGTGAA	600
DuxR13	TTGCTGGCGTCACAAGGGTCGGATGGGGCCCCTGCAGGTCCGGAAGGCAGAGAGCGTGAA	600
DuxR14	TTGCTGGCGTCACAAGGGTCGGATGGGGCCCCTGCAGGTCCGGAAGGCAGAGAGCGTGAA	600
	* *	

```
Dux     GGTGCCCAGGAGAACTTGTTGCCACAGGAAGAAGCAGGAAGTACGGGCATGGATACCTCG 660
DuxR1   GGTGCCCAGGAGAACTTGTTGCCACAGGAAGAAGCAGGAAGTACGGGCATGGATACCTCG 660
DuxR2   GGTGCCCAGGAGAACTTGTTGCCACAGGAAGAAGCAGGAAGTACGGGCATGGATACCTCG 660
DuxR3   GGTGCCCAGGAGAACTTGTTGCCACAGGAAGAAGCAGGAAGTACGGGCATGGATACCTCG 660
DuxR4   GGTGCCCAGGAGAACTTGTTGCCACAGGAAGAAGCAGGAAGTACGGGCATGGATACCTCG 660
DuxR5   GGTGCCCAGGAGAACTTGTTGCCACAGGAAGAAGCAGGAAGTACGGGCATGGATACCTCG 660
DuxR6   GGTGCCCAGGAGAACTTGTTGCCACAGGAAGAAGCAGGAAGTACGGGCATGGATACCTCG 660
DuxR7   GGTGCCCAGGAGAACTTGTTGCCACAGGAAGAAGCAGGAAGTACGGGGATGGATACCTCG 660
DuxR8   GGTGCCCAGGAGAACTTGTTGCCACAGGAAGAAGCAGGAAGTACGGGCATGGATACCTCG 660
DuxR9   GGTGCCCAGGAGAACTTGTTGCCACAGGAAGAAGCAGGAAGTACGGGCATGGATACCTCG 660
DuxR10  GGTGCCCAGGAGAACTTGTTGCCACAGGAAGAAGCAGGAAGTACGGGCATGGATACCTCG 660
DuxR11  GGTGCCCAGGAGAACTTGTTGCCACAGGAAGAAGCAGGAAGTACCGGCATGGATACCTCG 660
DuxR12  GGTGCCCAGGAGAACTTGTTGCCACAGGAAGAAGCAGGAAGTACGGGCATGGATACCTCG 660
DuxR13  GGTGCCCAGGAGAACTTGTTGCCACAGGAAGAAGCAGGAAGTACGGGCATGGATACCTCG 660
DuxR14  GGTGCCCAGGAGAACTTGTTGCCACAGGAAGAAGCAGAAAGTACGGGCATGGATACCTCG 660
                                             *      *  *

Dux     AGCCCTAGCGACTTGCCCTCCTTCTGCGGAGAGTCCCAGCCTTTCCAAGTGGCACAGCCC 720
DuxR1   AGCCCTAGCGACTTGCCCTCCTTCTGCGGAGAGTCCCAGCCTTTCCAAGTGGCACAGCCC 720
DuxR2   AGCCCTAGCGACTTGCCCTCCTTCTGCGGAGAGTCCCAGCCTTTCCAAGTGGCACAGCCC 720
DuxR3   AGCCCTAGCGACTTGCCCTCCTTCTGCGGAGAGTCCCAGCCTTTCCAAGTGGCACAGCCC 720
DuxR4   AGCCCTAGCGACTTGCCCTCCTTCTGCGGAGAGTCCCAGCCTTTCCAAGTGGCACAGCCC 720
DuxR5   AGCCCTAGCGACTTGCCCTCCTTCTGCGGAGAGTCCCAGCCTTTCCAAGTGGCACAGCCC 720
DuxR6   AGCCCTAGCGACTTGCCCTCCTTCTGCGGAGAGTCCCAGCCTTTCCAAGTGGCACAGCCC 720
DuxR7   AGCCCTAGCGACTTGCCCTCCTTCTGCGGAGAGTCCCAGCCTTTCCAAGTGGCACAGCCC 720
DuxR8   AGCCCTAGCGACTTGCCCTCCTTCTGCGGAGAGTCCCAGCCTTTCCAAGTGGCACAGCCC 720
DuxR9   AGCCCTAGCGACTTGCCCTCCTTCTGCGGAGAGTCCCAGCCTTTCCAAGTGGCACAGCCC 720
DuxR10  AGCCCTAGCGACTTGCCCTCCTTCTGCGGAGAGTCCCAGCCTTTCCAAGTGGCACAGCCC 720
DuxR11  AGCCCTAGCGACTTGCCCTCCTTCTGCGGAGAGTCCCAGCCTTTCCAAGTGGCACAGCCC 720
DuxR12  AGCCCTAGCGACTTGCCCTCCTTCTGCGGAGAGTCCCAGCCTTTCCAAGTGGCACAGCCC 720
DuxR13  AGCCCTAGCGACTTGCCCTCCTTCTGCGGAGAGTCCCAGCCTTTCCAAGTGGCACAGCCC 720
DuxR14  AGCCCTAGCGACTTGCCCTCCTTCTGCGGAGAGTCCCAGCCTTTCCAAGTGGCACAGCCC 720

Dux     CGTGGAGCAGGCCAACAAGAGGCCCCCACTCGAGCAGGCAACGCAGGCTCTCTGGAACCC 780
DuxR1   CGTGGAGCAGGCCAACAAGAGGCCCCCACTCAAGCAGGCAACGCAGGCTCTCTGGAACCC 780
DuxR2   CGTGGAGCAGGCCAACAAGAGGCCCCCACTCAAGCAGGCAACGCAGGCTCTCTGGAACCC 780
DuxR3   CGTGGAGCAGGCCAACAAGAGGCCCCCACTCAAGCAGGCAACGCAGGCTCTCTGGAACCC 780
DuxR4   CGTGGAGCAGGCCAACAAGAGGCCCCCACTCGAGCAGGCAACGCAGGCTCTCTGGAAACC 780
DuxR5   CGTGGAGCAGGCCAACAAGAGGCCCCCACTCAAGCAGGCAACGCAGGCTCTCTGGAACCC 780
DuxR6   CGTGGAGCAGGCCAACAAGAGGCCCCCACTCGAGCAGGCAACGCAGGCTCTCTGGAACCC 780
DuxR7   CGTGGAGCAGGCCAACAAGAGGCCCCCACTCAAGCAGGCAACGCAGGCTCTCTGGAACCC 780
DuxR8   CGTGGAGCAGGCCAACAAGAGGCCCCCACTCGAGCAGGCAACGCAGGCTCTCTGGAACCC 780
DuxR9   CGTGGAGCAGGCCAACAAGAGGCCCCCACTCAAGCAGGCAACGCAGGCTCTCTGGAACCC 780
DuxR10  CGTGGAGCAGGCCAACAAGAGGCCCCCACTCGAGCAGGCAACGCAGGCTCTCTGGAACCC 780
DuxR11  CGTGGAGCAGGCCAACAAGAGGCCCCCACTCAAGCAGGCAACGCAGGCTCTCTGGAACCC 780
DuxR12  CGTGGAGCAGGCCAACAAGAGGCCCCCACTCAAGCAGGCAACGCAGGCTCTCTGGAACCC 780
DuxR13  CGTGGAGCAGGCCAACAAGAGGCCCCCACTCGAGCAGGCAACGCAGGCTCTCTGGAAACC 780
DuxR14  CGTGGAGCAGGCCAACAAGAGGCCCCCACTCGAGCAGGCAACGCAGGCTCTCTGGAACCC 780
                                       *                         *

Dux     CTCCTTGATCAGCTGCTGGATGAAGTCCAAGTAGAAGAGCCTGCTCCAGCCCCTCTGAAT 840
DuxR1   CTCCTTGATCAGCTGCTGGATGAAGTCCAAGTAGAAGAGCCTGCTCCAGCCCCTCTGAAT 840
DuxR2   CTCCTTGATCAGCTGCTGGATGAAGTCCAAGTAGAAGAGCCTGCTCCAGCCCCTCTGAAT 840
DuxR3   CTCCTTGATCAGCTGCTGGATGAAGTCCAAGTAGAAGAGCCTGCTCCAGCCCCTCTGAAT 840
DuxR4   CTCCTTGATCAGCTGCTGGATGAAGTCCAAGTAGAAGAGCCTGCTCCAGCCCCTCTGAAT 840
DuxR5   CTCCTTGATCAGCTGCTGGATGAAGTCCAAGTAGAAGAGCCTGCTCCAGCCCCTCTGAAT 840
DuxR6   CTCCTTGATCAGCTGCTGGATGAAGTCCAAGTAGAAGAGCCTGCTCCAGCCCCTCTGAAT 840
DuxR7   CTCCTTGATCAGCTGCTGGATGAAGTCCAAGTAGAAGAGCCTGCTCCAGCCCCTCTGAAT 840
DuxR8   CTCCTTGATCAGCTGCTGGATGAAGTCCAAGTAGAAGAGCCTGCTCCAGCCCCTCTGAAT 840
DuxR9   CTCCTTGATCAGCTGCTGGATGAAGTCCAAGTAGAAGAGCCTGCTCCAGCCCCTCTGAAT 840
DuxR10  CTCCTTGATCAGCTGCTGGATGAAGTCCAAGTAGAAGAGCCTGCTCCAGCCCCTCTGAAT 840
DuxR11  CTCCTTGATCAGCTGCTGGATGAAGTCCAAGTAGAAGAGCCTGCTCCAGCCCCTCTGAAT 840
DuxR12  CTCCTTGATCAGCTGCTGGATGAAGTCCAAGTAGAAGAGCCTGCTCCAGCCCCTCTGAAT 840
DuxR13  CTCCTTGATCAGCTGCTGGATGAAGTCCAAGTAGAAGAGCCTGCTCCAGCCCCTCTGAAT 840
DuxR14  CTCCTTGATCAGCTGCTGGATGAAGTCCAAGTAGAAGAGCCTGCTCCAGCCCCTCTGAAT 840

Dux     TTGGATGGAGACCCTGGTGGCAGGGTGCATGAAGGTTCCCAGGAGAGCT 889
DuxR1   TTGGATGGAGACCCTGGTGGCAGGGTGCATGAAGGTTCCCAGGAGAGCT 889
DuxR2   TTGGATGGAGACCCTGGTGGCAGGGTGCATGAAGGTTCCCAGGAGAGCT 889
DuxR3   TTGGATGGAGACCCTGGTGGCAGGGTGCATGAAGGTTCCCAGGAGAGCT 889
DuxR4   TTGGATGGAGACCCTGGTGGCAGGGTGCATGAAGGTTCCCAGGAGAGCT 889
DuxR5   TTGGATGGAGACCCTGGTGGCAGGGTGCATGAAGGTTCCCAGGAGAGCT 889
DuxR6   TTGGATGGAGACCCTGGTGGCAGGGTGCATGAAGGTTCCCAGGAGAGCT 889
DuxR7   TTGGATGGAGACCCTGGTGGCAGGGTGCATGAAGGTTCCCAGGAGAGCT 889
DuxR8   TTGGATGGAGACCCTGGTGGCAGGGTGCATGAAGGTTCCCAGGAGAGCT 889
DuxR9   TTGGATGGAGACCCTGGTGGCAGGGTGCATGAAGGTTCCCAGGAGAGCT 889
DuxR10  TTGGATGGAGACCCTGGTGGCAGGGTGCATGAAGGTTCCCAGGAGAGCT 889
DuxR11  TTGGATGGAGACCCTGGTGGCAGGGTGTATGAAGGTTCCCAGGAGAGCT 889
DuxR12  TTGGATGGAGACCCTGGTGGCAGGGTGCATGAAGGTTCCCAGGAGAGCT 889
DuxR13  TTGGATGGAGACCCTGGTGGCAGGGTGCATGAAGGTTCCCAGGAGAGCT 889
DuxR14  TTGGATGGAGACCCTGGTGGCAGGGTGCATGAAGGTTCCCAGGAGAGCT 889
                                    *
```

Supplemental Figure S2. Nucleotide sequences of *Dux* family mRNA expressed in early preimplantation embryos

The sequences of *Dux* family transcripts (31 to 889 bps) are aligned. The *Dux* sequence (top) was referenced from NCBI database. Red letters indicate the mutation sites when compared with the *Dux* sequence.

Supplemental Fig. S3

Dux	11	SGVARESRRRRKTVWQAWQEQALLSTFKKKRYLSFKERKELAKRMGVSDC	60
DuxR1	11	SGVARESRRRRKTVWQAWQEQALLSTFKKKRYLSFKERKELAKRMGVSDC	60
DuxR2	11	SGVARESRRRRKTVWQAWQEQALLSTFKKKRYLSFKERKELAKRMGVSDC	60
DuxR3	11	SGVARESRRRRKTVWQAWQEQALLSTFKKKRYLSFKERKELAKRMGVSDC	60
DuxR4	11	SGVARESRRRRKTVWQAWQEQALLSTFKKKRYLSFKERKELAKRMGVSDC	60
DuxR5	11	SGVARESRRRRKTVWQAWQEQALLSTFKKKRYLSFKERKELAK**Q**MGVSDC	60
DuxR6	11	SGVARESRR**H**RKTVWQAWQEQALLSTFKKKRYLSFKERKELAKRMGVSDC	60
DuxR7	11	SGVARESRRRRKTVWQAWQEQALLSTFKKKRYLSFKERKELAKRMGVSDC	60
DuxR8	11	SGVARESRRRRKTVWQAWQEQALLSTFKKKRYLSFKERKELAKRMGVSDC	60
DuxR9	11	SGVARESRR**H**RKTVWQAWQEQALLSTFKKKRYLSFKERKELAKRMGVSDC	60
DuxR10	11	SGVARESRRRRKTVWQAWQEQALLSTFKKKRYLSFKERKELAK**Q**MGVSDC	60
DuxR11	11	SGVARESRRRRKTVWQA**G**QEQALLSTFKKKRYLSFKERKELAKRMGVSDC	60
DuxR12	11	SGVARESRRRRKTVWQAWQEQALLSTFKKKRYLSFKERKELAKRMGVSDC	60
DuxR13	11	SGVARESRRRRKTVWQAWQEQALLSTFKKKRYLSFKERKELAKRMGVSDC	60
DuxR14	11	SGVARESRRRRKTVWQAWQEQALLSTFKKKRYLSFKERKELAKRMGVSDC	60
		* * *	

Dux	RIRVWFQNRRNRSGEEGHASKRSIRGSRRLASPQLQEELGSRPQGRGMRSSGRRPRTRLT	120
DuxR1	RIRVWFQNRRNRSGEEGHASKRSIRGSRRLASPQLQEELGSRPQGRGMRSSGRRPRTRLT	120
DuxR2	RIRVWFQNRRNRSG**K**EGHASKRSIRGSRRLASPQLQEELGSRPQGRGMRSSGRRPRTRLT	120
DuxR3	RIRVWFQNRRNRSGEEGHASKRSIRGSRRLASPQLQEELGSRPQGRGMRSSGRRPRTRLT	120
DuxR4	RIRVWFQNRRNRSGEEGHASKRSIRGSRRLASPQLQEELGSRPQGRGMRSSGRRPRTRLT	120
DuxR5	RIRVWFQNRRNRSGEEGHASKRSIRGSRRLASPQLQEELGSRPQGRGMRSSGRRPRTRLT	120
DuxR6	RIRVWFQNRRNRSGEEGHASKRSIRGSRRLASPQLQEELGSRPQGRGMRSSGRRPRTRLT	120
DuxR7	RIRVWFQNRRNRSGEEGHASKRSIRGSRRLASPQLQEELGSRPQGRGMRSSGRRPRTRLT	120
DuxR8	RIRVWFQNRRNRSGEEGHASKRSIRGSRRLASPQLQEELGSRPQGRGMRSSGRRPRTRLT	120
DuxR9	RIRVWFQNRRNRSGEEGHASKRSIRGSRRLASPQLQEELGSRPQGRGMRSSGRRPRTRLT	120
DuxR10	RIRVWFQNRRNRSGEEGHASKRSIRGSRRLASPQLQEELGSRP**R**GRGMRSSGRRPRTRLT	120
DuxR11	RIRVWFQNRRNRSGEEGHASKRSIRGSRRLASPQLQEELGSRPQGRGMRSSGRRPRTRLT	120
DuxR12	RIRVWFQNRRNRSGEEGHASKRSIRGSRRLASPQLQEELGSRPQGRGMRSSGRRPRTRLT	120
DuxR13	RIRVWFQNRRNRSGEEGHASKRSIRGSRRLASPQLQEELGSRPQGRGMRSSGRRPRTRLT	120
DuxR14	RIRVWFQNRRNRSGEEGHASKRSIRGSRRLASPQLQEELGSRPQGRGMRSSGRRPRTRLT	120
	* *	

Dux	SLQLRILGQAFERNPRPGFATREELARDTGLPEDTIHIWFQNRRARRRHRRGRPTAQDQD	180
DuxR1	SLQLRILGQAFERNPRPGFATREELARDTGLPEDTIHIWFQNRRARRRHRRGRPTAQDQD	180
DuxR2	SLQLRILGQAFERNPRPGFATREELARDTGLPEDTIHIWFQNRRARRRHRRGRPTAQDQD	180
DuxR3	SLQLRILGQAFERNPRPGFATREELARDTG**M**PEDTIHIWFQNRRARRRHRRGRPTAQDQD	180
DuxR4	SLQLRILGQAFERNPRPGFATREELARDTGLPEDTIHIWFQNRRARRRHRRGRPTAQDQD	180
DuxR5	SLQLRILGQAFERNPRPGFATREELARDTGLPEDTIHIWFQNRRARRRHRRGRPTAQDQD	180
DuxR6	SLQLRILGQAFERNPRPGFATREELARDTGLPEDTIHIWFQNRRARRRHRRGRPTAQDQD	180
DuxR7	SLQLRILGQAFERNPRPGFATREELARDTGLPEDTIHIWFQNRRARRRHRRGRPTAQDQD	180
DuxR8	SLQLRILGQAFERNPRPGFATREELARDTGLPEDTIHIWFQNRRARRRHRRGRPTAQDQD	180
DuxR9	SLQLRILGQAFERNPRPGFATREELARDTGLPEDTIHIWFQNRRARRRHRRGRPTAQDQD	180
DuxR10	SLQLRILGQAFERNPRPGFATREELARDTGLPEDTIHIWFQNRRARRRHRRGRPTAQDQD	180
DuxR11	SLQLRILGQAFERNPRPGFATREELARDTGLPEDTIHIWFQNRRARRRHRRGRPTAQDQD	180
DuxR12	SLQLRILGQAFERNPRPGFATREELARDTGLPEDTIHIWFQNRRARRRHRRGRPTAQDQD	180
DuxR13	SLQLRILGQAFERNPRPGFATREELARDTGLPEDTIHIWFQNRRARRRHRRGRPTAQDQD	180
DuxR14	SLQLRILGQAFERNPRPGFATREELARDTGLPEDTIHIWFQNRRARRRHRRGRPTAQDQD	180
	*	

Dux	LLASQGSDGAPAGPEGREREGAQENLLPQEEAGSTGMDTSSPSDLPSFCGESQPFQVAQP	240
DuxR1	LLASQGSDGAPAGPEGREREGAQENLLPQEEAGSTGMDTSSPSDLPSFCGESQPFQVAQP	240
DuxR2	LLASQGSDGAPAGPEGREREGAQENLLPQEEAGSTGMDTSSPSDLPSFCGESQPFQVAQP	240
DuxR3	LLASQGSDGAPAGPEGREREGAQENLLPQEEAGSTGMDTSSPSDLPSFCGESQPFQVAQP	240
DuxR4	LLASQGSDGAPAGPEGREREGAQENLLPQEEAGSTGMDTSSPSDLPSFCGESQPFQVAQP	240
DuxR5	LLASQGSDGAPAGPEGREREGAQENLLPQEEAGSTGMDTSSPSDLPSFCGESQPFQVAQP	240
DuxR6	LLASQGSDGAPAGPEGREREGAQENLLPQEEAGSTGMDTSSPSDLPSFCGESQPFQVAQP	240
DuxR7	LLASQGSDGAPAGPEGREREGAQENLLPQEEAGSTGMDTSSPSDLPSFCGESQPFQVAQP	240
DuxR8	LL**V**SQGSDGAPAGPEGREREGAQENLLPQEEAGSTGMDTSSPSDLPSFCGESQPFQVAQP	240
DuxR9	LLASQGSDGAPAGPEGREREGAQENLLPQEEAGSTGMDTSSPSDLPSFCGESQPFQVAQP	240
DuxR10	LLASQGSDGAPAGPEGREREGAQENLLPQEEAGSTGMDTSSPSDLPSFCGESQPFQVAQP	240
DuxR11	LLASQGSDGAPAGPEGREREGAQENLLPQEEAGSTGMDTSSPSDLPSFCGESQPFQVAQP	240
DuxR12	LLASQGSDGAPAGPEGREREGAQENLLPQEEAGSTGMDTSSPSDLPSFCGESQPFQVAQP	240
DuxR13	LLASQGSDGAPAGPEGREREGAQENLLPQEEAGSTGMDTSSPSDLPSFCGESQPFQVAQP	240
DuxR14	LLASQGSDGAPAGPEGREREGAQENLLPQEEA**E**STGMDTSSPSDLPSFCGESQPFQVAQP	240
	* *	

Dux	RGAGQQEAPTRAGNAGSLEPLLDQLLDEVQVEEPAPAPLNLDGDPGGRVHEGSQES	296
DuxR1	RGAGQQEAPT**Q**AGNAGSLEPLLDQLLDEVQVEEPAPAPLNLDGDPGGRVHEGSQES	296
DuxR2	RGAGQQEAPT**Q**AGNAGSLEPLLDQLLDEVQVEEPAPAPLNLDGDPGGRVHEGSQES	296
DuxR3	RGAGQQEAPT**Q**AGNAGSLEPLLDQLLDEVQVEEPAPAPLNLDGDPGGRVHEGSQES	296
DuxR4	RGAGQQEAPTRAGNAGSLE**T**LLDQLLDEVQVEEPAPAPLNLDGDPGGRVHEGSQES	296
DuxR5	RGAGQQEAPT**Q**AGNAGSLEPLLDQLLDEVQVEEPAPAPLNLDGDPGGRVHEGSQES	296
DuxR6	RGAGQQEAPTRAGNAGSLEPLLDQLLDEVQVEEPAPAPLNLDGDPGGRVHEGSQES	296
DuxR7	RGAGQQEAPT**Q**AGNAGSLEPLLDQLLDEVQVEEPAPAPLNLDGDPGGRVHEGSQES	296
DuxR8	RGAGQQEAPTRAGNAGSLEPLLDQLLDEVQVEEPAPAPLNLDGDPGGRVHEGSQES	296
DuxR9	RGAGQQEAPT**Q**AGNAGSLEPLLDQLLDEVQVEEPAPAPLNLDGDPGGRVHEGSQES	296
DuxR10	RGAGQQEAPTRAGNAGSLEPLLDQLLDEVQVEEPAPAPLNLDGDPGGRVHEGSQES	296
DuxR11	RGAGQQEAPT**Q**AGNAGSLEPLLDQLLDEVQVEEPAPAPLNLDGDPGGRV**Y**EGSQES	296
DuxR12	RGAGQQEAPT**Q**AGNAGSLEPLLDQLLDEVQVEEPAPAPLNLDGDPGGRVHEGSQES	296
DuxR13	RGAGQQEAPTRAGNAGSLE**T**LLDQLLDEVQVEEPAPAPLNLDGDPGGRVHEGSQES	296
DuxR14	RGAGQQEAPTRAGNAGSLEPLLDQLLDEVQVEEPAPAPLNLDGDPGGRVHEGSQES	296
	* * *	

Supplemental Figure S3. ***A*****mino acid sequences of** ***Dux*** **families**

The sequences of *Dux* family mRNAs in Supplemental Figure 2 were translated to those of amino acids. Red letters indicate the mutation sites when compared with the *Dux* sequence. Although the nucleotide sequences were different, DuxR1, DuxR7 and R12 code the same amino acid sequence, and so do DuxR4 and DuxR13.

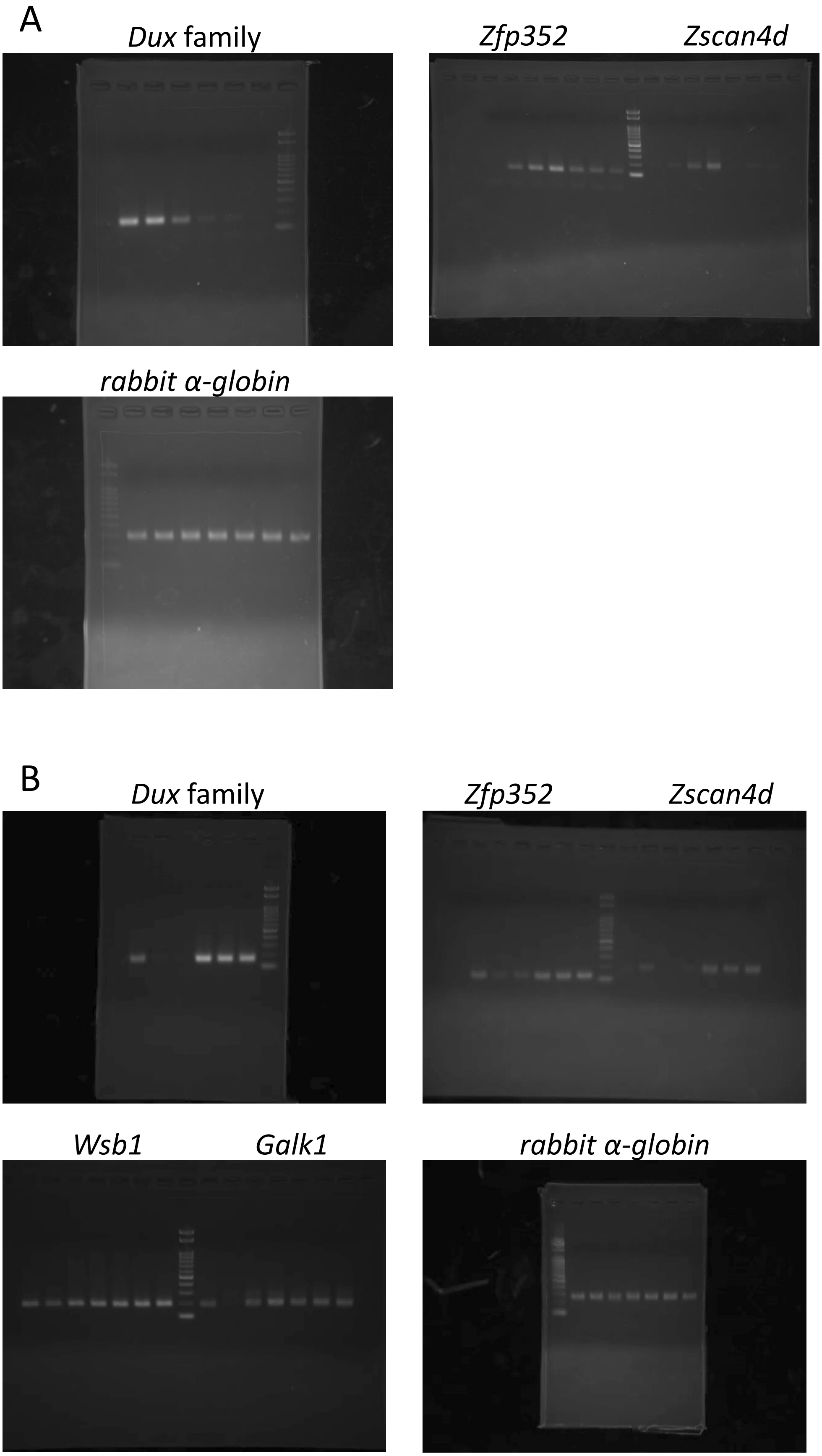

Supplemental Figure S4. Original electrophoresis images

These are uncropped images of electrophoresis gels in Fig. 1A **(A)** and Fig. 3A **(B)**.
